# Structural and mechanistic basis of capsule *O*-acetylation in *Neisseria meningitidis* serogroup A

**DOI:** 10.1038/s41467-020-18464-y

**Published:** 2020-09-18

**Authors:** Timm Fiebig, Johannes T. Cramer, Andrea Bethe, Petra Baruch, Ute Curth, Jana I. Führing, Falk F. R. Buettner, Ulrich Vogel, Mario Schubert, Roman Fedorov, Martina Mühlenhoff

**Affiliations:** 1 Institute of Clinical Biochemistry, Hannover Medical School, Hannover, Germany; 2 Institute for Virology, Hannover Medical School, Hannover, Germany; 3 Institute for Biophysical Chemistry, Hannover Medical School, Hannover, Germany; 4 Fraunhofer International Consortium for Anti-Infective Research (iCAIR), Hannover, Germany; 5 Institute for Hygiene and Microbiology, University of Würzburg, Würzburg, Germany; 6 Department of Biosciences, University of Salzburg, Salzburg, Austria

**Keywords:** Polysaccharides, Enzyme mechanisms, Solution-state NMR, Conjugate vaccines, X-ray crystallography

## Abstract

*O*-Acetylation of the capsular polysaccharide (CPS) of *Neisseria meningitidis* serogroup A (NmA) is critical for the induction of functional immune responses, making this modification mandatory for CPS-based anti-NmA vaccines. Using comprehensive NMR studies, we demonstrate that *O*-acetylation stabilizes the labile anomeric phosphodiester-linkages of the NmA-CPS and occurs in position C3 and C4 of the *N*-acetylmannosamine units due to enzymatic transfer and non-enzymatic ester migration, respectively. To shed light on the enzymatic transfer mechanism, we solved the crystal structure of the capsule *O*-acetyltransferase CsaC in its apo and acceptor-bound form and of the CsaC-H228A mutant as trapped acetyl-enzyme adduct in complex with CoA. Together with the results of a comprehensive mutagenesis study, the reported structures explain the strict regioselectivity of CsaC and provide insight into the catalytic mechanism, which relies on an unexpected Gln-extension of a classical Ser-His-Asp triad, embedded in an α/β-hydrolase fold.

## Abstract (web summary)

*Neisseria meningitidis* capsular polysaccharide (CPS) is a major virulence factor and vaccine formulations against Neisseria meningitidis serogroup A (NmA) contain *O*-acetylated CPS. Here, the authors provide mechanistic insights into CPS *O*-acetylation in NmA by determining the crystal structure of the *O*-acetyltransferase CsaC and NMR measurements further reveal that the CsaC-mediated reaction is regioselective for O3 and that the O4 modification results from spontaneous *O*-acetyl migration.

## Introduction

*Neisseria meningitidis* (Nm) is an encapsulated, strictly human pathogen, which remains an important cause of bacterial meningitis and septicemia, especially in the African meningitis belt and in India^1^. The capsular polysaccharide (CPS) is the major virulence factor and serves as the basis for potent anti-meningococcal glycoconjugate vaccines^2^. Licensed formulations are available against serogroups A, C, W and Y, including an affordable monovalent anti-NmA vaccine developed for combatting the devastating meningococcal disease outbreaks in sub-Saharan Africa^3–5^. The NmA-CPS is joint by phosphodiester bridges^6,7^ that are highly susceptible to hydrolysis at elevated temperatures, which has major implications for the shelf-life and storage conditions of anti-NmA vaccines in tropical and subtropical areas^4,8^.

**Fig. 1:**
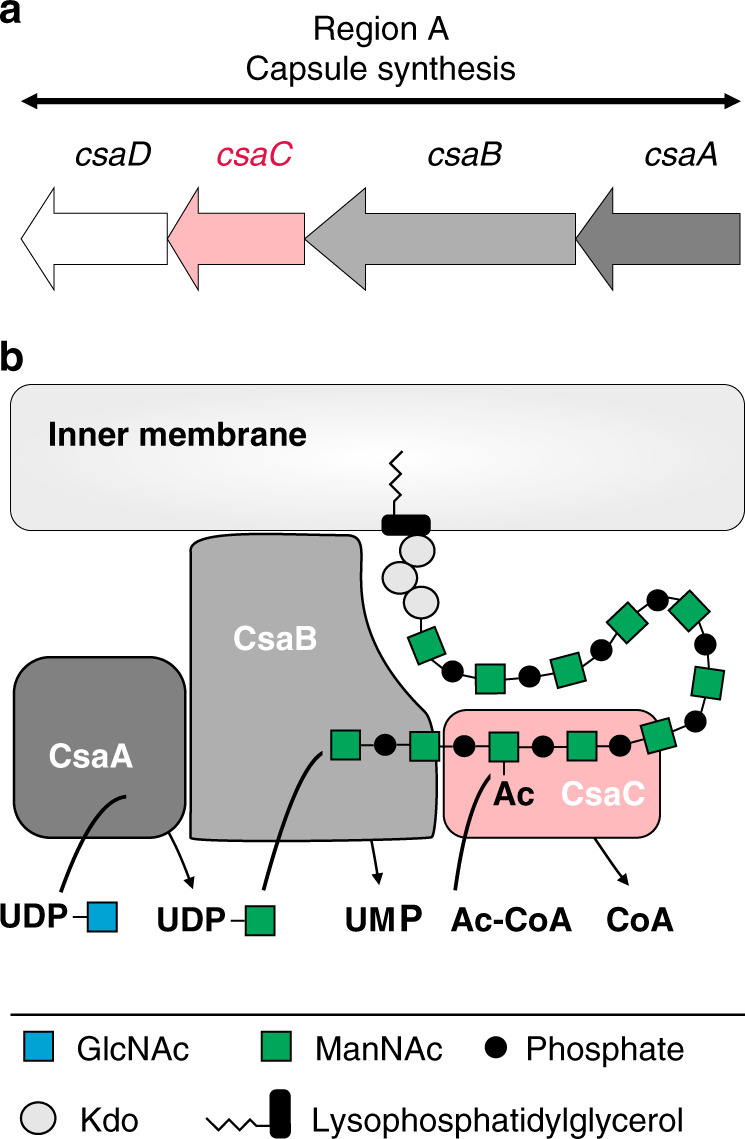
Capsule biosynthesis in *Neisseria meningitidis* serogroup A. **a** Schematic representation of region A of the capsule gene cluster of *Neisseria meningitidis* serogroup A^15^ encoding the UDP-*N*-acetyl-d-glucosamine-2-epimerase CsaA, the capsule polymerase CsaB, the capsule *O*-acetyltransferase CsaC, and a protein of unknown function (CsaD) predicted to be involved in CPS transport^15^. **b** Scheme summarizing the key steps of capsule biosynthesis in serogroup A. The UDP-*N*-acetyl-d-glucosamine-2-epimerase CsaA catalyzes the epimerization of UDP-GlcNAc to UDP-ManNAc. The capsule polymerase CsaB transfers ManNAc-1-phosphate units from UDP-ManNAc onto the non-reducing end of the growing polysaccharide chain [→6)-α-d-ManNAc-(1 → OPO_3_^−^ → ]. The *O*-acetyltransferase CsaC transfers acetyl-groups from acetyl-CoA onto the ManNAc units of assembled polysaccharide^14,16^. In all group 2 capsule biosynthesis complexes, the biosynthesis enzymes are believed to be membrane associated and the polymer is built upon a highly conserved acceptor consisting of lysophosphatidylglycerol and 3-deoxy-d-manno-oct-2-ulosonic acid (Kdo)^67^.

Composed of [→6)-α-d-ManNAc-(1 → OPO_3_^−^ → ] repeating units, the NmA-CPS backbone is heavily modified by variable degrees of *O*-acetylation at position C3 and C4^5,9–12^. Although four of the six clinically relevant Nm serogroups express *O*-acetylated capsules^7,12^, it is only the NmA-CPS, for which *O*-acetylation is mandatory to induce an effective immune response after vaccination^12,13^. Consequently, all vaccine formulations against NmA contain *O*-acetylated CPS and precise knowledge on the biosynthesis, intramolecular distribution, and functional impact of this modification is crucial for our understanding of meningococcal biology and tailored vaccine development. The key enzyme is the *O*-acetyltransferase CsaC (MynC) encoded in region A of the CPS gene cluster^14,15^. Gene deletion completely abolished capsule *O*-acetylation and biochemical studies identified the assembled polymer as acceptor substrate^14,16^, suggesting that *O*-acetylation occurs co- or post-synthetically (Fig. 1).

Aiming at a cost-effective synthesis platform that omits the cultivation of biohazardous material, we have recently established reaction conditions for the cell-free in vitro production of NmA-CPS. Usage of the recombinant biosynthetic enzymes CsaA, CsaB and CsaC (Fig. 1) allowed efficient in vitro assembly of the polymer and subsequent *O*-acetylation of 88% of the repeating units^16,17^. This approach, however, yielded exclusively 3-*O*-acetylated polymer and to date, the mechanism of CsaC-mediated *O*-acetylation and the origin of 4-*O*-acetylation are unknown. Moreover, structural information on meningococcal capsule *O*-acetyltransferases is, so far, only available for CssF (OatWY) of NmW and NmY. This enzyme belongs to the left-handed β-helix family of acyltransferases^18^ and shares no sequence similarity with CsaC.

Herein, we report three crystal structures of CsaC, in an apo and two substrate complexed forms, and provide mechanistic insight into NmA capsule *O*-acetylation. Supported by a comprehensive two-dimensional (2D) NMR characterization of the CPS, we demonstrate that the CsaC-mediated reaction is regioselective for O3 and that modification of O4 results from spontaneous *O*-acetyl migration. A comparative analysis of modified and unmodified NmA-CPS demonstrates that *O*-acetylation considerably stabilizes the polymer, allowing hydrolysis only to take place between non-*O*-acetylated sugar residues.

## Results

### 4-*O*-Acetylation of NmA CPS is due to acetyl migration

**Fig. 2:**
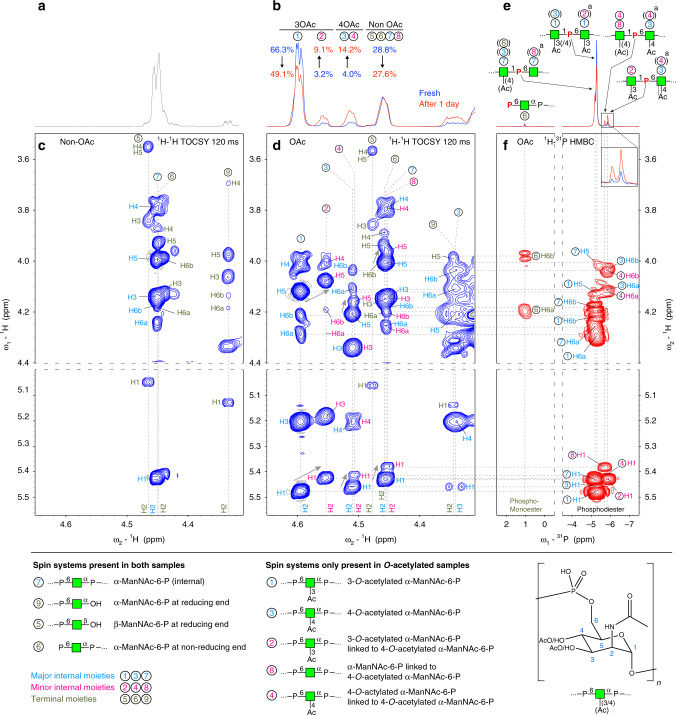
Comprehensive NMR analysis of *O*-acetylated and non-*O*-acetylated serogroup A polymer. **a**, **b** ^1^H NMR spectra of non-*O*-acetylated (**a**) and *O*-acetylated (**b**) serogroup A capsule polymer. **b** Freshly purified *O*-acetylated polymer (blue) was incubated at 45 °C and pH 7 for one day (red), leading to a shift in *O*-acetylation from C3 to C4. **c**, **d** 2D ^1^H-^1^H TOCSY (total correlation spectroscopy) spectra showing the H2 signal of each spin system on the ω2 axis and the correlations between H2 and the other ManNAc protons on the ω1 axis. Four spin systems were identified in the non-*O*-acetylated sample (**c**) and nine in the *O*-acetylated sample (**d**). The identified spin systems were labeled *1* to *9*, with the most downfield spin system labeled *1* and the most upfield labeled *9*. **e** ^31^P NMR of *O*-acetylated polymer. ^a^indicates spin systems that belong to moieties, which cannot be excluded as connecting residue. **f** ^1^H-^31^P HMBC spectrum corresponding to the 1D ^31^P spectrum shown in **e**.

For natural NmA-CPS contained in vaccines, total *O*-acetylation levels between 68–92% have been reported, of which roughly 10% is 4-*O*-acetylation^5,11,12^. To investigate, whether similar levels can be achieved by in vitro *O*-acetylation, we incubated CsaC with 1 mg of non-*O*-acetylated polymer in the presence of different donor (acetyl-CoA) to acceptor ratios, with each ManNAc moiety being considered as one acceptor site. Changing the ratio from 1:1 to 4:1 indeed increased the overall *O*-acetylation level from 70 to 92% (Supplementary Fig. 1a, b). Since acetyl groups can migrate between vicinal diols in a pH-dependent manner^7^, we next aimed at analyzing in vitro 3-*O*-acetylated NmA-CPS under different pH conditions to induce a potential 3,4-ester migration. Production of a larger batch of material for this experiment (20 mg) yielded 3-*O*-acetylated polymer, which already contained 4% of 4-*O*-acetylation (Fig. 2b, blue spectrum), indicating that acetyl migration occurred to a small extent already during the extended preparation time. Consequently, we aimed at reproducing these conditions in our experiment: The polymer was dissolved in phosphate buffer (pH 7.0) to mimic the enzymatic synthesis conditions, or in water (pH 5.6) to simulate the dialysis step of our processing protocol. The percentage of 3-*O*- and 4-*O*-acetylated moieties was determined by ^1^H NMR based on their characteristic H2 chemical shifts^7^. Already after one day, 4-*O*-acetylation was increased from 4 to 14% in phosphate buffer and remained constant upon prolonged incubation (Fig. 2b, red spectrum). In contrast, *O*-acetyl migration in water progressed considerably slower (Supplementary Fig. 2). Interestingly, the observed *O*-acetyl migration induced changes in the ^1^H and ^31^P NMR spectra (Fig. 2e, f; approximately −5.73 and −5.88 ppm) that were incongruent with previous assignments^14,19^. To address these discrepancies, we repeated the experiment and performed a comprehensive characterization of the *O*-acetylation pattern by 2D NMR (Fig. 2c–f).

### Two-dimensional (2D) NMR analysis of *O*-acetylated NmA-CPS

A complete de novo assignment was performed starting from the considerably less complex non-*O*-acetylated polymer (compare Fig. 2a, c with Fig. 2b, d). To increase signals resulting from the polysaccharide termini, we used partially hydrolyzed CPS, an approach that facilitated the assignment of the corresponding spin systems (Supplementary Fig. 3). A well-resolved part of a 2D ^1^H-^1^H TOCSY (total correlation spectroscopy) spectrum was chosen (Fig. 2c, d) that shows the H2 signal of each spin system on the ω_2_ axis and the correlations between H2 and the other ManNAc protons on the ω_1_ axis. A total of nine spin systems were identified (four in the non-*O*-acetylated and nine in the *O*-acetylated sample, with the most downfield and upfield systems in Fig. 2d labeled *1* and *9*, respectively (Supplementary Tables 1–3). Our data confirm the previous assignment of the major spin systems *1*, *3* and *7*, which belong to 3-*O*-acetylated, 4-*O*-acetylated and non-*O*-acetylated repeating units, respectively^7^.

The well-isolated, less abundant spin system *2* has been, so far, ambiguously assigned to either a 3,4-di-*O*-acetylated ManNAc^14^ or a 3OAc-ManNAc being connected to a 4OAc-ManNAc at its reducing end^7^. Spin system *2* shows correlations similar to those observed for *1* (3OAc-ManNAc), including the characteristic H3 chemical shift of 5.18 ppm, indicating *O*-acetylation at C3 (Fig. 2d and Supplementary Fig. 4). However, *1* and *2* differ by their H5 and H1 resonances (Fig. 2d, see arrows) and by the well-resolved ^31^P chemical shifts of the C1-linked phosphodiester (−5.28 versus −5.88 ppm, Fig. 2f). The latter finding suggests that *1* and *2* are quite similar, but connected to different neighboring units at their reducing ends. Following the vertical dotted lines of both ^31^P chemical shifts (−5.28 ppm for *1*, −5.88 ppm for *2*) and searching for correlations to the neighboring unit, reveals cross-peaks to characteristic H6a/H6b signals (Fig. 2f). The ^31^P resonance of the C1-linked phosphodiester of *2* correlates with H6a/H6b of *3* (4OAc-ManNAc), whereas the ^31^P resonance of the phosphodiester of *1* correlates to H6a/H6b of *1* (3OAc-ManNAc). Consequently, *1* belongs to a 3OAc-ManNAc that is linked at its reducing end to another 3OAc-ManNAc, whereas *2* refers to 3OAc-ManNAc linked to 4OAc-ManNAc. Owing to chemical shift degeneracies, a smaller population of *1* following nonOAc-ManNAc cannot be excluded. Importantly, the chemical shifts observed for H3/H4 (Fig. 2d) and C3/C4 (Supplementary Figs. 5 and 6) of *1* and *2* are almost identical, clearly excluding 4-*O*-acetylation of *2* and thus 3,4-di-*O*-acetylation on the same ManNAc moiety.

We observed two weak spin systems, namely *4* and *8*, that had not been described before. They show similar chemical shifts to the overlapping major spin systems *3* (4OAc-ManNAc) and *7* (nonOAc-ManNAc) (Fig. 2d), but differ by their H5 and H1 chemical shifts, reminiscent of the differences between *1* and *2*. The unique H1 chemical shift of *8* (5.381 ppm) shows a clear correlation to the unique ^31^P resonance at −5.72 ppm, which in turn correlates with H6a/H6b of *3* (4OAc-ManNAc, Fig. 2f). Thus, *8* is nonOAc-ManNAc linked to 4OAc-ManNAc at its reducing end. In accordance, *4* seems to be 4OAc-ManNAc linked to 4OAc-ManNAc at its reducing end. Unfortunately, the H1 resonance of *4* overlaps partially with other H1 resonances, preventing the identification of an unambiguous connection to the reducing end neighboring moiety. Also, the H2-H6a/H6b correlations of *4* are not clearly visible and likely hidden under the stronger H2-H6a/H6b correlations of *3*, making it difficult to deduce the connection to its non-reducing end moiety. However, since *3* and *4* belong to 4OAc-ManNAc moieties that differ only by the preceding neighbor, it is likely that the resonances of H6a/H6b are very similar. In summary, all signals of internal repeating units are present in a major (*1*, *3*, and *7*) and in a minor form (*2*, *4*, and *8*). The major forms represent units linked to 3OAc-ManNAc or nonOAc-ManNAc at their reducing ends, while the minor forms are linked to 4OAc-ManNAc. The quantification of the distinct structural motifs is presented as Supplementary Note 1.

Spin systems *5* and *9* were present in both samples (Fig. 2c, d) and were assigned to the β- and α-anomer of non-*O-*acetylated ManNAc at the reducing end, respectively, based on the similarity to published chemical shifts of free ManNAc^20^ (Supplementary Table 1). Spin system *6* originates from a terminal ManNAc with a phosphomonoester at C6 and represents the non-reducing end of the polymer as shown by a distinct ^31^P chemical shift of 0.63 ppm correlating to H6a/H6b of *6* in a ^1^H-^31^P HMBC (heteronuclear multiple bond correlation) spectrum (Fig. 2f and Supplementary Fig. 7).

Importantly, all termini identified in the *O*-acetylated sample resulted from nonOAc-ManNAc, indicating that hydrolysis of the *O*-acetylated polymer occurred exclusively between non-*O*-acetylated residues, suggesting their linkage to be less stable.

### *O*-Acetylation increases the stability of NmA-CPS

**Fig. 3:**
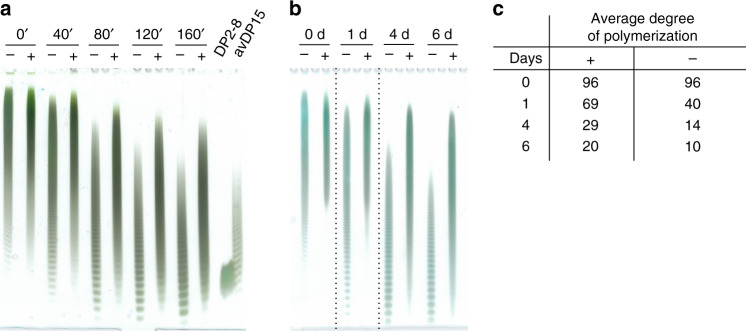
Stability of NmA-CPS. **a**, **b** Non-*O*-acetylated (−) and enzymatically *O*-acetylated (+) capsule polymer was subjected to mild acidic hydrolysis in acetate buffer pH 4.7 at 80 °C **a** and phosphate buffer pH 7.0 at 45 °C **b** to simulate the hydrolysis conditions during vaccine manufacturing and prolonged storage in tropical climate, respectively. Samples were taken at the indicated time-points and the experiment was continued until an average degree of polymerization (avDP) of 15 was reached, which reflects the avDP used for the generation of anti-NmA glycoconjugate vaccines^17,68^. Partially hydrolyzed polymers were separated by PAGE and visualized by Alcian blue/silver staining. An oligomer mix with avDP15 and a mixture of short oligomers containing dimers to octamers (DP2–8) were used as markers^17^. Source data are provided as a Source Data file. **c** The avDP of *O*-acetylated (+) and non-*O*-acetylated (−) polymer was monitored by ^31^P NMR under conditions corresponding to the experiment shown in **b**. According to a previously published method^19^, avDP values were calculated from ^31^P NMR signals and expressed as (*P*_Int_/*P*_Ter_) + 1, where *P*_Int_ is the molar concentration of the internal phosphate groups (phosphodiester groups) and *P*_Ter_ is the molar concentration of terminal phosphate groups (phosphomonoester groups).

Next, we analyzed the stability of *O*-acetylated and non-*O*-acetylated polymer under conditions that mimicked (i) hydrolysis during vaccine manufacturing (80 °C, pH 4.7, Fig. 3a) and (ii) storage of vaccines in tropical climate (45 °C, pH 7, Fig. 3b). Samples were taken at the indicated time-points and analyzed using Alcian blue/silver-stained polyacrylamide gel electrophoresis (PAGE). After both treatments, the *O*-acetylated polymer migrated slower than the non-*O*-acetylated polymer, indicating higher molecular mass and, therefore, higher stability. To confirm this finding, we quantified the change in the average degree of polymerization (DP) according to an established method^19^ that determines the average DP from the ratio between internal phosphodiesters and terminal phosphomonoesters obtained by ^31^P NMR (Fig. 2e). Again, OAc-CPS exhibited higher stability (Fig. 3c).

### Overall structure of CsaC

**Fig. 4:**
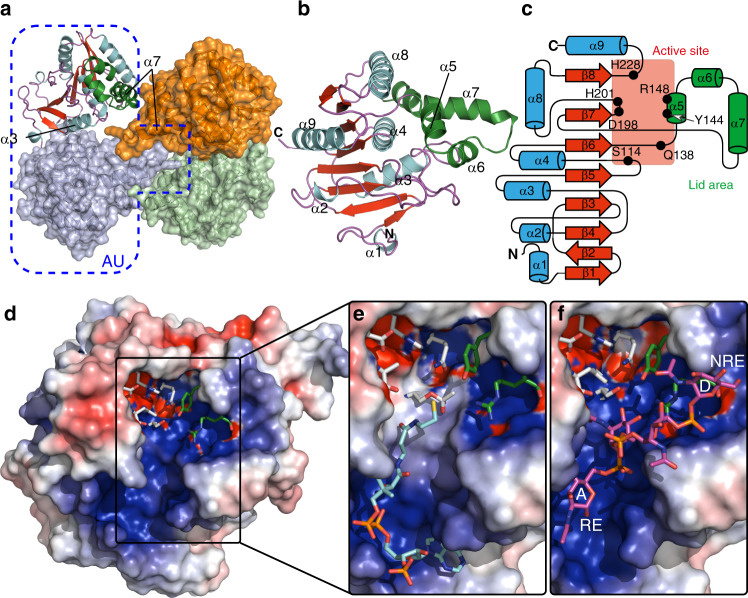
Crystal structure of CsaC. **a** Tetrameric assembly of CsaC with the contents of the asymmetric unit in blue outline. **b** Secondary structure arrangement of the CsaC protomer. **c** Secondary structure topology map of CsaC protomer with important active site residues marked. **d**–**f** Electrostatic surface potential (blue—positive, red—negative, white—neutral) with active site residues in stick representation of ligand-free wild type CsaC protomer (**d**), acetyl-CoA-soaked CsaC-H228A (**e**), and CPS-DP4-soaked wild type CsaC (**f**). Pyranose ring A at the reducing end (RE) and pyranose ring D at the non-reducing (NRE) end of the CPS-DP4 are highlighted.

**Fig. 5:**
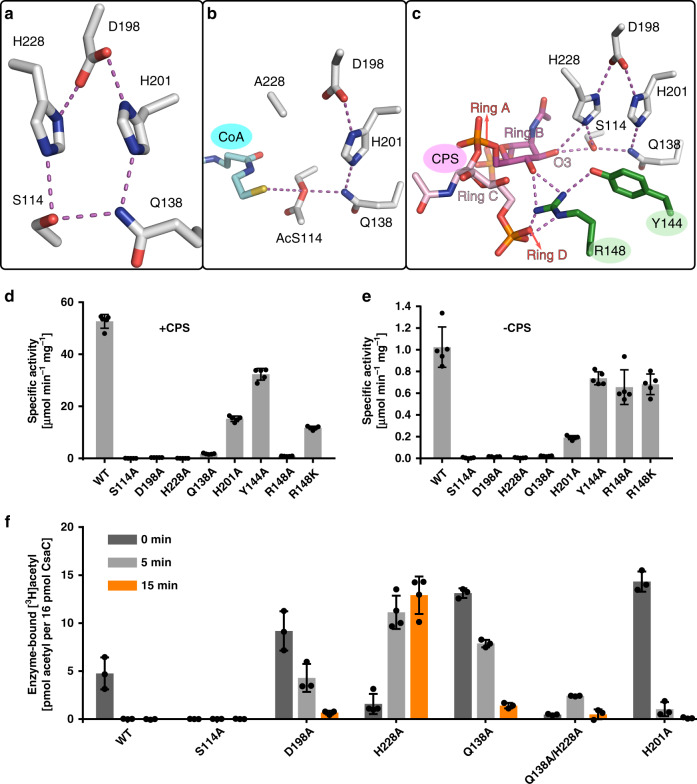
Active site description of CsaC. **a**–**c** Active site residues with hydrogen-bond network (distances ≤ 3.2 Å) for native wild type CsaC **a**, acetyl-CoA-soaked CsaC-H228A **b**, and CPS-DP4-soaked wild type CsaC **c**. **d** Transferase activity of wild type CsaC and active site mutants in presence of CPS (mean ± SD, *n* = 5 independent experiments). **e** Hydrolase activity towards acetyl-CoA measured in the absence of CPS (mean ± SD, n = 5 independent experiments). **f** Detection of acetyl-enzyme intermediates. Incorporation of radioactively labeled acetyl groups from [^3^H]acetyl-CoA was measured in the absence of CPS (mean ± SD, *n* = 4 independent experiments for CsaC-H228A and *n* = 3 independent experiments for CsaC-WT and all other variants). Source data are provided as a Source Data file.

To provide insight into the structural and mechanistic basis of CPS *O*-acetylation, we solved the structure of wild type CsaC (CsaC-WT, 2.0 Å resolution) in its apo state as well as complexes of CsaC-H228A with acetyl-CoA (2.0 Å resolution) and CsaC-WT with a tetrasaccharide fragment of the natural acceptor substrate (CPS-DP4, 1.95 Å resolution) (Supplementary Table 4). Consistent with the oligomeric state of CsaC in solution (Supplementary Fig. 8), the crystal packing of all CsaC structures contains a tetrameric assembly with two protein molecules in the asymmetric unit (Fig. 4a). Each protomer consists of a central eight-stranded β-sheet that is sandwiched by six α-helices and capped by a lid area formed by helices α5, α6, and α7 (Fig. 4b, c). Contact between the protomers is mainly mediated by helices α3 and α7 (Fig. 4a and Supplementary Fig. 9). A deep positively charged groove, which traverses the protein surface (Fig. 4e, f), allows substrate binding and opens into an active site that is characterized by a catalytic triad composed of S114, H228 and D198 (Fig. 5a). A DALI search^21^ revealed structural homology of CsaC with serine ester hydrolases of the α/β-hydrolase (ABH) fold superfamily, a large protein family that encompasses mainly hydrolytic enzymes^22–24^, as exemplified with esterase A from *Streptococcus pyogenes* (Supplementary Fig. 10, *Z*-score 18.4).

### CoA binding in the acetylated H228A(-AcS114)-CoA complex

As soaking of CsaC-WT crystals with acetyl-CoA or CoA yielded only incomplete substrate density and co-crystallization trials were unsuccessful, we soaked crystals of the catalytically impaired variant H228A with acetyl-CoA. All protomers showed complete density for CoA with a free SH-group (Fig. 4e and Supplementary Fig. 11a). Concomitantly, the triad serine showed an extended electron density with a contour indicative of its *O*-acetylated form (AcS114) (Fig. 5b and Supplementary Fig. 11a). This suggests that CsaC adopted the double displacement mechanism of ABH esterases^24^ and forms a transient acetyl-enzyme intermediate that is trapped in the absence of H228 (Supplementary Fig. 12). CoA, as the first reaction product, is bound along the positively charged groove in an L-shaped conformation (Fig. 4e). The pantetheine moiety is engaged in hydrophobic contacts (I233, A39, and F40), and only the distal carbonyl oxygen is specifically recognized by S113-Oγ, a contact that directs the SH-group towards the triad serine S114 (Supplementary Fig. 13). The purine nucleobase of CoA is sandwiched between I11 and K48 and locked into position by a hydrogen bond between adenine-N7 and Y49-N, while the phosphate oxygens of the ADP-3′-phosphate moiety are encased by a hydrogen network provided by R237, K53, Y51 and I52 (Fig. 4e and Supplementary Fig. 13).

### The acceptor binding site accommodates four ManNAc moieties

To investigate the structural basis of acceptor binding, crystals of CsaC-WT were soaked with a CPS fragment consisting of four ManNAc moieties (CPS-DP4), which is sufficiently long to serve as acceptor substrate (Supplementary Fig. 14). For the complete tetrasaccharide, the electron density is found in the positively charged groove (Fig. 4f and Supplementary Figs. 11b and 15). An assessment of the substrate electron densities of DP4, DP5, and DP6 soaked crystal structures revealed no significant difference in dimension and location. Since data statistics and quality of the electron density map were best for the DP4 data set, further analysis was performed on these data. A high-affinity acceptor binding site can be identified that accommodates four ManNAc moieties, designated A to D from reducing to non-reducing end. While the tetrasaccharide traverses the active site with the non-reducing end pointing towards the lid domain, the internal ManNAc moiety B faces the reactive center. The binding site of ManNAc moiety A and B overlaps with that of the pantetheine arm of CoA (Fig. 4e, f and Supplementary Fig. 15), indicating that CoA is released prior to acceptor binding. Contacts between acceptor and CsaC are mainly facilitated by residues K43, S113, S114, R148, and H228 (the latter three are illustrated in Fig. 5c and Supplementary Fig. 16).

### CsaC shows an unconventional active site architecture

CsaC harbors a serine-histidine-aspartate triad composed of S114, H228, and D198, which occur in the topological arrangement of the canonical ABH-fold, with S114 being part of a nucleophilic elbow motif (GX^114^SXG), a typical feature of ABH-enzymes that places the nucleophile at the tip of a sharp turn^22,24^. In CsaC, however, the classical triad is amended by two additional residues, H201 and Q138, yielding a bifurcated network of hydrogen-bond donors and acceptors with D198 as bifurcation point and the amino acid pairs H228/S114 and H201/Q138 as the two arms (Fig. 5a). Beyond the catalytic center, the H201/Q138 arm is extended by Y79 (Supplementary Fig. 17), which allows precise positioning of Q138 by engaging its Nε2 and Oε1 via hydrogen-bonding with the flanking H201-Nε2 and Y79-OH, respectively. In this unusual active site arrangement, S114-Oγ is in hydrogen-bond distance not only to H228-Nε2 as in a classical triad, but also to Nε2 of Q138, which appears to be a unique element of CsaC. In the H228A(-AcS114)-CoA complex, Q138 positions AcS114, which is approached on the opposite side by the SH-group of CoA (Fig. 5b). In the CsaC-CPS complex, the acceptor O3 of ring B is in H-bond distance to S114-Oγ and H228-Nε2 (Fig. 5c). The latter is, thus, well-positioned to act as general base deprotonating O3. In contrast, and similar to the situation in the H228A(-AcS114)-CoA complex, Q138 is located opposite to the substrate entry site and, thus, lacks direct substrate interactions (Fig. 5c). The catalytic unit of CsaC is accomplished by an oxyanion hole provided by the backbone amides of F40 and K115 (Supplementary Fig. 18), suggesting that the CsaC-catalyzed transfer mechanism involves oxyanion-containing tetrahedral intermediates as proposed in Supplementary Fig. 12.

### Enzymatic activity of WT and mutant forms of CsaC

CsaC-WT shows high transfer activity towards CPS and a 52-fold lower transfer activity towards water (measured as enzyme-dependent hydrolysis of acetyl-CoA in the absence of CPS) (Fig. 5d, e). As expected, single alanine replacement of the triad residues S114 and H228 resulted in a complete abrogation of detectable transfer activity, and a D198A exchange reduced transfer activity by 99.5% (+CPS) and 98.6% (−CPS). However, a severe drop in activity by 96.8% (+CPS) and 98.0% (−CPS) was also seen for Q138A, revealing a substantial contribution of this unconventional active site residue to catalysis. Exchange of H201, involved in the positioning of Q138, reduced transfer activity by over 70%. An R148A replacement extinguished transfer activity towards CPS, but not towards water, substantiating a critical role of R148 in CPS placing. The loss of activity towards CPS is only partially rescued by an R148K exchange (Fig. 5d) as only the guanidinium function allows the dual H-bonding with two acceptor sites, as seen in the CsaC-CPS complex (Fig. 5c and Supplementary Fig. 16). Replacement of Y144, which is H-bonded with R148, abolished transfer activity by 38.6% (+CPS) and 28.0% (−CPS).

To monitor the formation of the transient acetyl-enzyme intermediate, we incubated CsaC-WT in the presence of [^3^H]acetyl-CoA. Radiolabeled protein was detected directly after mixing and quickly disappeared thereafter due to hydrolysis of the acetyl-enzyme adduct and complete turnover of acetyl-CoA (Fig. 5f). Covalent adduct formation with S114 was confirmed by liquid chromatography-electrospray ionization-tandem mass spectrometry, while with both methods no adduct was seen for CsaC-S114A (Fig. 5f and Supplementary Figs. 19–22). CsaC-D198A, −Q138A and −H201A rapidly formed a covalent intermediate, but showed decelerated adduct hydrolysis compared to CsaC-WT (Fig. 5f). CsaC-H228A, in contrast, showed no detectable adduct hydrolysis, demonstrating an essential function of H228 in the second half-reaction, i.e., transfer of the acetyl group from AcS114 to the acceptor (CPS or water). However, how is the acetyl adduct formed by CsaC-H228A in the first half-reaction, in which the triad histidine usually enables the adduct formation by increasing the nucleophilicity of the catalytic serine? In CsaC, the only other residue that could fulfill this function is Q138, assuming the basicity of its amide function was increased, e.g., by amide-imidic acid tautomerism as proposed in Supplementary Fig. 12b. A similar imine-mediated pathway for proton elimination is discussed for ζ-fold prenyltransferases^25^ and the imidic form of an amide side chain as part of a proton transfer network has been directly shown for endoglucanase by neutron crystallography^26^. In support of our hypothesis that Q138 can substitute H228 as a general base in the first half-reaction, Q138 is optimally positioned in both CsaC-WT and the CsaC-H228A(-AcS114)-CoA complex (Supplementary Fig. 23) and impaired acetyl-adduct formation was seen upon simultaneous replacement of H228 and Q138 (Fig. 5f). In the second half-reaction, Q138 is too distant from the CPS acceptor to substitute H228. Yet, the transfer activity of CsaC-Q138A is severely impaired, suggesting a critical role of Q138 in optimal positioning of AcS114 for the incoming nucleophile and stabilization of a subsequently formed tetrahedral intermediate (Supplementary Figs. 12a and 18b, c).

### Structural basis of regioselective *O*-acetylation of CPS

**Fig. 6:**
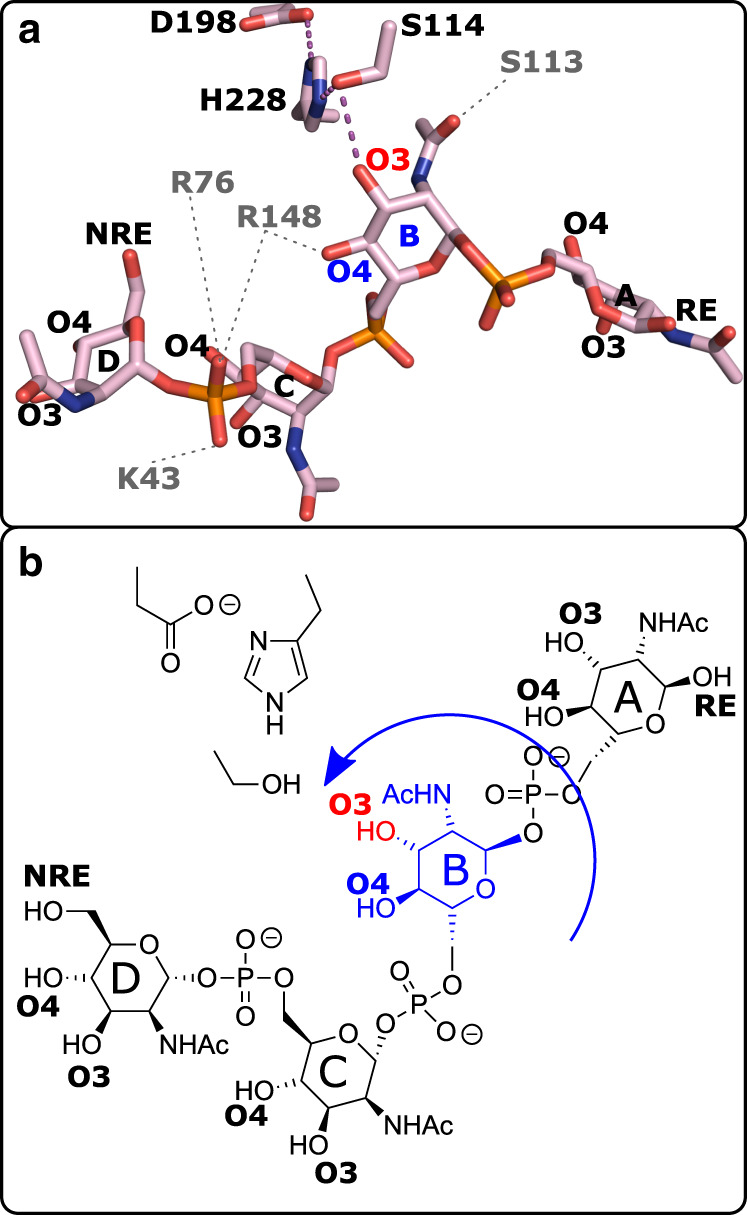
Orientation of CPS-DP4 rings A–D. Crystal structure of wild-type CsaC soaked with CPS-DP4 (**a**) and schematic representation (**b**). O3 (red) of ring B (blue) is in physical proximity to the catalytic triad serine 114, while O4 protrudes away from the active site. Hydrogen bond (dashed magenta lines) distances are ≤3.2 Å. Gray dashed lines indicate coordination by amino acid residues depicted in gray single-letter code. Amino acid residues of the catalytic triad are labeled in black.

Analysis of the CsaC-CPS complex revealed that the bound tetrasaccharide adopts a step-like conformation with the central phosphodiester bridge dividing the molecule into a reducing disaccharide and a non-reducing disaccharide half (Fig. 6). Specific interactions with CsaC are limited to pyranose ring B and the phosphodiester bridge interconnecting ring C and D. The orientation of ring B within the substrate entry site allows precise positioning of O3 for nucleophilic attack (Figs. 5c and 6). Selective access of the 3-OH to the reactive center is ensured by involving the flanking functional groups in acceptor-enzyme interactions. While the carbonyl oxygen of the C2 *N*-acetyl group is in hydrogen bonding with the hydroxyl group of S113, the C4 hydroxyl group is engaged in a bifurcated H-bond with R148 (Figs. 5c and 6; and Supplementary Fig. 16). Anchoring of the guanidinium group of R148 by two flanking H-bonds (with Y144 and the phosphodiester between ring C and D) directs the central R148-O4 interaction away from the catalytic center, thus ensuring regioselective attack of O3.

## Discussion

Native NmA-CPS on intact cells as well as purified polymer contain *O*-acetyl groups in position O3 and O4 of the ManNAc repeating units^27^. As genetic deletion of the *O*-acetyltransferase CsaC resulted in a complete loss of CPS *O*-acetylation^14^, it was previously concluded that CsaC has dual specificity for O3 and O4 and, thus, allows 3-*O*-, 4-*O*- and even 3,4-di-*O*-acetylation of the ManNAc units^14,27^. However, our data demonstrate strict regioselectivity of CsaC for O3 and uncovered 4-*O*-acetylation as a secondary effect due to acetyl migration between cis-diols, which may proceed via a cyclic orthoester derivative^28^. The non-enzymatic shift from O3 to O4 occurred upon the storage of 3-*O*-acetylated CPS and yielded an *O*-acetylation pattern (56.8% O3 and 13.9% O4) comparable to the natural polymer^5,7^. The phenomenon of *O*-acetyl group migration is well known in the context of sialic acids^7,29,30^. Consistent with our findings, *O*-acetyl group migration observed in the polysialic acid CPS of NmC occurred over a period of days when monitored in water^7^ and migration rates of free sialic acids increased at pH values above 6, with high rates and partial *O*-acetyl group losses at pH values above 7.5^29,30^. Considering that CPS harvest and purification is performed under neutral to mild basic conditions, the varying levels of 3-*O*- and 4-*O*-acetylation that have been reported for NmA-CPS^5,7,27^ might mainly reflect variations in the CPS processing protocols.

Our comprehensive 2D NMR analysis highlighted the importance of multiple correlation spectra to assign the complex NMR signals of the NmA-CPS, which were attributed to nine different structural motifs. The NMR data exclude the presence of 3,4-di-*O*-acetylated ManNAc units and helped to overcome ambiguities that led to misassignments in the past^14^. The newly acquired knowledge allows precise quantification of the *O*-acetylation level on the basis of eight spin systems (Supplementary Note 1) and contributes to improved quality control of vaccine formulations^31^.

Owing to the chemical lability of the anomeric phosphodiester linkage in aqueous solution, the NmA-CPS is the least stable of the meningococcal polysaccharides^4,19^. Efforts to generate stable glycomimetics yielded, so far, only compounds that are less immunogenic than the natural epitope^32–34^. However, approaches to improve their antigenic properties are ongoing^35–38^, and in this context site-selective, enzymatic *O*-acetylation might provide a valuable tool.

Our results show that *O*-acetylation significantly increases the stability of the NmA CPS and that hydrolysis of anomeric phosphodiester linkages occurs exclusively between non-*O*-acetylated ManNAc units. Comparison with the more stable NmX-CPS ([ → 4)GlcNAc-(1-P → ]n), which contains GlcNAc instead of ManNAc and lacks *O*-acetylation, suggests that the axial orientation of the C2 *N*-acetyl group of ManNAc favors carbonyl-assisted intramolecular hydrolysis of the anomeric phosphodiester linkage in the NmA-CPS^19^. The introduction of an electron-withdrawing *O*-acetyl group in C3 of ManNAc will mitigate the attack of the carbonyl oxygen in C2, which might explain the increased stability of *O*-acetylated NmA-CPS. Moreover, molecular dynamics simulations predicted that *O*-acetylation shifts the conformational preference of NmA-CPS from a compact to an extended form^36^. Thus, *O*-acetylation may not only increase CPS stability, but also define the polysaccharide dimensions and, thus, the thickness of the capsule. In summary, and together with our finding that CsaC *O*-acetylates also shorter oligosaccharides, our data offer new possibilities for using CsaC as tool for optimized, site-selective *O*-acetylation (i) to increase the stability of size-reduced NmA-CPS used for glycoconjugate vaccines, which are currently produced under harsh conditions that may cause a partial loss of *O*-acetyl groups (*O*-acetylation levels can be as low as 61.5%^39^) and (ii) to increase the immunogenicity of non-native glycomimetics, which show improved stability, but are less immunogenic than the native NmA-CPS^32^.

Our structural analysis of CsaC identified the capsule *O*-acetyltransferase of NmA as an α/β-fold enzyme with Ser-His-Asp triad. The Ser-His-Asp triad occurs in several distinct folds, including the ABH-fold, subtilisin fold, chymotrypsin fold and SGNH-fold, and is mainly seen as core catalytic machinery in hydrolytic enzyme reactions^40,41^. So far, several bacterial cell wall *O*-acetyltransferases with a Ser-His-Asp triad have been crystallized and all present an SGNH- or SGNH-like fold^42–45^. The crystal structure of CsaC revealed that this polysaccharide *O*-acetyltransferase adopts an ABH-fold and, thus, clearly differs from the above mentioned transferases (see Supplementary Figs. 24 and 25 for comparison). The large ABH-fold superfamily contains primarily hydrolases, but also encompasses members with other catalytic functions^22,46^. Based on a central ABH-fold as scaffold for a catalytic triad, functional variability can arise from additional structural elements. Insertions in the C-terminal part frequently form lid structures that restrict access to the catalytic center and/or shape the substrate-binding pocket^46^. In CsaC, the lid area provides R148, the pivotal residue in acceptor positioning, which critically contributes to acceptor-substrate binding and regioselectivity.

The identification of an acetyl-serine adduct indicates that CsaC follows the canonical mechanism of ABH-esterases, a double-displacement bi-bi mechanism involving two tetrahedral oxyanion intermediates^22^. Different from esterases, however, the CsaC-mediated reaction involves a sugar hydroxyl group instead of water for cleavage of the acetyl-enzyme intermediate during the second half-reaction (Supplementary Fig. 12a). So far, structural information on ABH-transferases is largely limited to enzymes that are listed in the ESTHER database^47^ as members of the homoserine transacetylase (HAT) subfamily. Like CsaC, HATs transfer acetyl groups from acetyl-CoA to a hydroxyl group, but use l-homoserine, l-serine, or deacetylcephalosporin C as acceptor substrates^48–50^. Despite sharing a Ser-His-Asp triad and catalyzing similar reactions, HATs and CsaC show closer structural homology to different subsets of ABH-esterases than to each other and differ in the fine structure of their active sites. HATs not only lack the His201-Q138-Y79-based triad extension seen in CsaC, but also show a different main-chain conformation of the oxyanion loop. In HATs, this conformation is distinct from esterases to lower the activity of water in the active site^22^. CsaC, by contrast, shows an “activating” esterase-like conformation (Supplementary Fig. 26) and substantial hydrolysis of the acetyl-enzyme adduct was observed in the absence of CPS. Thus, in the acceptor-bound state, the optimal geometry of the enzyme-substrate complex might be key to favor the acceptor hydroxyl over water. This affords precise positioning of both acceptor hydroxyl and acetyl-serine adduct, which is mediated by R148 and Q138, respectively, the two non-triad residues that are critical for CsaC-mediated CPS *O*-acetylation. Notably, Q138 is highly conserved in several uncharacterized bacterial proteins with a nucleophile elbow motif (Supplementary Fig. 27 and Supplementary Data 1), suggesting that the unusual active site arrangement of CsaC is shared by a panel of putative ABH-fold enzymes of unknown function. Future analyses of these enzymes may show, whether extension of the Ser-His-Asp triad by an extra glutamine evolved as a general strategy to transform ABH-fold esterases into transferases.

## Methods

### Cloning and purification

The generation of the plasmid pStrep-CsaC-His was described previously^16^. For the generation of pCsaC-His, *csac* was amplified using the primers AB90/FF57 (Supplementary Table 5) and pStrep-CsaC-His as template. The resulting PCR product was cloned back into pStrep-CsaC-His via *Nde*I/*Xho*I, leading to the loss of the Strep tag. All mutations were introduced using the primers shown in Supplementary Table 5 and the QuikChange^®^ XL Site-Directed Mutagenesis Kit (Stratagene) according to the manufacturer’s guidelines. Constructs were expressed in *Escherichia coli* BL21(DE3) and purified on an ÄKTA FPLC (GE Healthcare) via their C-terminal His-tag using immobilized nickel affinity chromatography with 50 mM Tris pH 8.0, 300 mM NaCl, 20 mM imidazole as binding buffer and 50 mM Tris pH 8.0, 300 mM NaCl, 500 mM imidazole as elution buffer. Protein containing fractions were pooled, transferred into storage buffer (50 mM Hepes pH 7.0, 100 mM NaCl, 5 mM MgCl_2_, 1 mM EDTA) and snap-frozen in liquid nitrogen^16^.

### CsaC-mediated in vitro *O*-acetylation

CsaC-mediated *O*-acetylation of either 1 or 20 mg of in vitro synthesized non-*O*-acetylated polymer was performed for 4 h at 37 °C in the presence of 0.8 µM CsaC and the indicated concentration of acetyl-CoA (sodium salt, GERBU) (Supplementary Fig. 1) in a total volume of 0.5 mL (1 mg) or 10 mL (20 mg) of acetylation buffer (25 mM Tris, pH 7.5, 50 mM NaCl). The modified polymer was purified by anion exchange chromatography (AEC) using a MonoQ HR 5/5 column or a MonoQ 10/100 GL column (both GE Healthcare) and a linear sodium chloride gradient. CPS containing fractions eluting at 500–600 mM NaCl were pooled, dialyzed against water (ZelluTrans, Roth, 1 kDa molecular mass cutoff) and freeze-dried for NMR analysis^16^.

### Nuclear magnetic resonance spectroscopy

All spectra were recorded on a 600 MHz Avance III HD spectrometer (Bruker Biospin, Germany) equipped with a ^1^H/^13^C/^15^N/^31^P-QXI probe at 298 K. Typically, lyophilized samples were dissolved in 500 μL D_2_O (100% D, Armar, Leipzig, Germany) and transferred to standard 5 mm tubes (type TA, Armar, Leipzig, Germany). Concentrations ranged from 1 mg/mL to 5 mg/mL. One-dimensional ^1^H spectra were recorded with 64 scans, recycle delays of 2 s, 65536 points and a spectral width of 20 ppm resulting in a measurement time of 5 min. One-dimensional ^31^P spectra were recorded using 352 scans, a recycle delay of 3 s, 16384 points and a spectral width of 50.1 ppm resulting in a measurement time of 22 min. Two-dimensional (2D) ^1^H-^1^H COSY spectra were acquired with the pulse sequence cosygpppf (Bruker library) using 32 scans, a recycle delay of 2 s, 2048 × 128 points and spectral widths of 8.33 × 8.33 ppm with a duration of ~2 h. Standard 2D ^1^H-^1^H TOCSY spectra were measured using a mixing time of 80 ms, 4 scans, a recycle delay of 2 s, 2048 × 512 points and spectral widths of 8.33 × 8.33 ppm with a duration of 1 h 30 min. Two-dimensional (2D) ^1^H-^13^C HSQC spectra were recorded using hsqcedetgpsisp2.2 (Bruker library) with 32 scans, a recycle delay of 1.5 s, 2048 × 230 points and spectral widths of 16.0 × 100.4 ppm with a duration of 3 h 30 min. Two-dimensional (2D) ^1^H-^13^C HMBC spectra were recorded using the pulse sequence hmbcgpndqf (Bruker library) with 64 scans, a recycle delay of 2 s, 4096 × 512 points and spectral widths of 20.02 × 222.4 ppm with a duration of 20 h 30 min. Two-dimensional (2D) ^1^H-^31^P HMBC spectra were acquired using the pulse sequence hmbclpndqf (Bruker library) with 32 scans, a recycle delay of 1.5 s, 4096 × 64 points and spectral widths of 10.0 × 30.5 ppm with a duration of 1 h 8 min unless stated otherwise. Spectra were processed with Topspin 3.6.1 (Bruker Biospin, Germany) and analyzed with Sparky 3.115 (Goddard T.D., Kneller D.G. (2008) SPARKY 3. University of California, San Francisco, CA). The ^1^H resonances were referenced to DSS (4,4-dimethyl-4-silapentane-1-sulfonic acid) using an external sample of 2 mM sucrose containing 0.5 mM DSS (standard sample for water suppression from Bruker Biospin, Germany). ^13^C, ^15^N, and ^31^P axis were referenced indirectly according to IUPAC-IUBMB-IUPAB^51^ using the chemical shift referencing ratios of 0.251449530, 0.101329118, and 0.404808636.

### Generation of CPS-derived oligosaccharides with defined DP

Serogroup A oligosaccharides of defined DP were produced by partial hydrolysis of the phosphodiester bond, starting from enzymatically synthesized, non-*O*-acetylated serogroup A polymer^17^. The polymer was incubated in sodium acetate buffer (50 mM sodium acetate, pH 4.8) for 6 h at 73 °C, yielding oligosaccharides that carry a phosphomonoester at the non-reducing end^19,52^, which could be removed by calf intestinal alkaline phosphatase^16,53^. Obtained oligosaccharides were purified by AEC on an ÄKTA FPLC system (GE Healthcare) equipped with a MonoQ HR 5/5 column (GE Healthcare). H_2_O and 1 M NaCl were used as mobile phases M1 and M2, respectively, at a flow rate of 1 mL/min. Samples were separated by consecutive linear gradient steps (0–5% M2 over 1 mL, 5–20% M2 over 10 mL, and 20–30% M2 over 20 mL). Acetate and GlcNAc-1P were used to calibrate the column. The amount of oligosaccharide in each fraction was estimated from the peak area obtained at 214 nm under the assumption that each ManNAc residue contributes equally to the absorbance of the respective oligomer. Fractions were dialyzed against water (ZelluTrans, Roth, 1-kDa molecular mass cutoff) and freeze-dried, and equal concentrations were adjusted with water. Identity and DP of the size-fractionated oligosaccharides were confirmed by HPLC-AEC and HPAEC-PAD (high-performance anion exchange chromatography with pulsed amperometric detection). HPLC-AEC was performed on a Prominence UFLC-XR system (Shimadzu) equipped with a CarboPac PA-100 column (Dionex, Thermo Fisher Scientific). Samples were separated at 50 °C with H_2_O and 1 M NaCl as mobile phases M1 and M2, using a −2 curved gradient from 0 to 30% M2 over 4 min followed by a linear gradient from 30 to 84% M2 over 33 min^54^ (Supplementary Fig. 14a). LCsolution version 1.25 SP4 (Shimadzu) was used for data collection and analysis. HPAEC-PAD^19^ was performed on an ICS 5000 system (Dionex, Thermo Fisher Scientific) equipped with a CarboPac PA200 column (Thermo Fisher Scientific) calibrated with ManNAc and ManNAc-6P (Carbosynth) (Supplementary Fig. 14b). Samples were separated at 30 °C with H_2_O, 1 M NaNO_3_ and 500 mM NaOH as mobile phases M1, M2 and M3, using a constant concentration of 20% of M3 and a linear gradient from 1 to 16% of M2 over 50 min. Chromeleon 7.2 SR4 (Thermo Scientific) was used for data collection and analysis.

### CPS stability assay

To analyze the stability of *O*-acetylated and non-*O*-acetylated NmA-CPS, 3.9 mg of CPS were dissolved in 105 µL of 50 mM sodium acetat buffer pH 4.7 and incubated at 80 °C. After the indicated time points (Fig. 3a), 15 µL of sample were neutralized at room temperature with 0.4–0.6 µL of 1 M NaOH and supplemented with 15 µL of 2 M sucrose. Oligomers were separated by high percentage (25%) PAGE performed at 4 °C and 400 V and stained with 0.5% Alcian blue and 0.6% silver nitrate^16,17,55^. Samples shown in Fig. 3b were prepared as described below for Fig. 3c and analyzed as described above for Fig. 3a. For ^31^P NMR analysis (Figs. 3c), 4.8 mg of NmA-CPS were dissolved in 525 µL of 57 mM potassium phosphate buffer pH 7 and incubated at 45 °C. The pH was monitored before and after the experiment (pH 6.96 for non-OAc- and pH 6.80 for OAc-CPS). The avDP values were calculated from ^31^P NMR signals of internal phosphate groups (phosphodiester groups) and terminal phosphate groups (phosphomonoester groups)^19^.

### Analysis of acetyl group migration

*O*-Acetylated NmA-CPS (1.6 mg) was dissolved in 550 µL D_2_O (final pH of 5.6) or 550 µL potassium phosphate buffer (final pH of 7.0) and incubated for the indicated time period (Fig. 2b and Supplementary Fig. 2).

### *O*-Acetyltransferase assay

Enzymatic activity of purified CsaC variants was determined in a spectrophotometric assay with 5,5-dithio-bis-(2-nitrobenzoic acid) (DTNB). The reaction was performed at 30 °C in a total volume of 100 µL containing 50 mM Tris-HCl pH 7.5, 50 mM NaCl, 20% glycerol, 2 mM DTNB (Sigma) and 1 mM acetyl-CoA (sodium salt, GERBU) with or without 160 µg/mL non-*O*-acetylated NmA-CPS. The reaction was initiated by adding 16 pmol of purified enzyme. A reaction without enzyme was performed as blank to account for spontaneous hydrolysis of acetyl-CoA. The generation of CoA was monitored by reaction of its free sulfhydryl group with DTNB, which yields a mixed disulfide and 5-thionitrobenzoic acid. The latter was measured continuously at 405 nm in half-area 96-well plates (Greiner) using a PowerWave 340 microtiter plate spectrophotometer (BioTek). The change in optical density at 405 nm was related to a standard curve obtained for free CoA (Sigma).

### Radioactive incorporation assay

To monitor the formation of acetyl-enzyme adducts, 2.5 µg of purified CsaC variants were incubated at 37 °C with 5 µM (740 Bq/pmol) [^3^H]acetyl-CoA (American Radiolabeled Chemicals) in 25 µL reaction buffer (50 mM Tris-HCl pH 7.5, 50 mM NaCl). The reaction was stopped after 0, 5 and 15 min by spotting 5 µL of the reaction mixture (containing 16 pmol CsaC) on Whatman 3MM paper. Free radioactivity was removed by descending paper chromatography in 70% ethanol containing 300 mM ammonium acetate buffer pH 7.5. Radioactivity that was incorporated into the chromatographically immobile protein fraction was quantified by scintillation counting.

### Analysis of acetyl-enzyme adducts by liquid chromatography–electrospray ionization–mass spectrometry (LC–ESI–MS)

For detection of acetyl enzyme adducts by mass spectrometry, 2 µg of the enzyme (64 pmol) were mixed with 5 µM acetyl-CoA in a final volume of 10 µL of reaction buffer (50 mM Tris-HCl pH 7.5, 50 mM NaCl). The reaction was immediately stopped by adding Laemmli sample buffer, and the reaction mixture was separated by sodium dodecyl sulfate–polyacrylamide gel electrophoresis. Coomassie Blue-stained protein bands were excised and digested with trypsin and Asp-N. Briefly, the gel pieces were dehydrated with acetonitrile and rehydrated with 100 mM NH_4_HCO_3_ containing 10 mM dithiothreitol (Roth). Subsequently, gel pieces were treated with 100 mM NH_4_HCO_3_ containing 100 mM iodoacetamide (Sigma). After a second dehydration step with acetonitrile and rehydration with 100 mM NH_4_HCO_3_ buffer followed by dehydration with acetonitrile, the dried gel pieces were rehydrated with 20 ng/μL trypsin (Promega) in 50 mM NH_4_HCO_3_ buffer and incubated overnight at 37 °C. Peptides were extracted with 75% acetonitrile containing 0.1% formic acid and dried in a vacuum centrifuge. Peptides were dissolved in 4 ng/µL Asp-N (Promega) in 10 mM Tris-HCl buffer (pH 8.0), incubated overnight at 37 °C and dried again. MS analysis of peptides was performed on a nanoACQUITY UPLC system (Waters) equipped with an analytical column (Waters, BEH130C18, 100 μm × 100 mm, 1.7 μm particle size) coupled online to an ESI Q TOF (Q TOF Ultima, Waters). Peptides were dissolved in 2% acetonitrile, 0.1% formic acid and separated by reverse phase chromatography using acetonitrile as eluent. MS spectra were recorded in positive reflection mode and analytes were automatically subjected to fragmentation (MS/MS). Spectra were analyzed using MassLynx V4.1 software (Waters). MS/MS protein spectra were automatically analyzed using the program ProteinLynx Global Server (Version 2.1, Waters).

### CsaC crystallization and structure determination

Native wild type CsaC and CsaC-H228A were crystallized in sitting drop setups at concentrations of ~18 mg/mL. Fine screens around initial screening conditions resulted in many isomorphous crystals. Mother liquor contained 50 mM HEPES pH 7.0 (Applichem), 100 mM HEPES pH 7.6, 100 mM NaCl (Sigma), 5 mM MgCl_2_ (Merck), 1 mM EDTA (Merck), and 31–42% PEG200 (Sigma). Good quality crystals grew at 4, 12, and 18 °C. Structure determination involved de novo phasing by gadolinium-single-wavelength anomalous diffraction (SAD) and the obtained CsaC structure was then used as a search model to solve all subsequent structures. Native crystals of CsaC-WT were soaked overnight in mother liquor supplemented with 150 mM Gd-HPDO3A (“caged gadolinium”; Jena Bioscience) at 18 °C. CPS-derived oligosaccharide with DP4 (see Supplementary Fig. 14) was soaked into native wild type crystals overnight at 18 °C. Owing to a lack of an appropriate standard, the absolute concentration of the oligomer remained undetermined. The best relative concentration was determined empirically by soaking different serial dilutions of CPS-DP4. Acetyl-CoA was soaked at a concentration of 4 mM into native CsaC-H228A crystals over-night at 18 °C.

Diffraction data (Supplementary Table 4) were measured at the ESRF beamline ID30B and the DESY beamlines P13 and P14. Data were integrated using XDS^56^. The Phase problem was solved in crank2^57^. Native and soaked crystal data were solved by Molecular Replacement in Phaser^58^ with the gadolinium-soaked structure as a search model. Structures were refined by several cycles of automatic and manual refinement in Phenix^58^ and Coot^58^, respectively.

### Analytical ultracentrifugation (AUC)

AUC experiments were carried out in a Beckman Coulter ProteomeLab XL-I analytical ultracentrifuge with an An-50 Ti rotor at 20 °C using the absorption scanning optics at 280 nm and the manufacturer’s data acquisition software ProteomeLab XL-I Version 6.0 (Firmware 5.7). The stock solution of CsaC was dialyzed against 50 mM HEPES pH 7.0, 0.1 M NaCl, 5 mM MgCl_2_, and 1 mM EDTA and diluted with the same buffer. Protein extinction coefficients at 280 nm, partial specific volumes, and buffer viscosities and densities were calculated from amino acid or buffer composition, respectively, by the program SEDNTERP^59^. Protein concentrations were determined spectrophotometrically and are given in monomers (Supplementary Fig. 8). Sedimentation velocity analysis was performed at 35,000 rpm in a concentration range from 3.0 to 40.9 µM CsaC using 3 or 12 mm double sector centerpieces filled with 100 µL or 400 µL sample, respectively. For data analysis, a model for diffusion-deconvoluted differential sedimentation coefficient distributions [continuous c(s) distributions] implemented in the program SEDFIT^60^ was used. Figures were prepared using the program GUSSI^61^.

Sedimentation equilibrium experiments were performed at 7000; 9000; 12,000; and 15,000 rpm using 150 µL of 9.1 µM CsaC or 40 µL of 40.9 µM CsaC in standard 12 or 3 mm double sector centerpieces, respectively. Samples were spun at each rotor speed until no further change in concentration gradient could be observed for at least 8 h. The baseline offset was set to the buffer absorbance measured in each cell near the meniscus after sedimenting the protein for 8 h at 44,000 rpm at the end of the experiment. The molar mass was determined by globally fitting the data obtained at all rotor speeds and concentrations to a single species model, similar as described previously^62^.

### Bioinformatics analyses

Blastp^63^ (protein-protein BLAST) searches were performed against the non-redundant protein sequences database both including and excluding Gram-positive bacteria, yielding hits with 25–40% sequence identity. Multiple-sequence alignments were performed with Clustal omega^64^ and annotated with Jalview^65^ (Supplementary Fig. 27), and with CLUSTALW implemented in MEGAX^66^ (Supplementary Data 1).

### Reporting summary

Further information on research design is available in the Nature Research Reporting Summary linked to this article.

## Supplementary information

Supplementary Information

Description of Additional Supplementary Files

Supplementary Data 1

Reporting Summary

## Data Availability

The crystallographic datasets reported herein have been deposited in the PDB repository under accession codes 6YUO, 6YUV, 6YUS, 6YUQ. Source data for Figs. 3, 5 and Supplementary Fig. 14 are provided with this paper. Raw NMR and mass spectrometry data are available from the corresponding authors upon request. Source data are provided with this paper.

## Source data

Source Data
