# Supplementary material for: Structural and mechanistic basis of capsule O-acetylation in Neisseria meningitidis serogroup A
Source: Nat Commun. 2020 Sep 18;11:4723. doi: 10.1038/s41467-020-18464-y (PMC7501274; doi:10.1038/s41467-020-18464-y)
Supplement: Supplementary file 1 — Supplementary Information [file 41467_2020_18464_MOESM1_ESM.pdf]

Supplementary Information

Structural and mechanistic basis of capsule *O*-acetylation in *Neisseria meningitidis* serogroup A

Timm Fiebig, Johannes T Cramer et al.

1. Supplementary Tables (Supplementary Tables 1-5)

2. Supplementary Figures (Supplementary Figs. 1-27)

3. Supplementary Notes (Supplementary Note 1)

4. Supplementary References

Supplementary Tables

Supplementary Table 1. Observed ^{1}H, ^{13}C and ^{31}P chemical shifts of non-*O*-acetylated NmA capsular polymer, referenced to 2,2-dimethyl-2-silapentanesulfonic acid (DSS).

Atom	Internal αManNAc-6-P moiety ⑦	αManNAc-6-P moiety at non-reducing end ⑥	αManNAc-6-P moiety at reducing end ⑨	βManNAc-6-P moiety at reducing end ⑤	Free αManNAc (Values from Coxon *et al.*[1])[b]	Free βManNAc (Values from Coxon *et al.*[1])[b]
H1	5.422	5.407	5.126	5.046	5.121	5.028
H2	4.448	4.443	4.333	4.462	4.319	4.451
H3	4.137	4.131	4.060	3.842	4.047	3.828
H4	3.780	3.857	3.690	3.552	3.620	3.522
H5	3.989	3.923	3.968	3.549	3.863	3.416
H6	4.240	4.177	4.186	4.181	~3.939	3.890
H6'	4.173	3.978	4.138	4.138	~3.939	3.812
H8	2.073	2.067	2.058	2.103	2.048	2.092
C1	97.9	98.0	96.0	95.8	96.0	95.5
C2	55.9	55.9	55.9	56.7	56.1	56.9
C3	71.3	70.9	71.6	74.7	71.7	74.9
C4	68.7	68.5	69.3	69.2	69.6	69.4
C5	75.0	75.7	73.6	77.8	74.8	79.2
C6	67.2	65.4	67.6[a]	67.6[a]	63.2	63.2
C7 (NAc)	177.7	177.7[a]	177.7[a]	178.5	177.6	178.6
C8 (NAc)	24.8	24.8	24.8	24.8	24.8	24.9
6P	-5.31	0.63	-5.21[a]	-5.21[a]	–	–
1P	-5.31	-5.20	–	–	–	–
$^{3}J_{H1H2}$	1.4 Hz	n.d.[d]	1.3 Hz	1.6 Hz	1.7 Hz (1.3 Hz)[c]	1.7 Hz (1.6 Hz)[c]
$^{3}J_{H1P}$	7.4 Hz	n.d.[d]	–	–		

[a]overlapping resonances

[b]referencing was slightly different, an offset of 2.1 ppm to all ^{13}C values reported in Coxon *et al.* [1] was determined

[c]values reported by Pan *et al.*[2].

[d]not determined (n.d.), either due to overlap or weak signal intensity

Supplementary Table 2. Observed ^{1}H, ^{13}C and ^{31}P chemical shifts of the repeating units of *O*-acetylated NmA capsular polymer, referenced to 2,2-dimethyl-2-silapentanesulfonic acid (DSS).

Atom	Internal 3OAc-αManNAc-6-P moiety ①	Internal 3OAc-αManNAc-6-P moiety ②	Internal 4OAc-αManNAc-6-P moiety ③	Internal 4OAc-αManNAc-6-P moiety ④	Internal αManNAc-6-P moiety ⑦	Internal αManNAc-6-P moiety ⑧
H1	5.479	5.426	5.461	5.415	5.432	5.381
H2	4.597	4.556	4.513	4.508	4.457	4.454
H3	5.206	5.183	4.341	4.330	4.149	4.133
H4	4.008	4.005	5.203	n.d.[a]	3.792	n.d.[a]
H5	4.120	4.079	4.208	4.158	4.007	3.965
H6	4.290	4.291	4.111	4.107	4.259	n.d.[a]
H6'	4.213	4.193	4.036	4.034	4.197	n.d.[a]
H8	2.079	2.076	2.110	n.d.[a]	2.092	n.d.[a]
H10	2.110	2.111	2.187	n.d.[a]	–	–
C1	97.5	97.7	97.7	n.d.[a]	97.9	97.9
C2	53.4	53.3	55.9	55.9	55.9	55.9
C3	74.6	74.7	69.6	69.6	71.3	71.3
C4	66.1	66.1	71.0	n.d.[a]	68.7	n.d.[a]
C5	74.9	74.8	72.8	72.6	75.1	75.1
C6	67.0	67.1	66.9	n.d.[a]	67.2	n.d.[a]
C7 (NAc)	177.3	177.4	177.6	n.d.[a]	177.6	n.d.[a]
C8 (NAc)	24.6	24.5	24.9	n.d.[a]	24.8	n.d.[a]
C9 (OAc)	176.1	176.1	175.6	n.d.[a]	–	–
C10 (OAc)	23.2	23.2	23.2	n.d.[a]	–	–
6P	-5.28	n.d.[a]	-5.84	n.d.[a]	-5.20	n.d.[a]
1P	-5.28	-5.88	(-5.24)[b]	-5.74	-5.20	-5.72

[a]not determined (n.d.), either due to overlap or weak signal intensity

[b]overlapping resonances

Supplementary Table 3. Observed ^{1}H, ^{13}C and ^{31}P chemical shifts of the termini of *O*-acetylated NmA capsular polymer, referenced to 2,2-dimethyl-2-silapentanesulfonic acid (DSS).

Atom	αManNAc-6-P moiety at non-reducing end ⑥	αManNAc-6-P moiety at reducing end ⑨	βManNAc-6-P moiety at reducing end ⑤
H1	5.418	5.137	5.061
H2	4.458	4.345	4.478
H3	4.157	4.077	3.855
H4	3.880	3.696	3.568
H5	3.938	3.987	3.563
H6	4.192	4.200	4.206
H6'	3.972	4.151	4.147
H8	n.d.[a]	n.d.[a]	n.d.[a]
C1	(97.9)[b]	95.9	95.8
C2	(55.9)[b]	55.9	56.8
C3	n.d.[a]	71.5	n.d.[a]
C4	68.6	69.4	n.d.[a]
C5	75.9	n.d.[a]	n.d.[a]
C6	n.d.[a]	67.3	n.d.[a]
C7 (NAc)	n.d.[a]	n.d.[a]	n.d.[a]
C8 (NAc)	n.d.[a]	n.d.[a]	n.d.[a]
6P	1.04	-5.19	n.d.[a]
1P	-5.18	–	–

[a] not determined (n.d.), either due to overlap or signal intensity

[b] overlapping resonances

Supplementary Table 4. Data collection and refinement statistics.

	6YUO: WT+Gd	6YUV: native WT	6YUS: H228A+CoA	6YUQ: WT+CPS
**Data collection**				
Space group	$P\,4_1\,2_1\,2$	$P\,4_1\,2_1\,2$	$P\,4_1\,2_1\,2$	$P\,4_1\,2_1\,2$
Cell dimensions				
a, *b*, *c* (Å)	132.9, 132.9, 69.3	138.3, 138.3, 70.6	136.7, 136.7, 70.7	137.5, 137.5, 70.3
α, β, γ (°)	90, 90, 90	90, 90, 90	90, 90, 90	90, 90, 90
Wilson B (Å²)	43.7	43.0	63.9	64.2
Resolution range (high-resolution shell) (Å)	50-2.20(2.32-2.20)	50-2.00 (2.07-2.00)	50-2.00 (2.08-2.00)	50-1.95 (2.02-1.95)
R_{merge}	0.081 (0.896)	0.048 (0.694)	0.080 (4.052)	0.060 (2.413)
I / σI	25.2 (4.1)	36.2 (5.0)	19.6 (0.5)	25.1 (1.4)
$cc_{1/2}$	1.00 (0.91)	1.00 (0.98)	1.00 (0.22)	99.8 (0.64)
Completeness (%)	89.9 (89.6)	99.9 (99.9)	100.0 (99.9)	99.9 (99.9)
Redundancy	24.5 (25.3)	22.8 (22.9)	23.5 (15.6)	26.2 (25.6)
**Refinement**				
Resolution (Å)	2.2	2.0	2.0	1.95
No. reflections	28,548	46,709	44,759	49,462
R_{work} / R_{free}	0.182 / 0.219	0.179 / 0.207	0.198 / 0.240	0.210 / 0.221
No. atoms	3,952	4,092	4,022	4,037
Protein	3,857	3,915	3,868	3,898
Ligand/ion	15	12	114	92
Water	80	165	40	47
B-factors (Å²)	55.1	55.4	77.5	71.3
Protein	54.9	55.4	77.3	71.1
Ligand/ion	90.3	64.1	89.5	85.0
Water	56.5	55.7	63.4	63.2
R.m.s. deviations				
Bond lengths (Å)	0.003	0.008	0.010	0.006
Bond angles (°)	0.61	0.81	1.14	0.75

Note: Statistics are based on one crystal per dataset

Supplementary Table 5: Plasmids and primers used in this study. The plasmids for the expression of CsaC are based on pET22b (Novagen).

#	Plasmid	Primer	Primer sequence
**4182**	pStrep-CsaC-His	FF58	5'-GCGGATCCTTATCTAATTTAAAAACAGG-3'
		FF57	5'-CCGCTCGAGTATATTTTGGATTATGGT-3'
**4815**	pStrep-CsaC-Ser114Ala-His	AB78	5'-CCTTTTGGGTTCAGCTAAAGGTGGCGTTGGCGC-3'
		AB79	5'-GCGCCAACGCCACCTTTAGCTGAACCCAAAAGG-3'
**4816**	pStrep-CsaC-Asp198Ala-His	AB80	5'-CTTGCGGAAAAGATGCTTCATATCATTTAAATGAATTAG-3'
		AB81	5'-CTAATTCATTTAAATGATATGAAGCATCTTTTCCGCAAG-3'
**4817**	pStrep-CsaC-His228Ala-His	AB82	5'-CCAAACTAATTTCTGGCGGGGCTGATAATGAAGCAATTGC-3'
		AB83	5'-GCAATTGCTTCATTATCAGCCCCGCCAGAAATTAGTTTGG-3'
**5082**	pStrep-CsaC-His201Ala-His	AB99	5'-GCGGAAAAGATGATTCATATGCTTTAAATGAATTAGAAATTC-3'
		AB100	5'-GAATTTCTAATTCATTTAAAGCATATGAATCATCTTTTCCGC-3'
**5374**	pStrep-CsaC-Y144A-His	AB134	5'-GCCAAATTAGCAGATGCTATCAAAACACGCTCG-3'
		AB135	5'-CGAGCGTGTTTTGATAGCATCTGCTAATTTGGC-3'
**5375**	pStrep-CsaC-R148A-His	AB136	5'-GCAGATTATATCAAAACAGCCTCGAAAACCATTCTTTC-3'
		AB137	5'-GAAAGAATGGTTTTCGAGGCTGTTTTGATATAATCTGC-3'
**5376**	pStrep-CsaC-R148K-His	AB138	5'-CCAAATTAGCAGATTATATCAAAACAAAGTCGAAAACCATTCTTTC-3'
		AB139	5'-GAAAGAATGGTTTTCGACTTTGTTTTGATATAATCTGCTAATTTGG-3'
**4875**	pCsaC-His	AB90	5'-GCATCTCATATGTTATCTAATTTAAAAACAGG-3'
		FF57	5'-CCGCTCGAGTATATTTTGGATTATGGT-3'
**5424**	pCsaC-H228A-His	AB82	5'-CCAAACTAATTTCTGGCGGGGCTGATAATGAAGCAATTGC-3'
		AB83	5'-GCAATTGCTTCATTATCAGCCCCGCCAGAAATTAGTTTGG-3'
**5697**	pStrep-CsaC-Q138A-His	AB198	5'-TATAATTATTAATGCTCCTGCGGCCAAATTAGCAGATTATATCAAAACACGCTCGAAAACC-3'
		AB199	5'-TAATCTGCTAATTTGGCCGCAGGAGCATTAATAATTATATTAGGATAATTATATGTAAGACCG-3'
**5698**	pStrep-CsaC-Q138A-H228A-His	AB198	5'-TATAATTATTAATGCTCCTGCGGCCAAATTAGCAGATTATATCAAAACACGCTCGAAAACC-3'
		AB199	5'-TAATCTGCTAATTTGGCCGCAGGAGCATTAATAATTATATTAGGATAATTATATGTAAGACCG-3'

Supplementary Figures

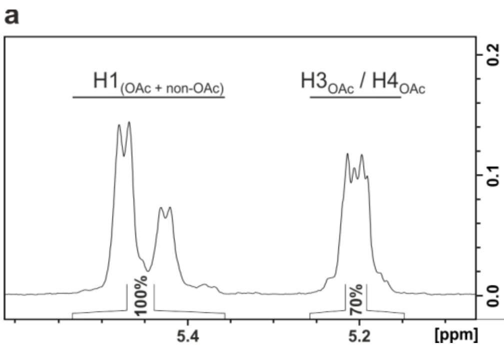

b

AcCoA / ManNAc	% O-acetylation
1	70
2	87
4	92

Supplementary Figure 1. Tailoring CsaC-mediated *O*-acetylation levels. a, Representative example of a 1D ^{1}H NMR spectrum of *O*-acetylated NmA-CPS. The degree of *O*-acetylation of the NmA-CPS was determined according to a convenient, previously published method[3], which is based on integrating the ^{1}H NMR signals of $H3_{OAc}$ and $H4_{OAc}$. The two overlapping signals center at ~5.2 ppm and are thus well isolated from signals of non-*O*-acetylated ring protons[3]. Through comparison to the integrated signals resulting from the anomeric proton of both *O*-acetylated and non-*O*-acetylated moieties ($H1_{(OAc+nonOAc)}$, set to 100%) the percentage of *O*-acetylated ManNAc units is obtained. Precise assignment of the H1 signals by 2D NMR and an in-depth characterization of the *O*-acetylation pattern is shown in Fig. 2. A precise determination of the *O*-acetylation pattern by 2D NMR is described in the **Supplementary Note 1**. **b,** Percentage of CsaC-mediated *O*-acetylation at donor (acetyl-CoA) to acceptor (ManNAc) ratios of 1:1 (7 mM : 7 mM), 2:1 (14 mM : 7 mM) and 4:1 (28 mM : 7 mM), determined as described in (**a**). The percentage of *O*-acetylated ManNAc moieties increased with increasing donor to acceptor ratios yielding *O*-acetylation levels comparable to those reported for CPS harvested from NmA cultures[3–5]. It is important to note that the level of *O*-acetylation is generally below 100% for polymers harvested from pathogen cultures, not only for NmA, but also for other meningococcal serogroups[3,4] and other pathogenic bacteria[6,7].

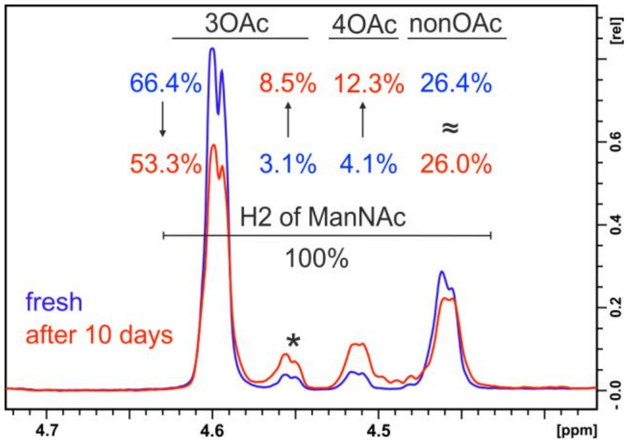

Supplementary Figure 2. ^{1}H NMR analysis of *O*-acetyl group migration in water. The integral of the entire H2 region was set to 100% (see bar) and the relative amounts of the 3OAc-, 4OAc- and nonOAc moieties were calculated based on the area of each individual peak as indicated. In the beginning of the experiment (fresh), 4% of the ManNAc residues were already *O*-acetylated in position C4. During two days of storage at room temperature no change of the *O*-acetylation pattern was seen (not shown) and the sample was transferred to 45°C. Thereafter, 4-*O*-acetylation steadily increased on the expense of 3-*O*-acetylation (not shown) until, on day 10, 12% of all residues were 4-*O*-acetylated (red chromatogram). *indicates 3-*O*-acetylated residues connected to 4-*O*-acetylated residues as described in Lemercinier and Jones (1996)[3] and further defined in Fig. 2 and the corresponding results section.

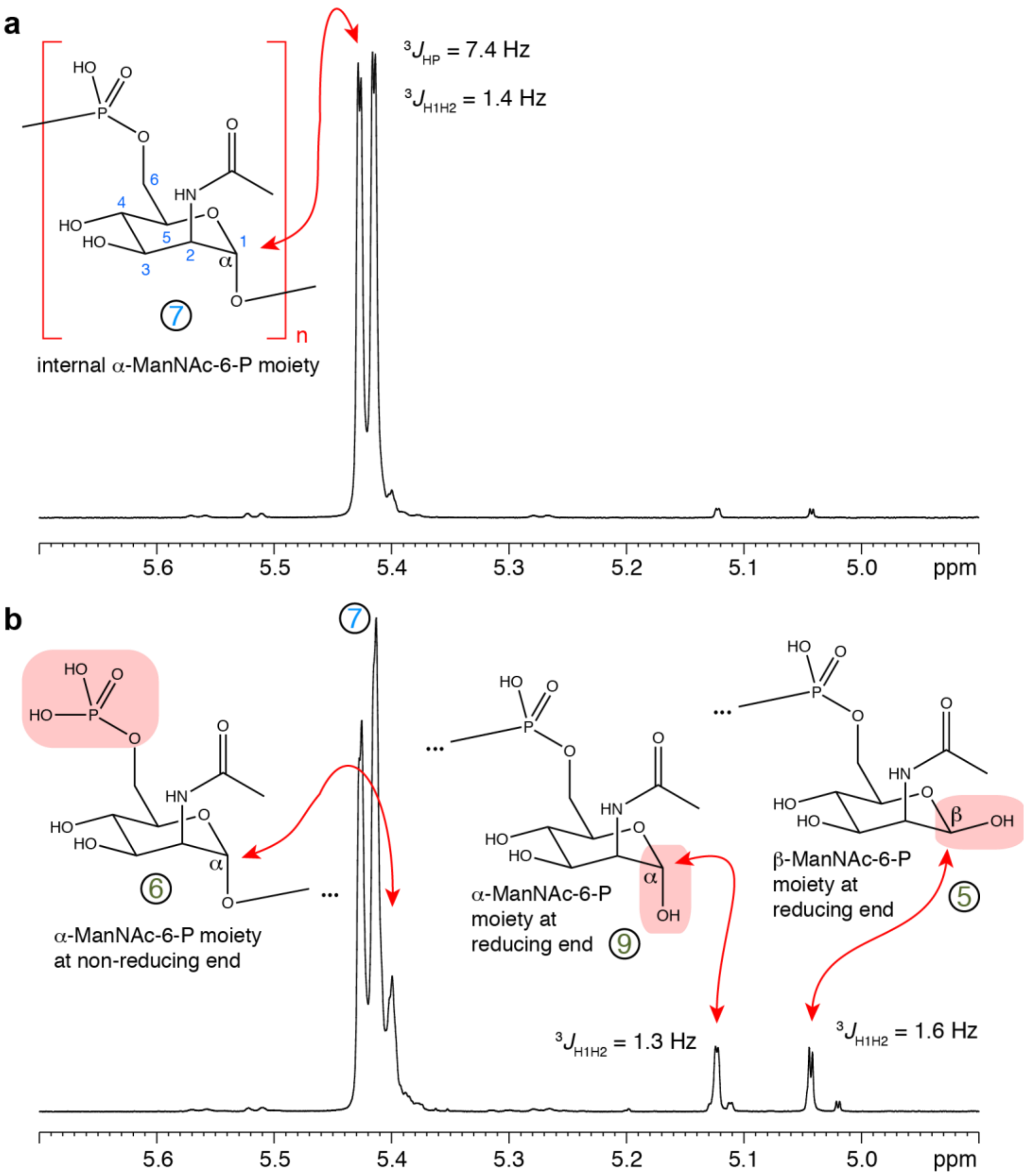

Supplementary Figure 3. ^{1}H NMR spectra of the non-*O*-acetylated NmA-CPS. **a,** Non-hydrolyzed sample and **b,** partially hydrolyzed sample. Chemical structures show spin systems ⑥, ⑨, ⑤ from left to right.

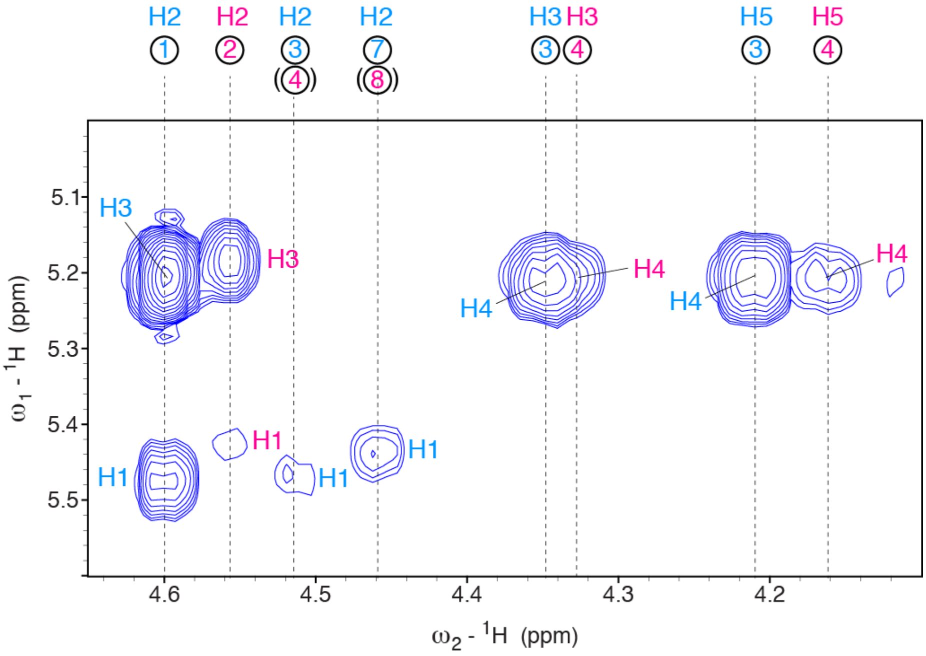

Supplementary Figure 4. ^{1}H-^{1}H COSY spectrum of *O*-acetylated NmA-CPS. Spin systems and resonances are indicated above or next to the signals (for the ω_1 dimension).

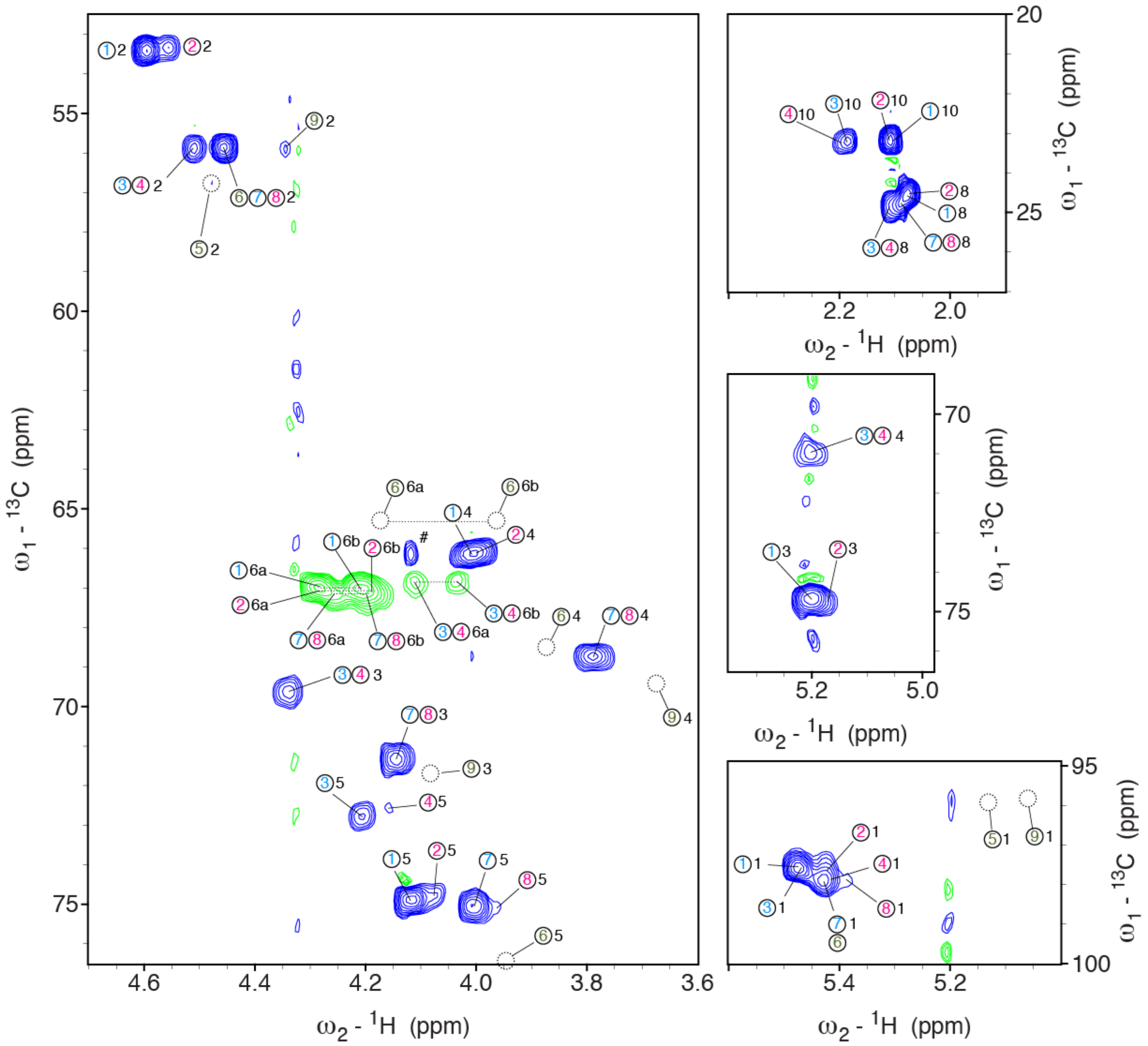

Supplementary Figure 5. ^{13}C-^{1}H HSQC spectrum of *O*-acetylated NmA-CPS. Due to the multiplicity-editing, positive signals (blue) indicate CH groups and negative signals (green) CH_2 groups. For each signal, the spin system is shown as circled number and the position within the moiety is indicated, for example "⑧1", refers to H1/C1 of spin system ⑧. Very weak signals below the threshold of the shown contour levels are indicated by dotted circles.

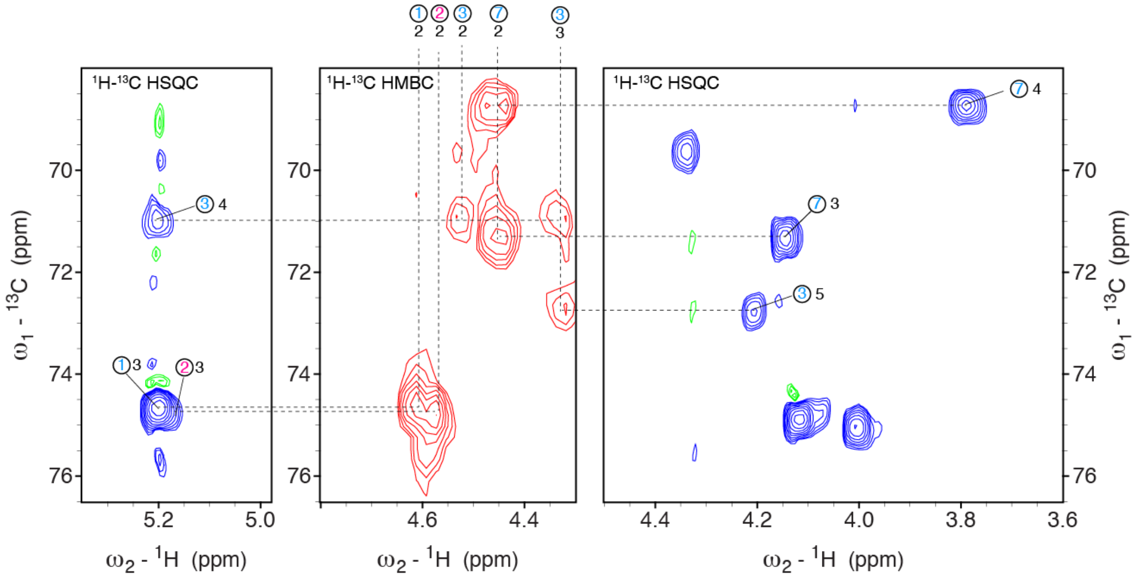

Supplementary Figure 6. ^{13}C-^{1}H HMBC spectrum of *O*-acetylated NmA-CPS aligned with relevant regions of a ^{13}C-^{1}H HSQC spectrum. The ^{13}C chemical shifts (C3 of ①, 74.6 ppm; C4 of ③, 71.0 ppm) deduced from the H3-C3 and H4-C4 correlations of the ^{13}C-^{1}H HSQC experiment (left panel) could be unambiguously correlated to H2 of the 3-*O*-acetylated and 4-*O*-acetylated spin systems ① and ③, respectively, in the ^{13}C-^{1}H HMBC spectrum (middle panel).

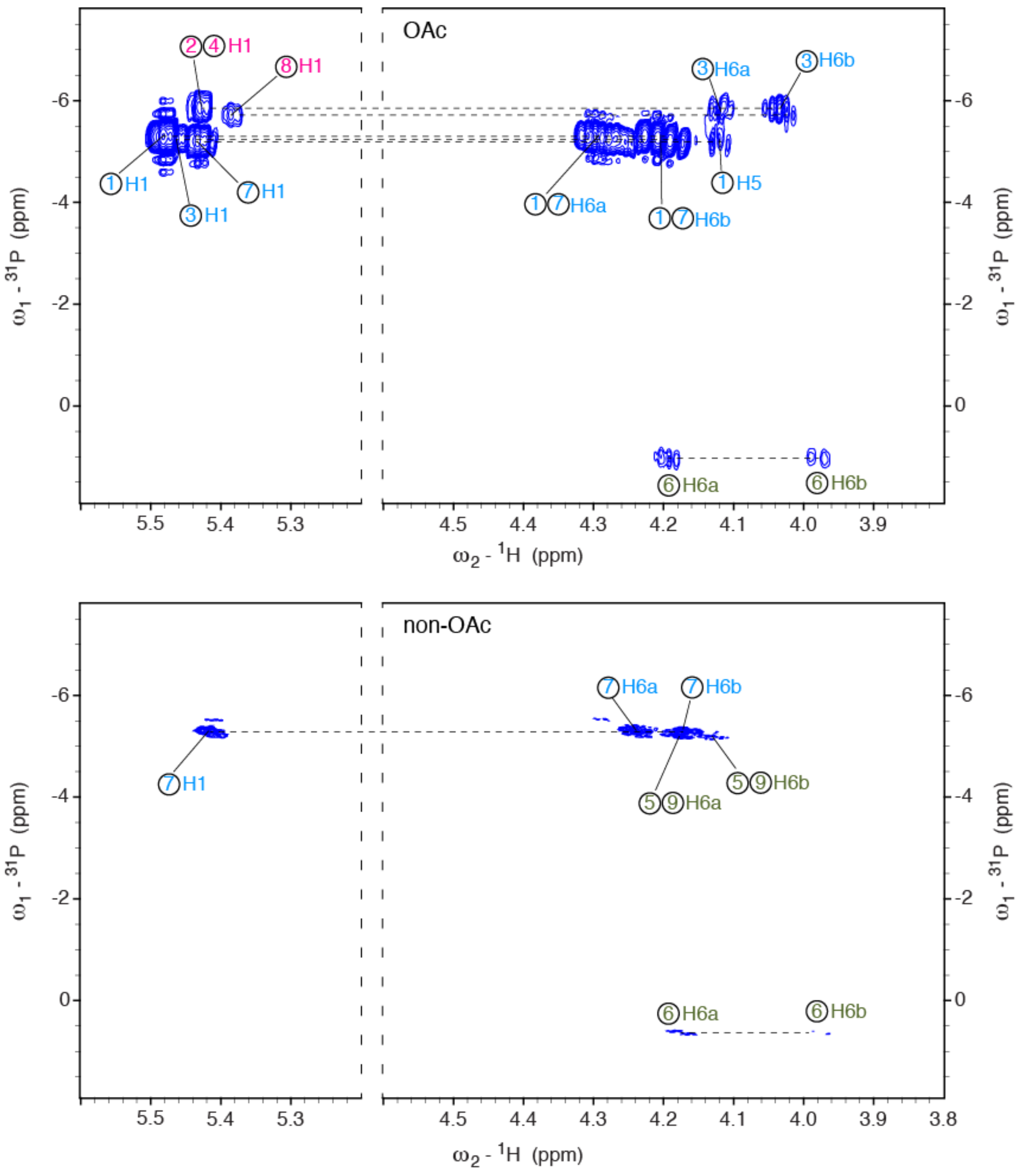

Supplementary Figure 7. ^{31}P-^{1}H HMBC spectra of *O*-acetylated (top) and non-*O*-acetylated (bottom) NmA-CPS. ^{31}P chemical shifts of phosphomonoesters are typically ~5 ppm downfield compared to phosphodiester resonances[8]. The spectrum at the bottom was recorded with 32 scans, a recycle delay of 1.5 sec, 4096×320 points and spectral widths of 10.0 ppm × 9.0 ppm with a duration of 5 hours 40 min.

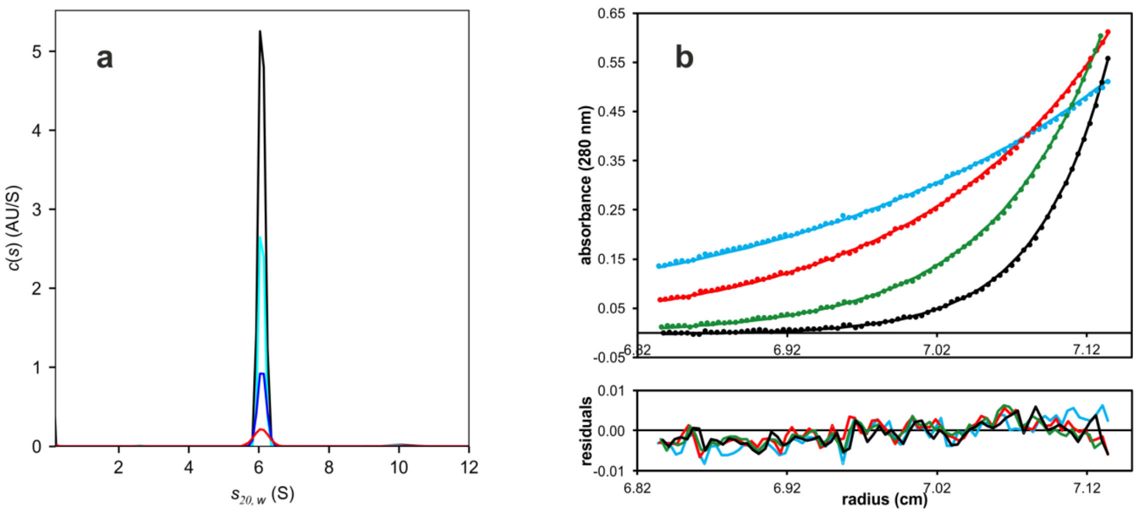

Supplementary Figure 8. CsaC forms stable tetramers in solution as shown by analytical ultracentrifugation (AUC). a, Sedimentation velocity analyses of 3.0 µM (red), 9.1 µM (blue), 18.6 µM (cyan) and 40.9 µM (black) CsaC. Independent of protein concentration, CsaC sediments as a single species with $s_{20,\ w}$ = 6.1 S. For ease of comparison, all sedimentation coefficient distributions have been converted to a path length of 12 mm. **b**, Concentration gradients obtained for 40.9 µM CsaC in sedimentation equilibrium centrifugation were measured at 7,000 rpm (cyan), 9,000 rpm (red), 12,000 rpm (green) and 15,000 rpm (black). These data were fitted together with the data obtained for 9.1 µM CsaC at the same rotor speeds (data not shown) using a single species model. The global fit (solid lines) yielded a molar mass of 113 kg/mol. As the molar mass of monomeric CsaC is 29.3 kg/mol, the protein forms stable tetramers in solution. The lower panel shows the difference between the calculated and the measured absorbance values as a function of radial position (residuals).

In order to determine the oligomerization state of CsaC in solution, 3.0 to 40.9 µM of the protein were subjected to sedimentation velocity analysis in an AUC. Independent of protein concentration, CsaC sedimented as a single species with a sedimentation coefficient $s_{20,\ w}$= 6.1 S. (**Supplementary Fig. 8a**). From the continuous c(s) distribution model[9], a molar mass of about 114 kg/mol was obtained from sedimentation coefficient and diffusion broadening of the sedimenting boundary. Since the molar mass of the monomer as calculated from amino acid composition is 29.3 kg/mol, CsaC forms stable tetramers in solution. This result was also confirmed by sedimentation equilibrium experiments, where global analysis of the concentration gradients obtained at 4 rotor speeds and two protein concentrations yielded a molar mass of 113 kg/mol (**Supplementary Fig. 8b**). Comparison of the obtained sedimentation coefficient with the s-value calculated for an unhydrated, spherical protein of the same mass as the CsaC tetramer yielded a frictional ratio of 1.36. Since for a spherical, hydrated protein a frictional ratio of 1.1 to 1.2 is expected[10], the CsaC tetramer deviates substantially from the shape of a sphere. This is consistent with the structure of the potential tetramer as identified by x-ray crystallography (Fig. 4a, **Supplementary Fig.** 9). Hydrodynamic bead modelling of this high resolution structure with the program SoMo[11], using a SoMo overlap bead model followed by ZENO calculation, yielded $s_{20,\ w}$ = 6.3 S. The obtained deviation from the experimental result is within the error limits of the method[12].

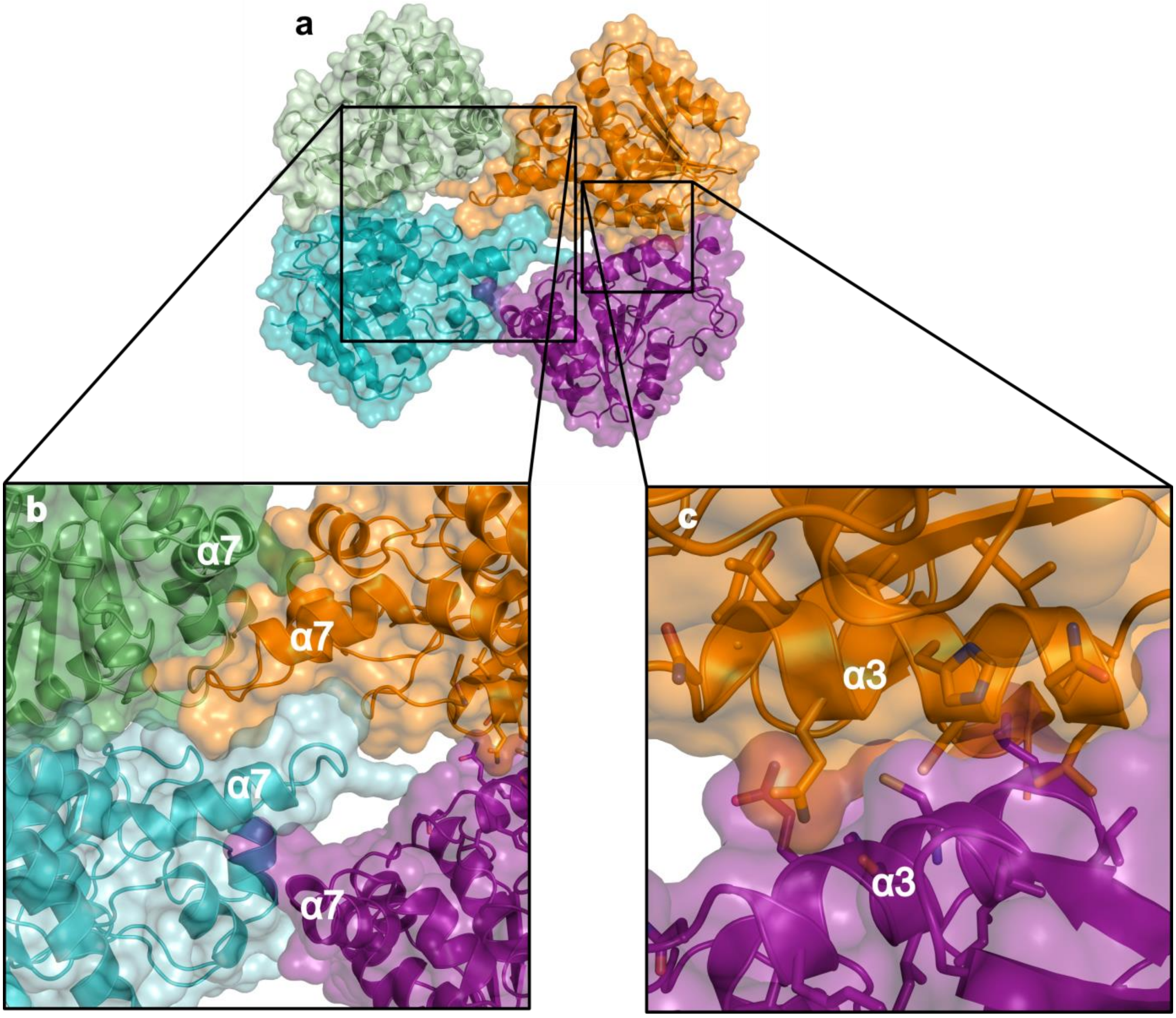

Supplementary Figure 9. Oligomerization interface of CsaC. a, Assembly of the CsaC tetramer. **b**, Close-up of the tetrameric interface, mainly facilitated by α-helix α7. **c**, Close-up of the dimerization interface between two protomers, mainly facilitated by α-helix α3.

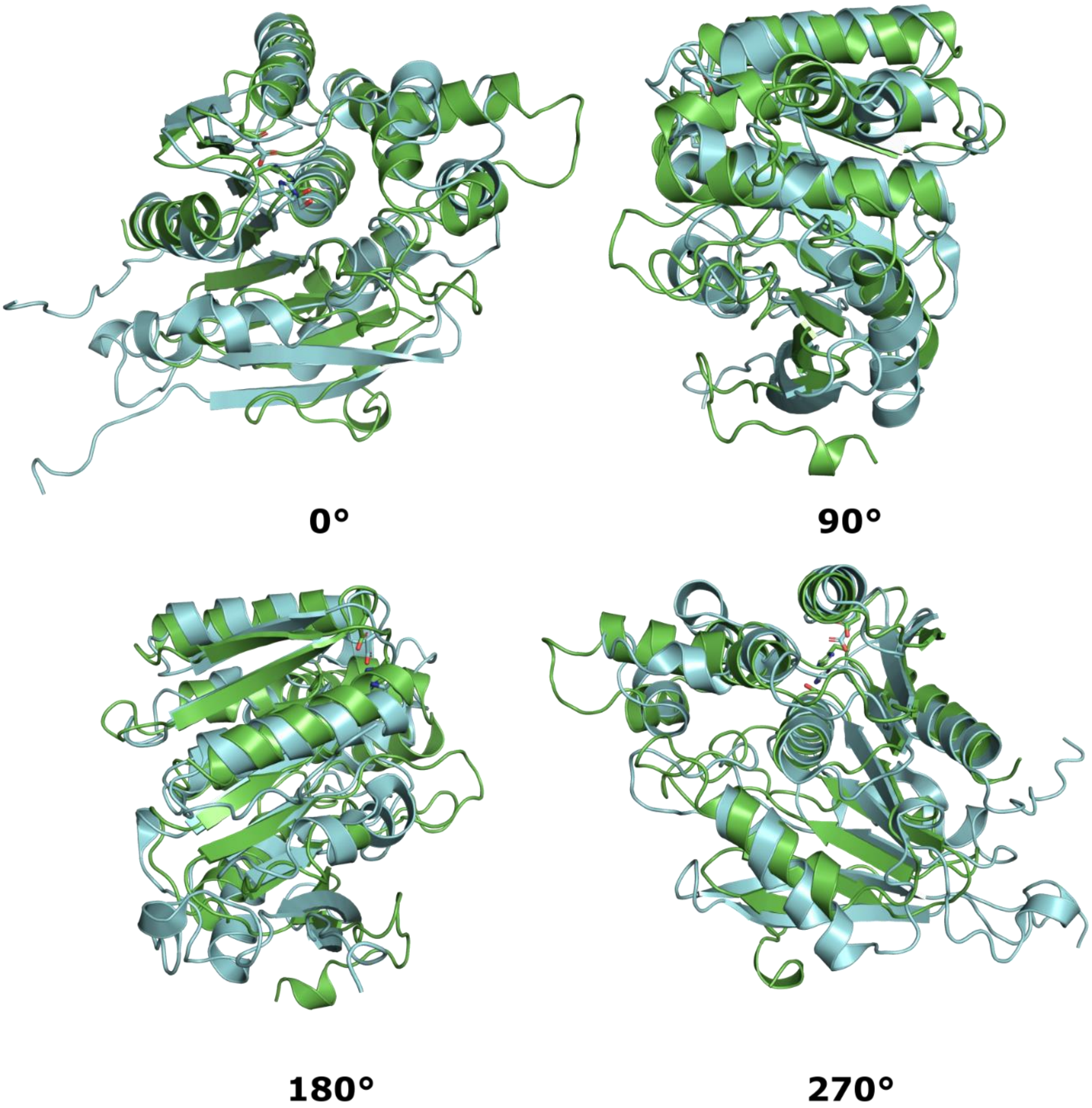

Supplementary Figure 10. Fold and secondary structure comparison of CsaC. CsaC (green) in apo form (chain A) with top DALI search hit esterase A from *Streptococcus pyogenes* (cyan) (pdb: 4rot, DALI z-score = 18.4, r.m.s.d. = 2.59 Å). Residues of the catalytic triad are shown in stick representation.

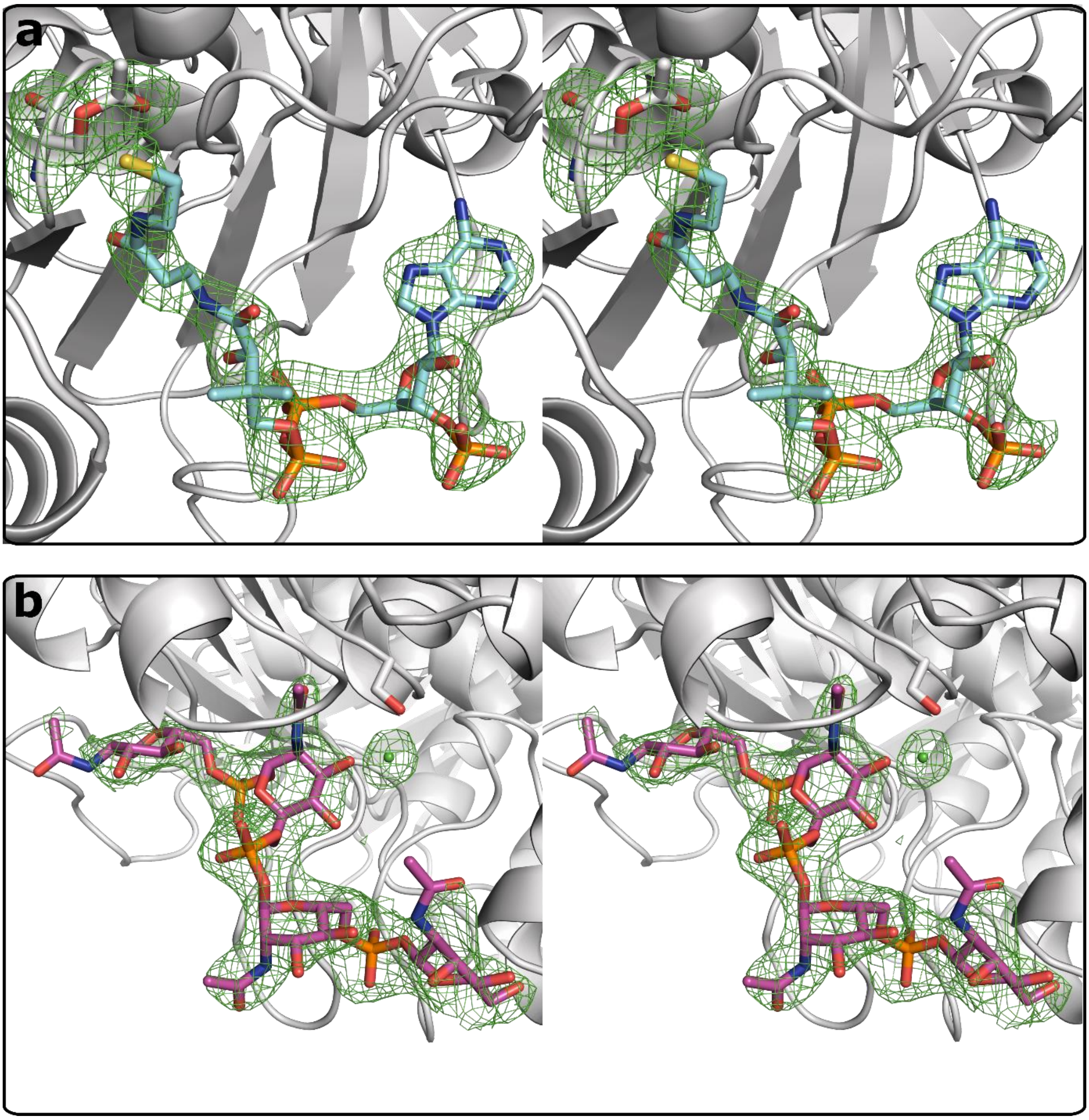

Supplementary Figure 11. **Cross-eyed stereo of Fo-Fc electron density difference maps contoured at 3 σ** omitting **a**, CoA (cyan) and acetyl-Ser in the active site of CsaC-H228A and **b**, CPS-DP4 (magenta) and chloride (green) in the active site of wild type CsaC.

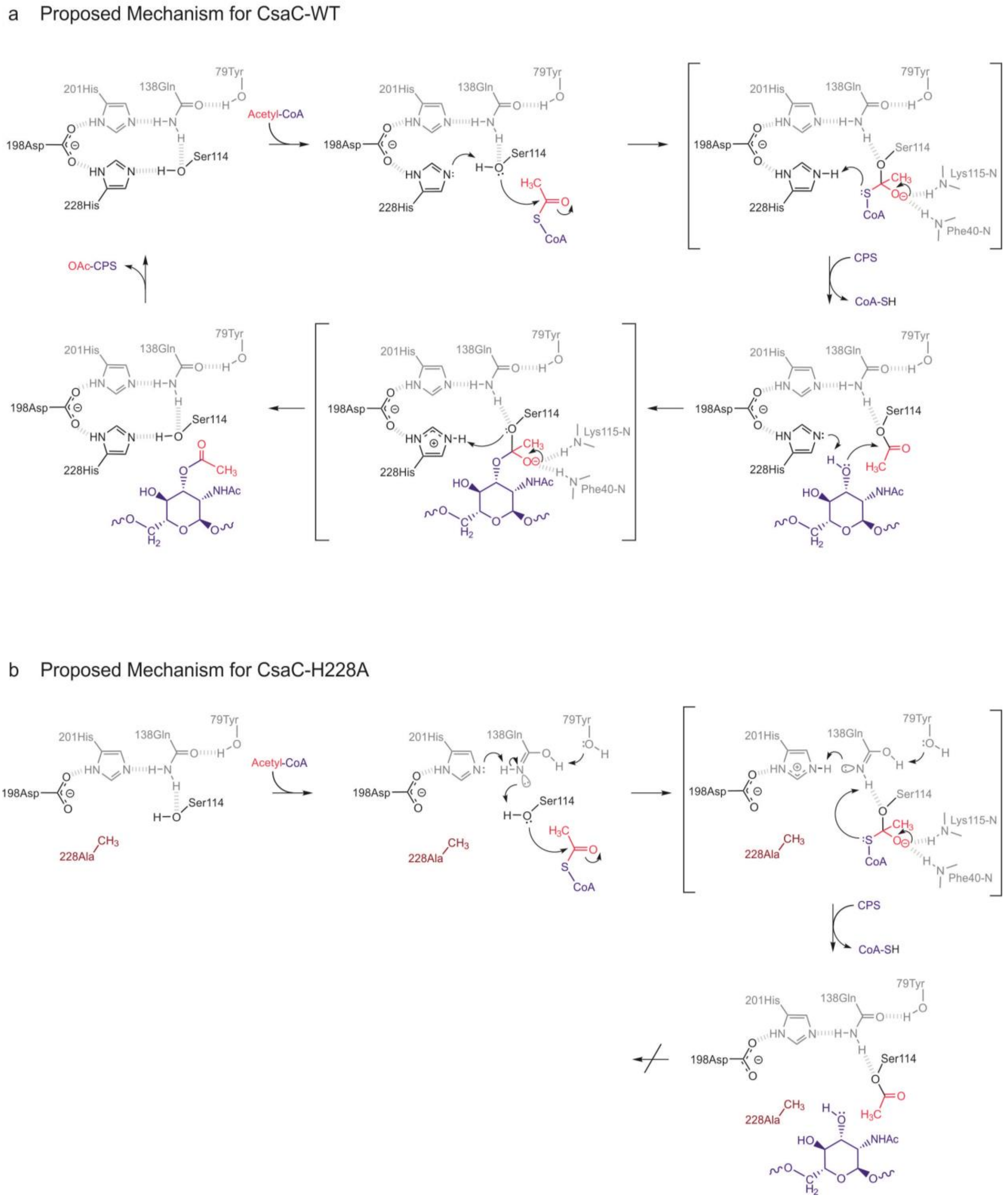

Supplementary Figure 12: Proposed reaction mechanism for **a,** CsaC-WT and **b,** CsaC-H228A. **a,** The reaction of CsaC-WT proceeds through six consecutive steps, starting with donor substrate binding, nucleophilic attack of the carbonyl group of acetyl-CoA by S114 and formation of a first tetrahedral intermediate. H228 acts as general base to deprotonate S114. The main-chain amides of F40 and K115, the canonical components of the oxyanion hole (**Supplementary Fig.** 18), stabilize the tetrahedral intermediate anion. Decomposition of the tetrahedral intermediate leads to the release of free CoA and the formation of an acetyl-enzyme adduct. Release of the first product allows binding of the acceptor polysaccharide with one ManNAc moiety positioned in the substrate entry site. H228 acts as general base to deprotonate the acceptor hydroxyl group, which allows

nucleophilic attack of the carbonyl carbon of the acetyl-enzyme, yielding a second tetrahedral intermediate. Collaps of the second tetrahedral intermediate results in the release of 3-*O*-acetylated CPS and the regeneration of free enzyme (for the sake of clearity, the main-chain amides of F40 and K115 are depicted only in schemes showing a tetrahedral intermediate).

b, In the CsaC-H228A mutant, Q138 can substitute H228 in the first half-reaction. In the absence of H228, the hydrogen bond network of H201, Q138 and Y79 forms a proton relay system that stabilizes the imidic acid tautomer of Q138. The imidic acid form of Q138 can act as a base to deprotonate the triad serine S114. This allows nucleophilic attack of the carbonyl group of the incoming acetyl-CoA and the formation of the first tetrahedral intermediate, which finally yields the acetyl-enzyme adduct. Because Q138 is located in the back of the active site - opposite to the substrate entry site (Fig. 5c) - Q138 is too distantly positioned to deprotonate the acceptor hydroxyl in the second half-reaction. The sugar hydroxyl group remains a poor nucleophile, unable to attack the carbonyl carbon of the acetyl-enzyme adduct. This prevents hydrolysis of the acetyl-adduct, which is thus trapped in the absence of H228.

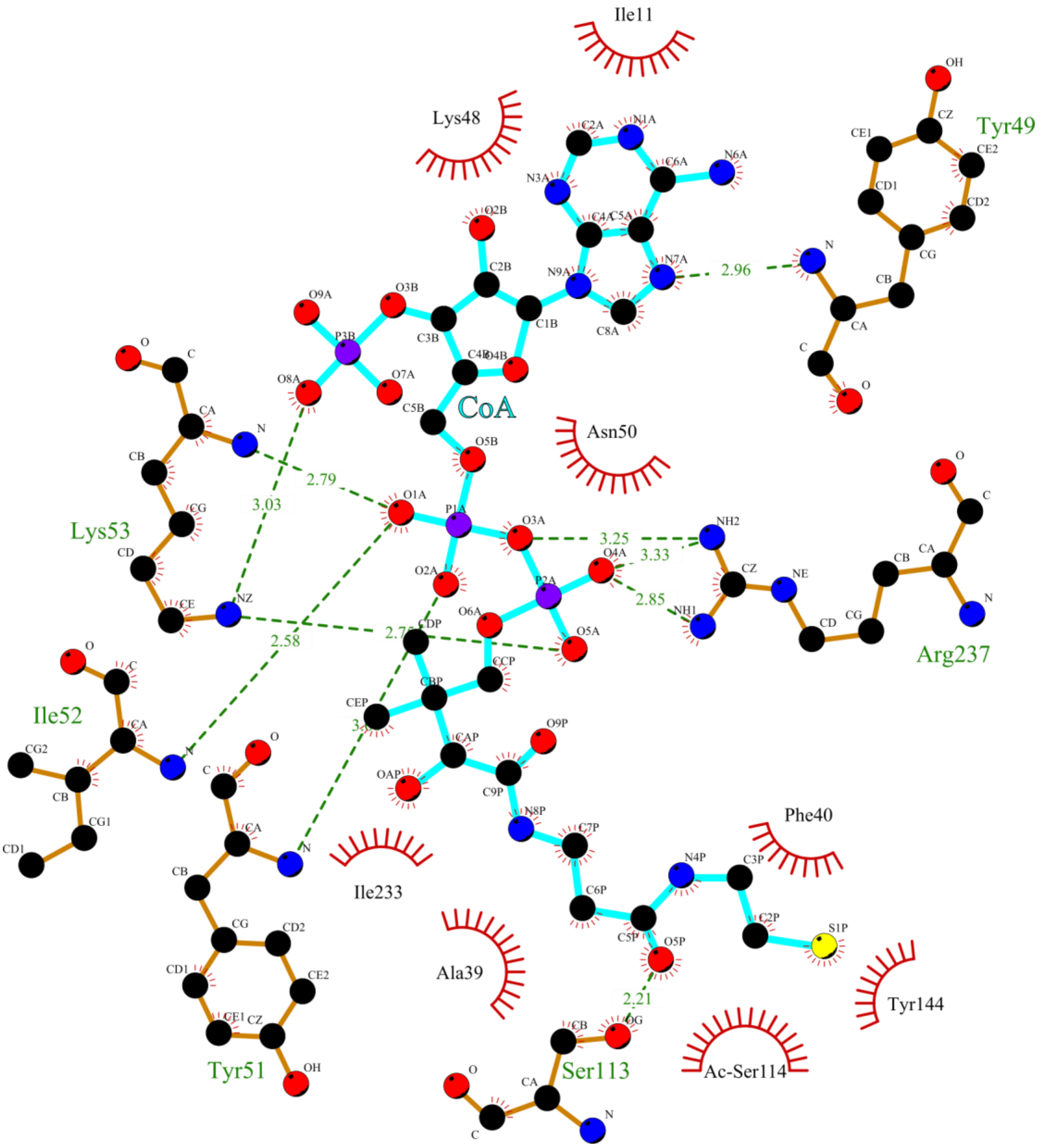

Supplementary Figure 13. LigPlot+[13] of CoA (cyan) in the CsaC-H228A structure chain B.

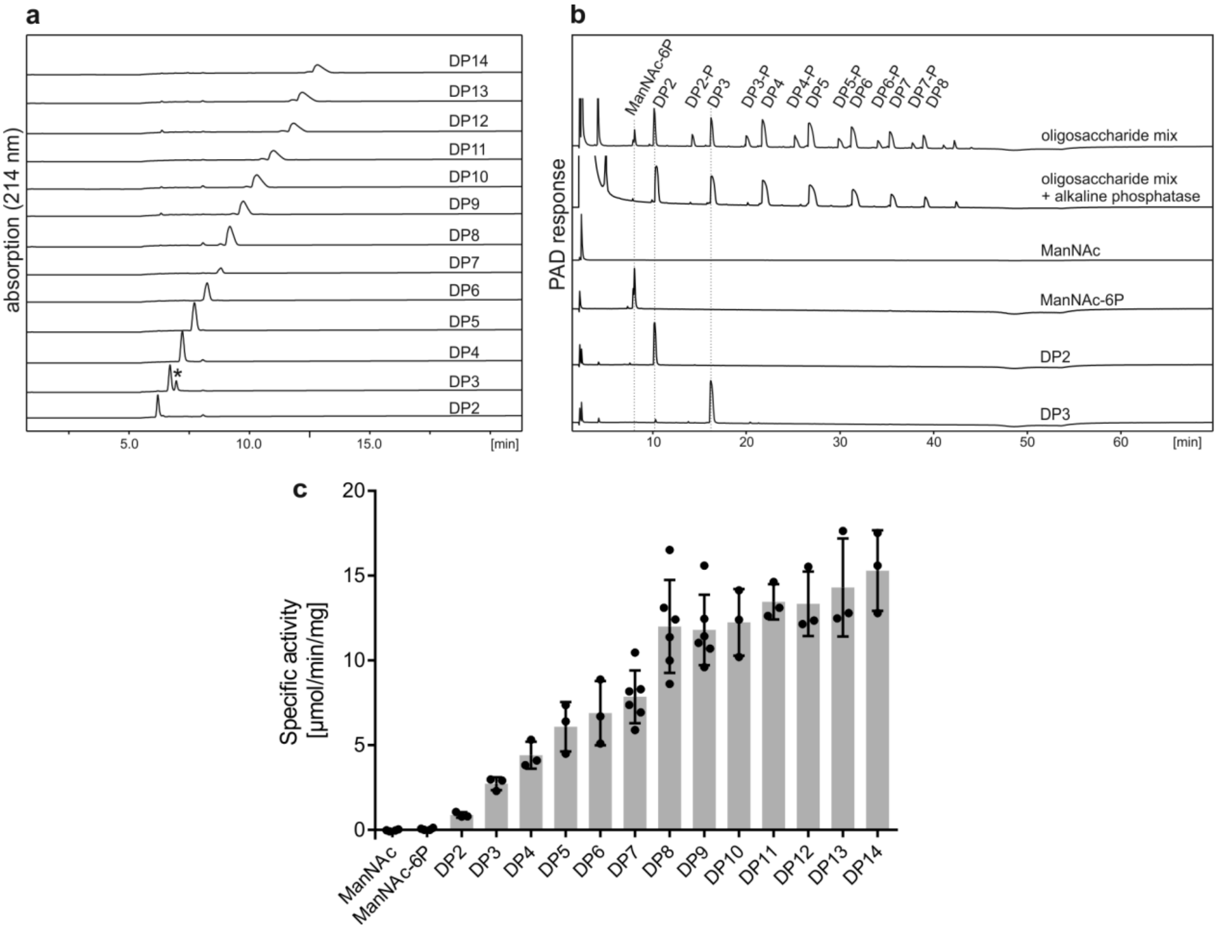

Supplementary Figure 14. Generation and testing of oligosaccharide acceptors with defined degree of polymerization. a, HPLC-AEC with UV detection (214 nm) showing dephosphorylated and purified oligosaccharides of defined DP. *indicates UV-active impurity in the DP3 sample which is not visible in HPAEC-PAD (see DP3 in (**b**)) and which is thus not of carbohydrate origin. **b,** HPAEC-PAD profiling as additional quality control for the DP3 containing fraction shown in (**a**). DP3 is highly pure and void of other phosphorylated and non-phosphorylated serogroup A oligosaccharides. Commercially available ManNAc and ManNAc-6P, DP2, as well as a serogroup A oligosaccharide mix (obtained through partial hydrolysis) before and after enzymatic removal of the non-reducing end phosphomonoester, are shown as standards. **c**, Spectrophotometric assay used to monitor CsaC activity in the presence of oligosaccharide acceptors with DP as indicated and commercially available ManNAc and ManNAc-6P. Error bars represent mean $\pm$ SD. The mean was calculated from n (shown as dots) independent experiments. DP7-9: n=6; ManNAc, ManNAc-6P: n=4; all other DPs: n=3. Source data are provided as a Source Data file.

Determination of the minimal acceptor length required by CsaC

Oligosaccharides of defined degree of polymerisation (DP) were prepared as previously described[14,15]. Briefly, enzymatically synthesized, non-*O*-acetylated serogroup A polymer was incubated in sodium acetate buffer (pH 4.8, 73°C, 6h) leading to the partial hydrolysis of anomeric phosphodiester bonds and yielding oligosaccharides that carry a phosphomonoester at the non-reducing end[16,17]. Since the NmA-CPS backbone, the natural substrate of CsaC *in vivo* (Fig. 1), has free non-reducing ends[15,18], the phosphomonoester was removed using calf intestinal alkaline phosphatase. The resulting mixture of oligosaccharides was separated by preparative anion exchange

chromatography yielding single oligosaccharide species of defined DP. The homogeneity of all oligosaccharides was corroborated by analytic HPLC-based anion exchange chromatography (HPLC-AEC) (**Supplementary Fig.** 14a). Due to an unexpected peak in the DP3 containing fraction (see * in **Supplementary Fig.** 14a), which is not uncommon during UV-detection at 214 nm and might result from process related impurities, High Performance Anion Exchange Chromatography coupled to Pulsed Amperometric Detection (HPAEC-PAD) was performed as additional quality control (**Supplementary Fig.** 14b). This technique is highly specific for the detection of carbohydrates and allows the discrimination of oligosaccharides with phosphorylated and non-phosphorylated non-reducing end. HPAEC-PAD clearly demonstrated that the DP3 containing fraction is highly homogenous, corroborating that the impurity does not results from unintentional co-purification of other phosphorylated or non-phosphorylated oligosaccharides.

In a next step, CsaC activity towards oligosaccharides of DP2-DP14 was monitored continuously using a spectrophotometric assay, in which free SH-groups of the CoA released after each *O*-acetyl transfer react with 5,5’-dithiobis(2-nitrobenzoic acid) to 5-thionitrobenzoic acid, which can be detected through absorption[19] (**Supplementary Fig.** 14c).

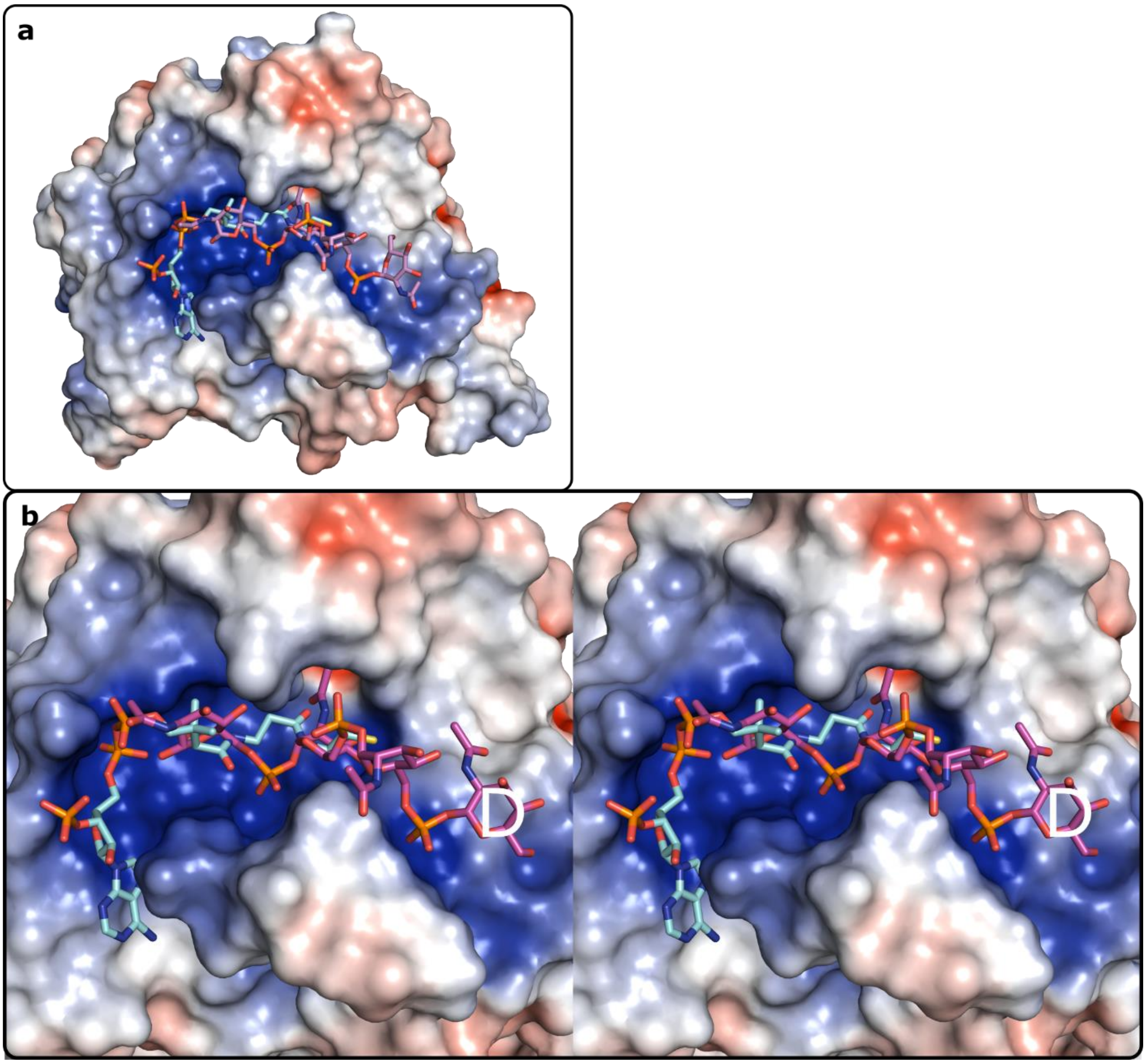

Supplementary Figure 15. Electrostatic surface potential (blue – positive, red – negative, white – neutral) representation of wild type CsaC in complex with CPS-DP4, showing overlay of CPS-DP4 (magenta) and CoA (cyan, from acetyl-CoA-soaked CsaC-H228A). **a**, Overview of the protomer, **b**, cross-eyed stereo close up of the binding sites and their overlap.

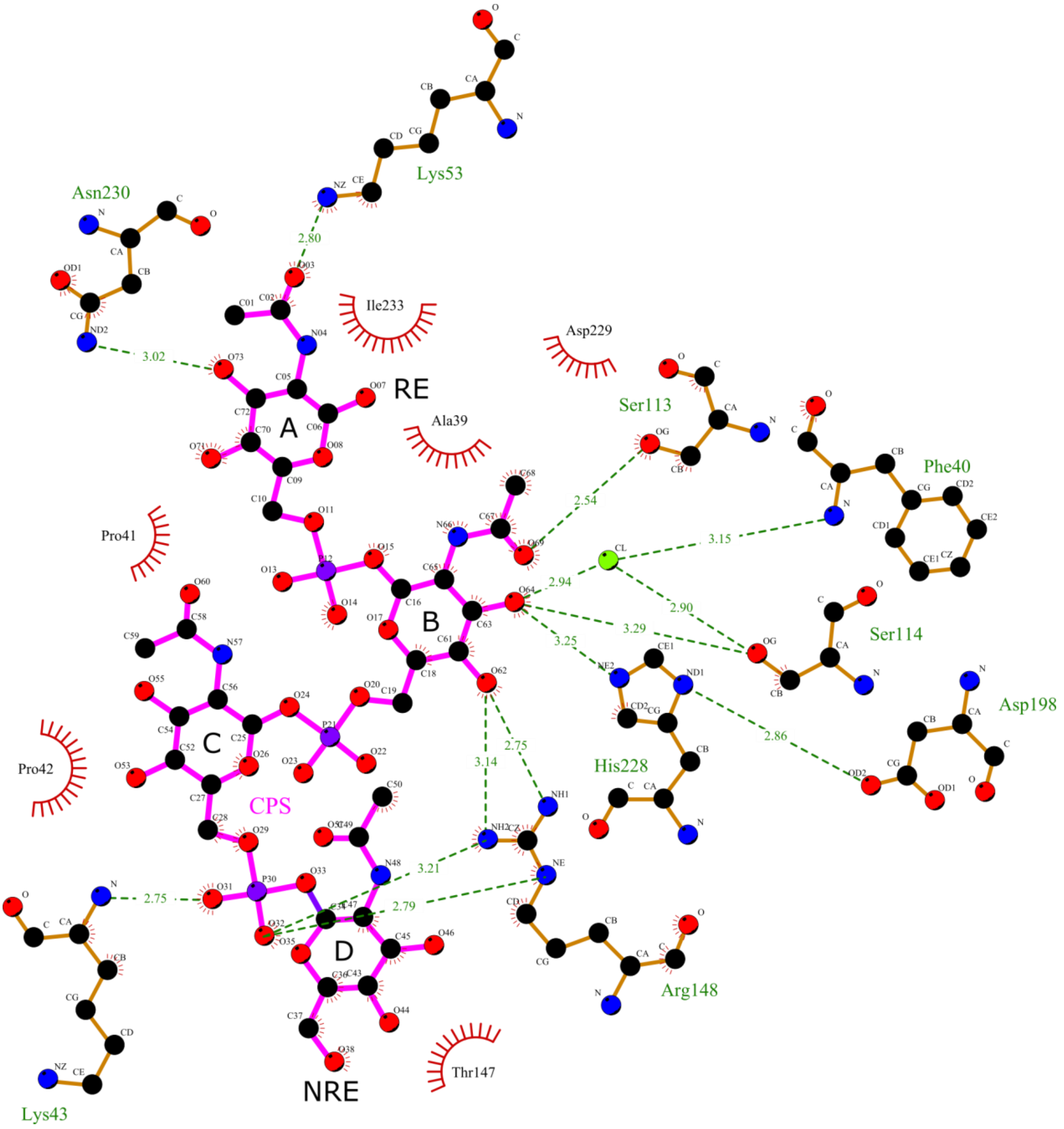

Supplementary Figure 16. Ligplot+[13] of CPS-DP4 (magenta) in the wild type CsaC structure chain A. Reducing (RE) and non-reducing (NRE) ends, as well as Ring A-D are labeled.

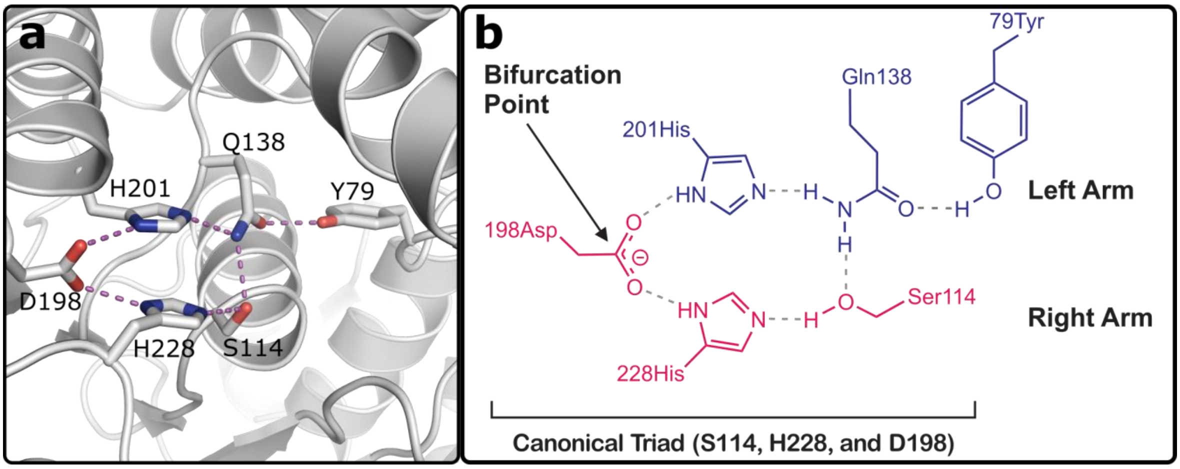

Supplementary Figure 17. **Bifurcated active site arrangement of CsaC. a**, Crystal structure depicting the active site of native wild type CsaC. **b**, Schematic view of the bifurcated active site arrangement with the canonical triad S114, H228 and D198 in red (right arm) and the additional residues H201, Q138 and Y79 (left arm) in blue.

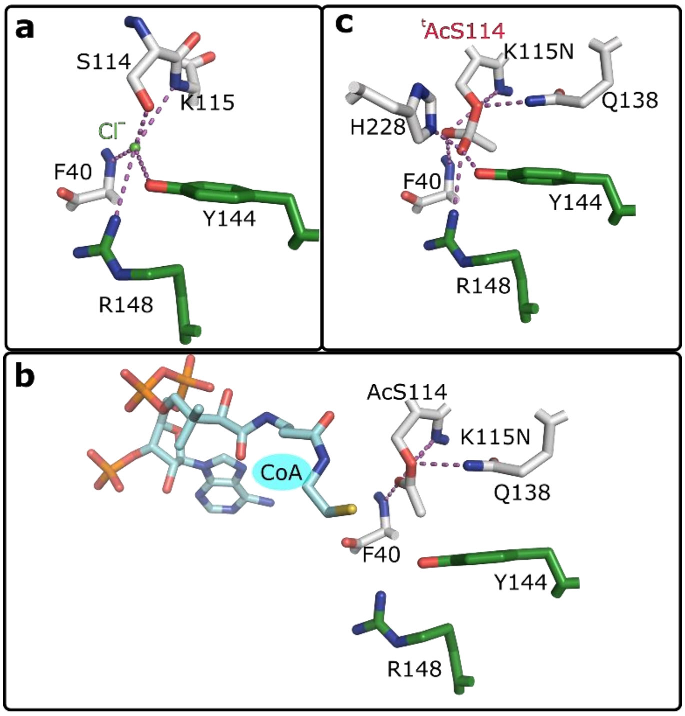

Supplementary Figure 18: CsaC oxyanion hole occupied by **a**, a chloride ion (Cl^-) in the native crystal, **b**, the acetyl-serine in the CsaC-H228A(-AcS114)-CoA complex and **c**, the predicted tetrahedral acetyl-serine intermediate. Hydrogen bond (dashed magenta lines) distances are ≤3.2 Å.

The catalytic machinery of ABH-fold enzymes is accomplished by an oxyanion hole, which binds carbonyl oxygens and stabilizes oxyanion-containing tetrahedral intermediates by interactions with two backbone amides[20,21]. In the CsaC-WT structures of the ligand-free form and the complex with CPS-DP4, the presumed oxyanion hole is occupied by a chloride ion (see also **Supplementary Fig.** 11b, 16) that may originate from the crystallization buffer. Based on its localization, we pinpointed F40-N and K115-N as the canonical structural components of the oxyanion hole. The chloride ion is further coordinated by the side chains of S114, Y144 and R148. In the structure of the CsaC-H228A(-AcS114)-CoA complex, the position of the chloride ion is occupied by the acetyl group of AcS114 (**Supplementary Fig.** 18b). Based on the CsaC-H228A(-AcS114) structure, a tetrahedral configuration of the carbonyl carbon of AcS114 was predicted as model of the transient tetrahedral oxyanion intermediate (**Supplementary Fig.** 18c).

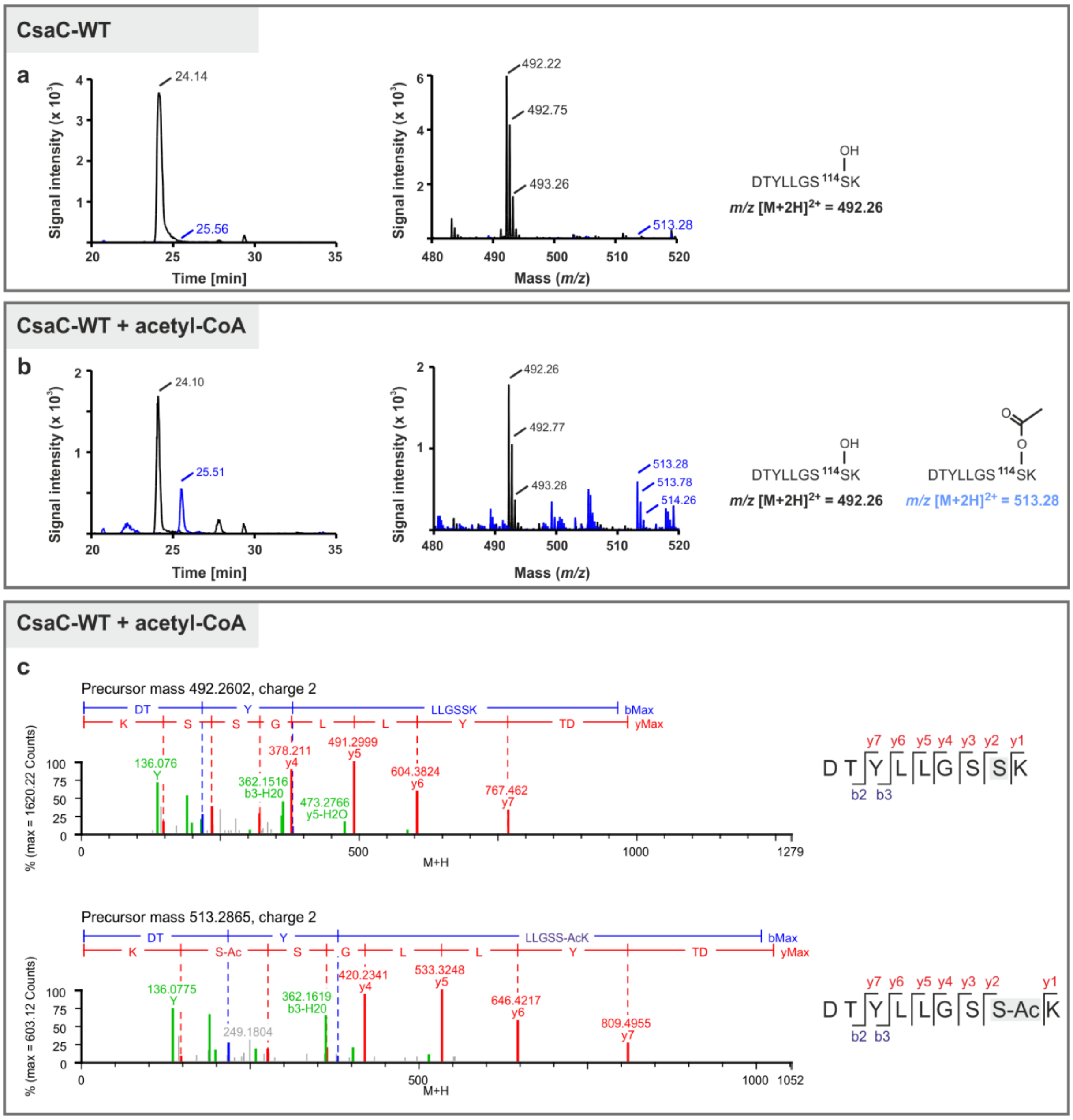

Supplementary Figure 19: MS analysis of the acetyl-enzyme intermediate formed by CsaC-WT. LC-ESI-MS analysis of purified CsaC-WT incubated **a**, without or **b**, with acetyl-CoA. After separation by SDS-PAGE and digest of the excised protein band by trypsin/Asp-N, each sample was analyzed by LC-ESI-MS. Theoretical m/z values are given in the right panel for the 2-fold charged peptides containing the active site serine S114. Spectra in the middle panel were acquired by accumulating scans within ± 0.1 min around the respective peak maxima in the extracted ion chromatograms of the given m/z values. **c**, Tandem MS spectra of the doubly charged S114-containing peptide in non-acetylated (m/z = 492.26) and acetylated (m/z = 513.28) form. MS/MS spectra were analysed using the program ProteinLynx Global Server (Version 2.1, Waters). The fragmentation pattern of the respective peptides are given on the right with the detected C-terminal (y-series) and N-terminal (b-series) ions indicated on top and below the peptide sequence, respectively.

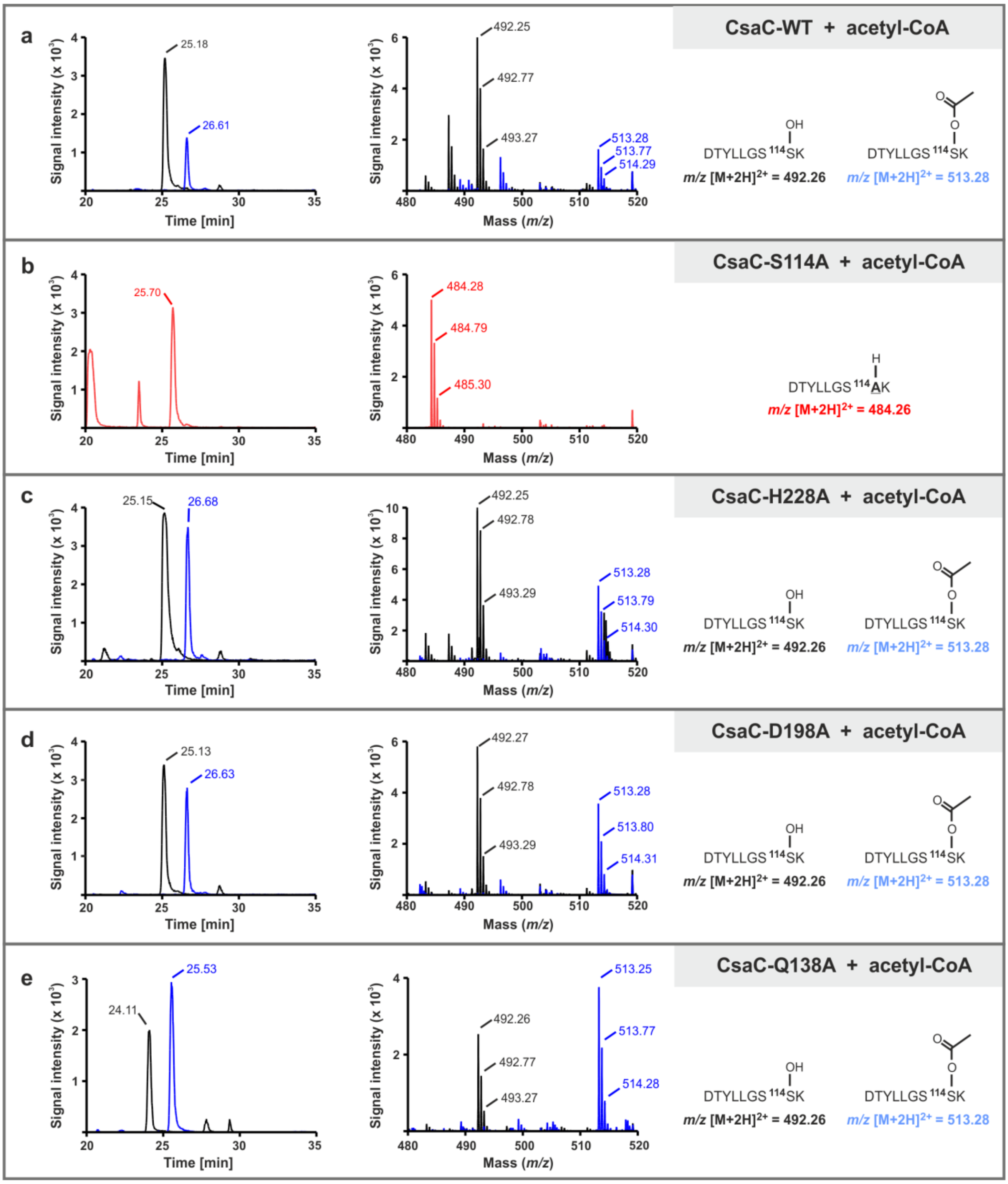

Supplementary Figure 20: LC-ESI-MS analysis of the acetyl-adduct in CsaC-WT and CsaC mutant forms. a, Purified CsaC-WT and **b-e**, indicated CsaC variants were incubated with acetyl-CoA and processed as described in the legend of **Supplementary Fig.** 19. Theoretical m/z values are given in the right panel for the 2-fold charged peptides containing the active site serine S114 (a, c-e) or the corresponding S114A mutation (b). Spectra in the middle panel were acquired by accumulating scans within ± 0.1 min around the respective peak maxima in the extracted ion chromatograms of the given m/z values. The peptide identity and the linkage of the acetyl group to S114 were confirmed by LC-ESI-MS/MS (**Supplementary Fig.** 21). The identity of the analyzed mutant variants was verified by detection of a peptide that harbors the alanine exchange (**Supplementary Fig.** 22).

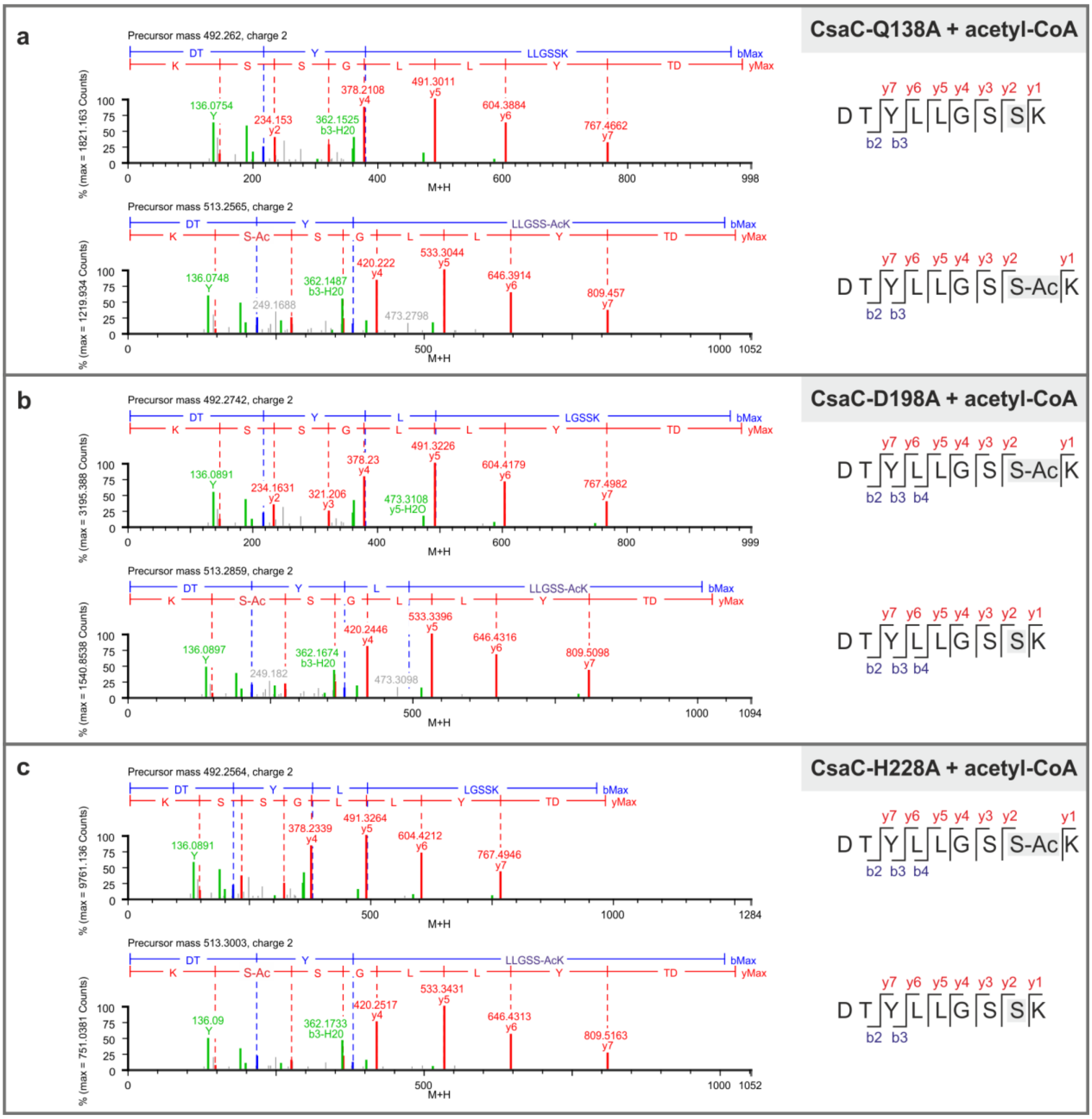

Supplementary Figure 21: LC-ESI-MS/MS analysis of acetyl-enzyme intermediates formed by CsaC variants. Tandem MS spectra of the doubly charged S114-containing peptides in non-acetylated (m/z = 492.26) and acetylated (m/z = 513.28) form obtained from the indicated CsaC variants incubated with acetyl-CoA. MS/MS spectra were analysed using the program ProteinLynx Global Server (Version 2.1, Waters). The fragmentation patterns of the respective peptides are given on the right with the detected C-terminal (y-series) and N-terminal (b-series) ions indicated on top and below the peptide sequence, respectively.

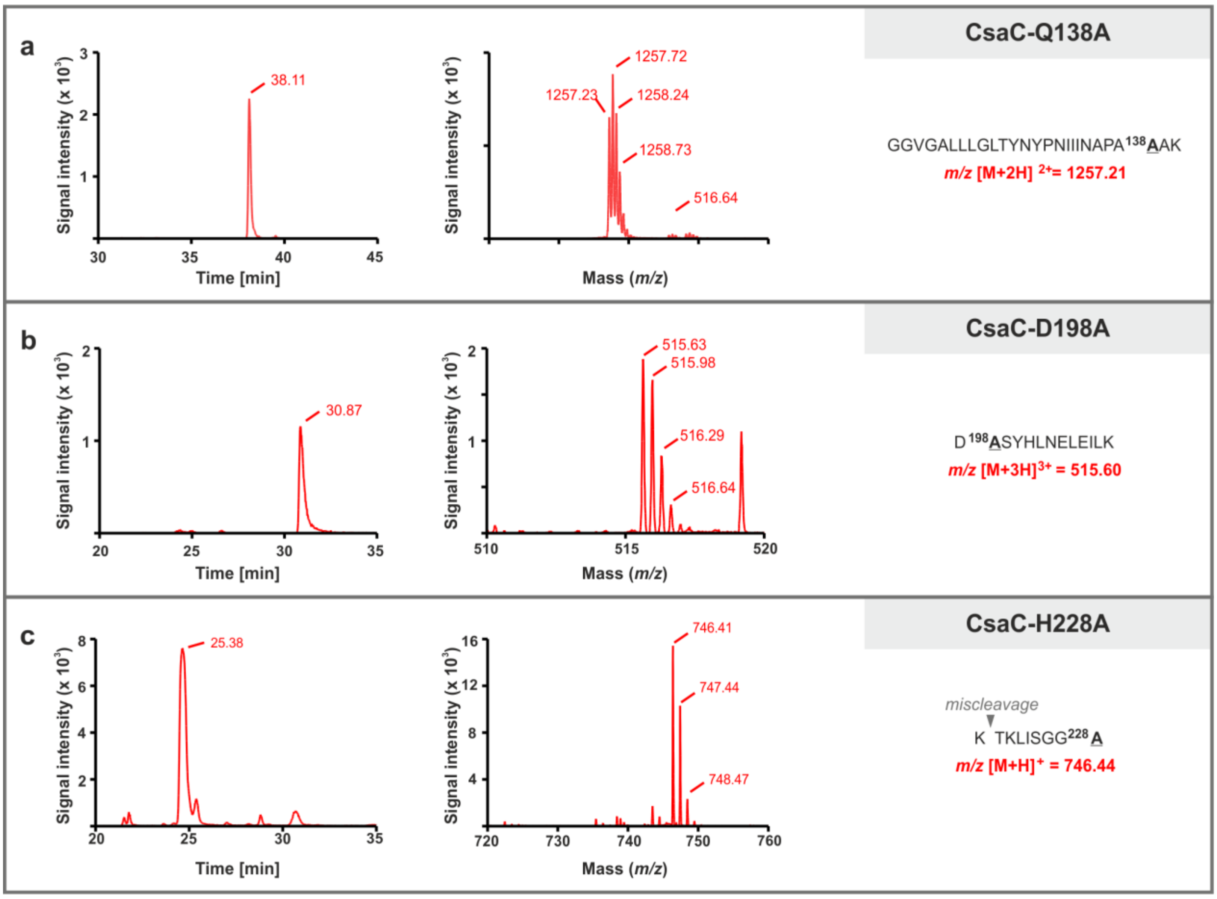

Supplementary Figure 22: Verification of CsaC mutant forms by LC-ESI-MS analysis. Alanine-exchanges in **a**, CsaC-Q138A, **b**, CsaC-D198A and **c**, CsaC-H228A were verified by analysis of peptides containing the respective exchange. Theoretical m/z values for these peptides are given in the right panel. Spectra in the middle panel were acquired by accumulating scans within ± 0.1 min around the respective peak maxima in the extracted ion chromatograms of the given m/z values.

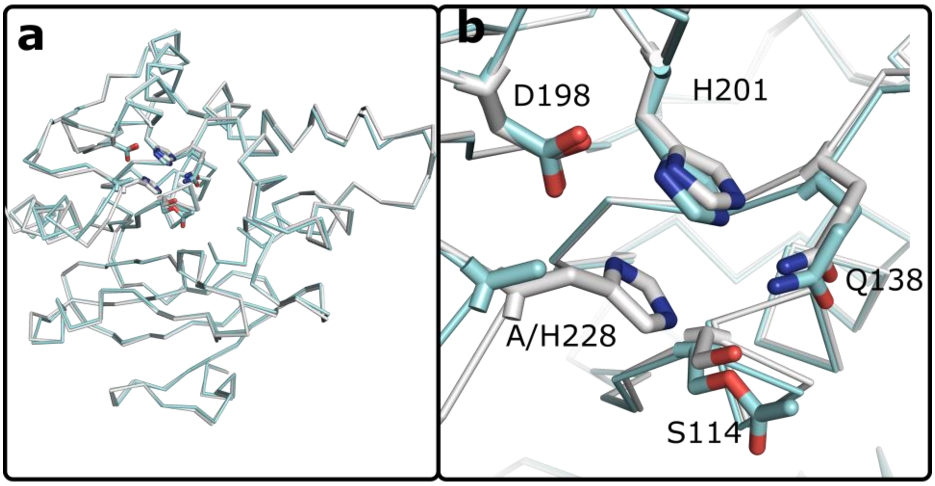

Supplementary Figure 23: Superposition of native wild type CsaC and CsaC-H228A(-AcS114)-CoA. a, C-alpha trace of secondary structure superposition of apo CsaC wild type and CsaC-H228A(-AcS114)-CoA (r.m.s.d. 0.384 Å). **b**, close up of the active sites.

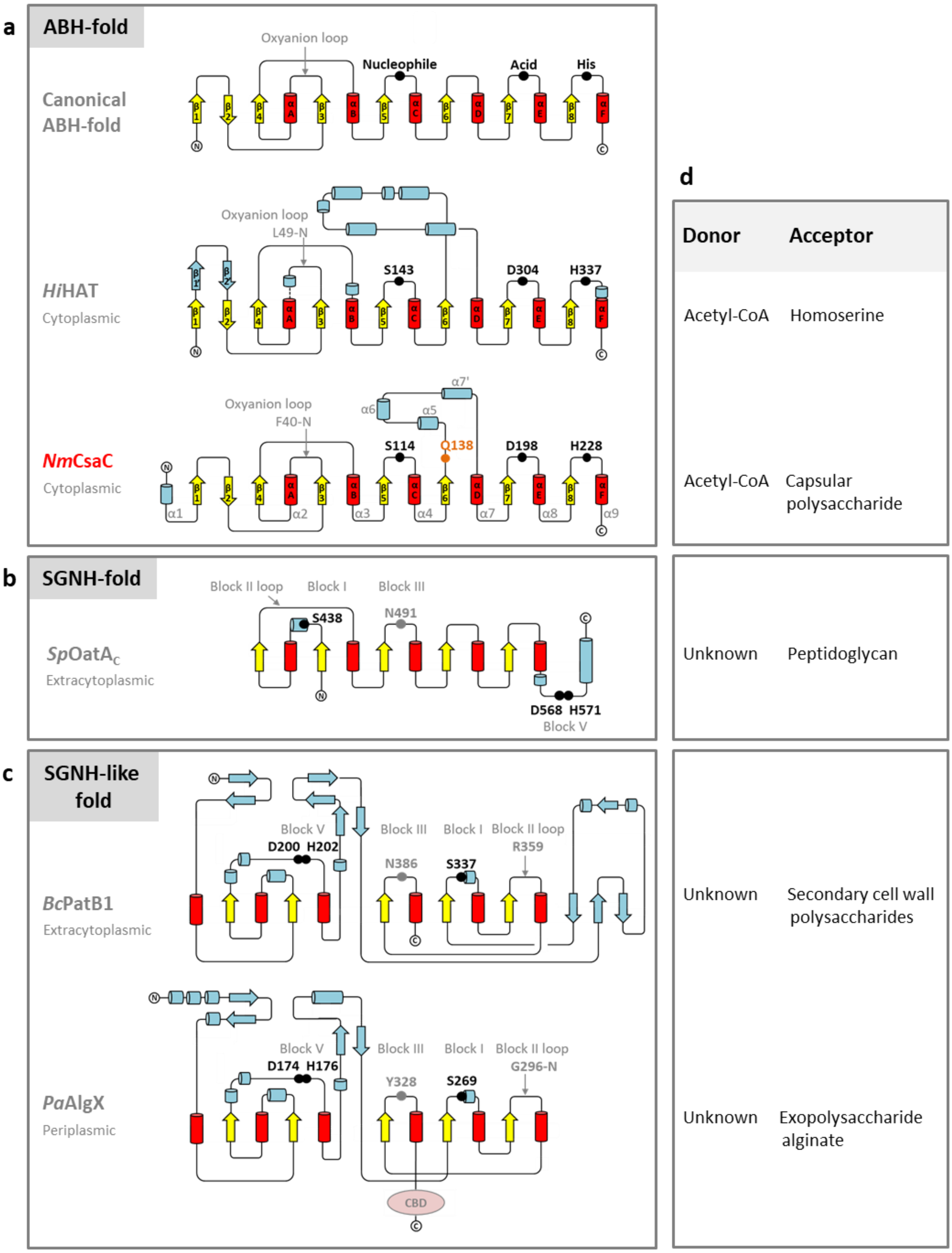

Supplementary Figure 24: Protein topology of structurally characterized *O*-acetyltransferases with Ser-His-Asp triad. Protein topology diagrams of *O*-acetyltransferases with a Ser-His-Asp triad were generated based on the following structures: *Hi*HAT, Homoserine *O*-acetyltransferase of *Haemophilus influenzae* (pdb code 2b61); *Nm*CsaC, capsule *O*-acetyltransferase of *Neisseria meningitidis* serogroup A (this study); *Sp*OatA$_C$, extracytoplasmic domain (residues 430-605) of the peptidoglycan *O*-acetyltransferase OatA of *Streptococcus pneumoniae* (pdb code 5ufy); *Bc*PatB1, secondary cell wall polysaccharide *O*-acetyltransferase of *Bacillus cereus* (pdb code 5v8e); *Pa*AlgX,

alginate *O*-acetyltransferase of *Pseudomonas aeruginosa* (pdb code 4knc). Helices and strands of the central fold are shown in red and yellow, respectively, and additional secondary structure elements are shown in light blue. The additional C-terminal carbohydrate binding domain (CBD) of *Pa*AlgX is shown as a pink sphere.

a, ABH-fold. The canaonical ABH-fold is composed of an 8-stranded mostly parallel β-sheet flanked by α-helices. The core fold serves as scaffold for the catalytic triad residues, which are placed on interconnecting loops and whose location in relation to the secondary structure topology defines the ABH family[21–23]. The catalytic nucleophile (Nu) is located between strand β5 and helix αC. It is part of the consensus sequence GxNuxG, which positions the nucleophile (serine or cysteine) at the tip of a very sharp turn, called the nucleophilic elbow. The catalytic histidine is located between strand β8 and helix αF, while the acid is placed between strand β7 and helix αE[21,22]. The oxyanion hole is formed by two main chain amides provided by the residue N-terminally adjacent to the catalytic serine and by a residue located in the oxyanion loop that interconnects strand β3 and helix αA. Structural information on ABH-fold transferases is largely limited to enzymes that are listed in the ESTHER database[23] as members of the homoserine transacetylase (HAT) subfamily[24,25]. As representative of this family, the topology of the HAT of *Haemophilus influenzae*[26] is shown in comparison to CsaC.

b, SGNH-fold. Members of the SGNH-fold family are characterized by conserved sequence blocks that contain the highly conserved residues S (the nucleophile in block I), G (in block II), N (in block III), and H (in block V), which gave the family its name[27]. The SGNH-fold enzymes harbor a Ser-His-Asp triad with the acid placed in a DxxH motif in block V that encompasses the catalytic histidine. The spatial orientation of the catalytic triad residues with respect to the overall α/β/α fold differs considerably between SGNH- and ABH-fold enzymes. In SGNH-fold enzymes, the triad residues align almost perpendicular to the central β-sheet and are placed on top of the N-terminal half of the central β-sheet, whereas in ABH-fold enzymes, the triad residues align almost parallel to the central β-sheet on top of the C-terminal half of the sheet[28]. Moreover, the nucleophilic serine of SGNH-fold enzymes is positioned in a GDS(L) motif close to the N-terminus and is part of a short α-helix. The canonical oxyanion whole of SGNH-fold enzymes is tri-, instead of di-residue-based as in the ABH-fold. In addition to two main chain amides (of the serine in block I and the glycine in block II), the oxyanion whole is accomplished by the side chain of the conserved asparagine in block III. All three residues are conserved in OatA$_C$ of *Staphylocoocus aureus*, whereas in OatA$_C$ of *Streptococcus pneumoniae*, the glycine in block II is exchanged by a serine (S461), whose main chain amide is atypically oriented away from the active site. The function of the conserved asparagine (N491 in *Sp*OatA$_C$) in stabilizing the oxyanion is exemplified in the *Sp*OatA$_C$ structure with methylsulfonyl adduct, which is covalently bound to the nucleophile S438 and represents a transition-state analogue[29] (**Supplementary Fig.** 25). Like SGNH-fold hydrolases, SGNH-fold *O*-acetyltransferases follow a ping-pong mechanism involving a covalent acetyl-enzyme intermediate as demonstrated for *Sp*OatA$_C$[30].

c, SGNH-like fold. Compared to canonical SGNH enzymes, SGNH-like esterases and *O*-acetyltransferases exhibit a circularly permuted order of the catalytic residues[31–33]. Similarly to SGNH enzymes, the nucleophile is located in a short α-helix and the triad residues align almost perpendicular to the central β-sheet. The DxxH motif of SGNH-fold enzymes is shortend to a DxH motif[31–33]. So far, two *O*-acetyltransferases with an SGNH-like fold have been crystallized, the alginate *O*-acetyltransferase AlgX of *Pseudomonas aeruginosa*[32] and the secondary cell wall polysaccharide *O*-acetyltransferases PatB of *Bacillus cereus*. In *Bc*PatB, the conserved glycine in block II is substituted by an arginine (R359), which atypically contributes *via* its side chain to the oxyanion whole, whereas the side chain of the conserved asparagine in block III (N386) is more distantly positioned[31]. In AlgX, the glycine in block II is conserved, but the asparagine in block III is substituted by a tyrosine (Y328)[31–33].

d, Differences in cellular localization and donor substrate usage. The ABH-fold *O*-acetyltransferases CsaC and HAT catalyze reactions in the cytoplasm and use acetyl-CoA as donor substrate[24–26]. The SGNH and SGNH-like cell wall *O*-acetyltransferases, by contrast, act in the extracytoplasmic space,

rely on the translocation of acetyl groups from the cytoplasm, and use an unidentified acetyl group donor. The proposed pathway for these enzymes includes (i) uptake of an acetyl group from a cytoplasmic acetyl donor (presumably acetyl-CoA), (ii) translocation of the acetyl group across the inner membrane through an N-terminal multipass transmembrane domain (OatA) or separate transmembrane protein (PatA and AlgI for PatB and AlgX, respectively), and (iii) acetyl group transfer onto a carbohydrate acceptor by the C-terminal SGNH-fold domain (OatA) or the separate SGNH-like *O*-acetyltransferase (PatB, AlgX)[29,31,32,34]. As the nature of the acetyl group donor of the SGNH/SGNH-like *O*-acetyltransferases is not known, artificial donor substrates (*p*-nitrophenyl-acetate or 4-methylumbelliferyl-acetate) have been used to study the enzymatic function of these enzymes.

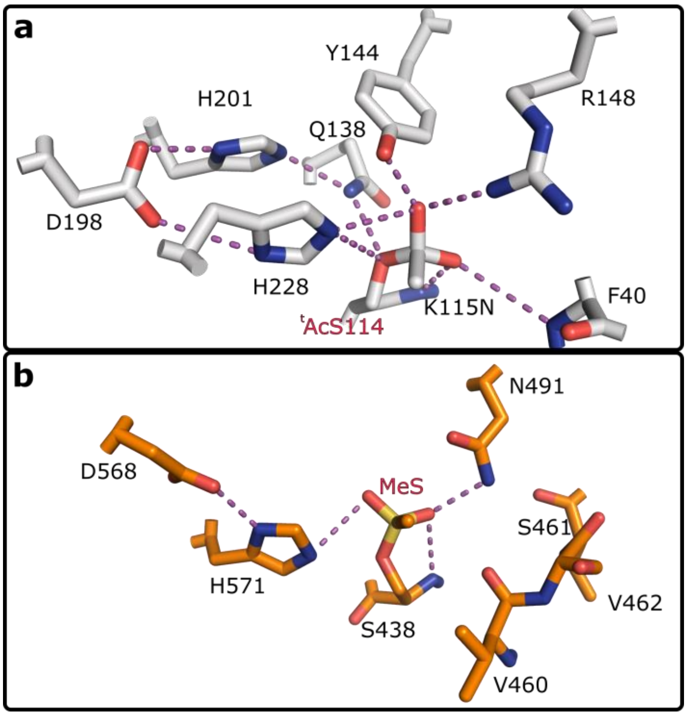

Supplementary Figure 25: Comparison of active site residues of **a**, proposed theoretical intermediate structure of CsaC and **b**, SGNH-fold representative *Sp*OatA$_C$ (pdb id: 5ug1). CsaC intermediate structure was manually built based on the wild type CsaC structure (pdb id: 6YUV) and energy minimized in PyMOL. The oxyanion hole of CsaC is formed by K115-N and F40-N, whereas in *Sp*OatA$_C$, S438-N and N491 accomplish this function. The unique glutamine residue Q138 of CsaC is not part of the oxyanion hole and an equivalent residue is missing in SGNH- and SGNH-like enzymes.

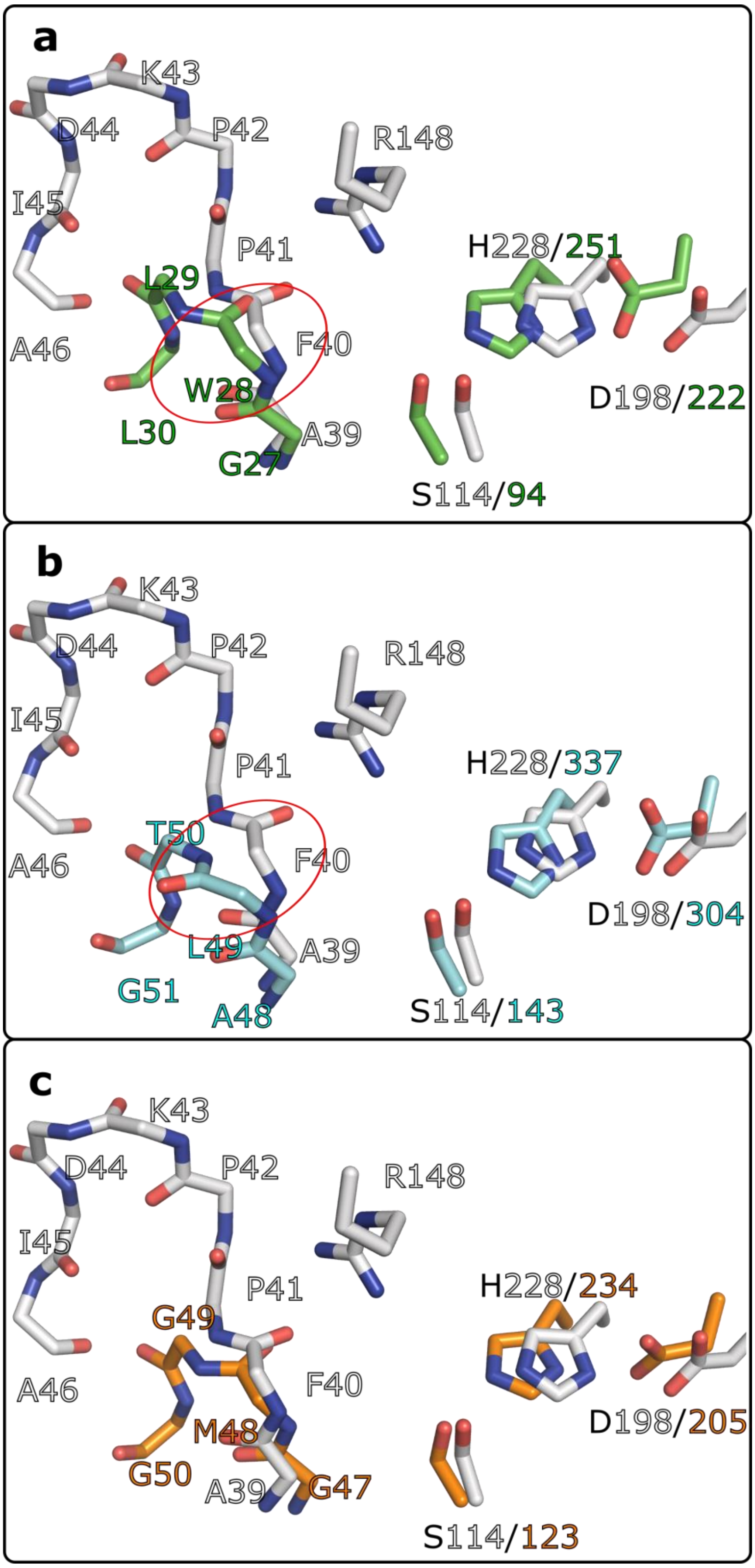

Supplementary Figure 26: Main chain conformation of the oxyanion loop. Secondary structure superposition of wild type CsaC (grey) with **a**, esterase PFE (type I β-turn, green, pdb: 1va4), **b**, acetyl transferase *Hi*HAT (type II β-turn, cyan, pdb: 2b61), and **c**, closest DALI search result esterase A (type I β-turn, orange, pdb: 4rot, DALI z-score = 18.3).

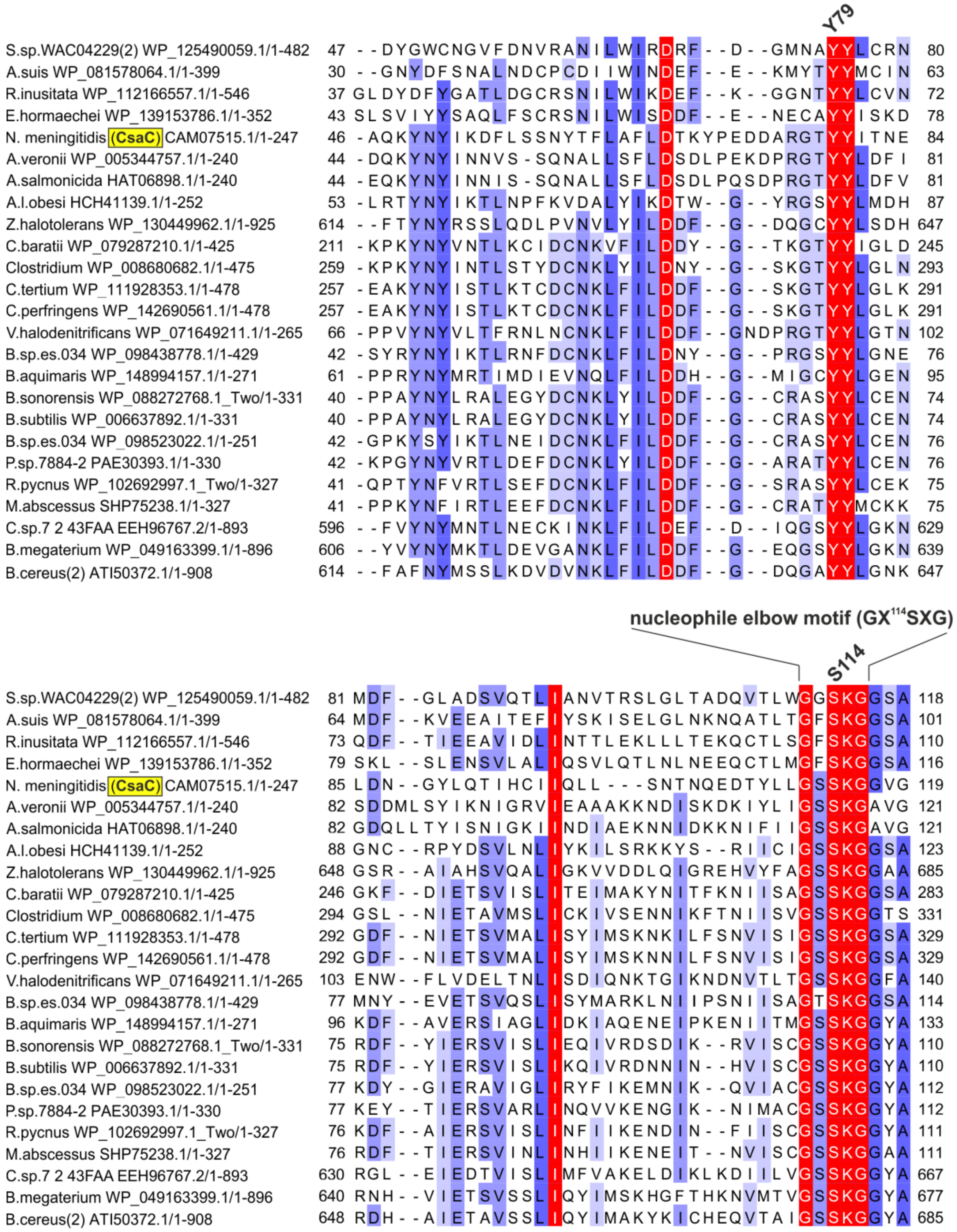

Supplementary Figure 27 Part 1: Multiple-sequence alignement.

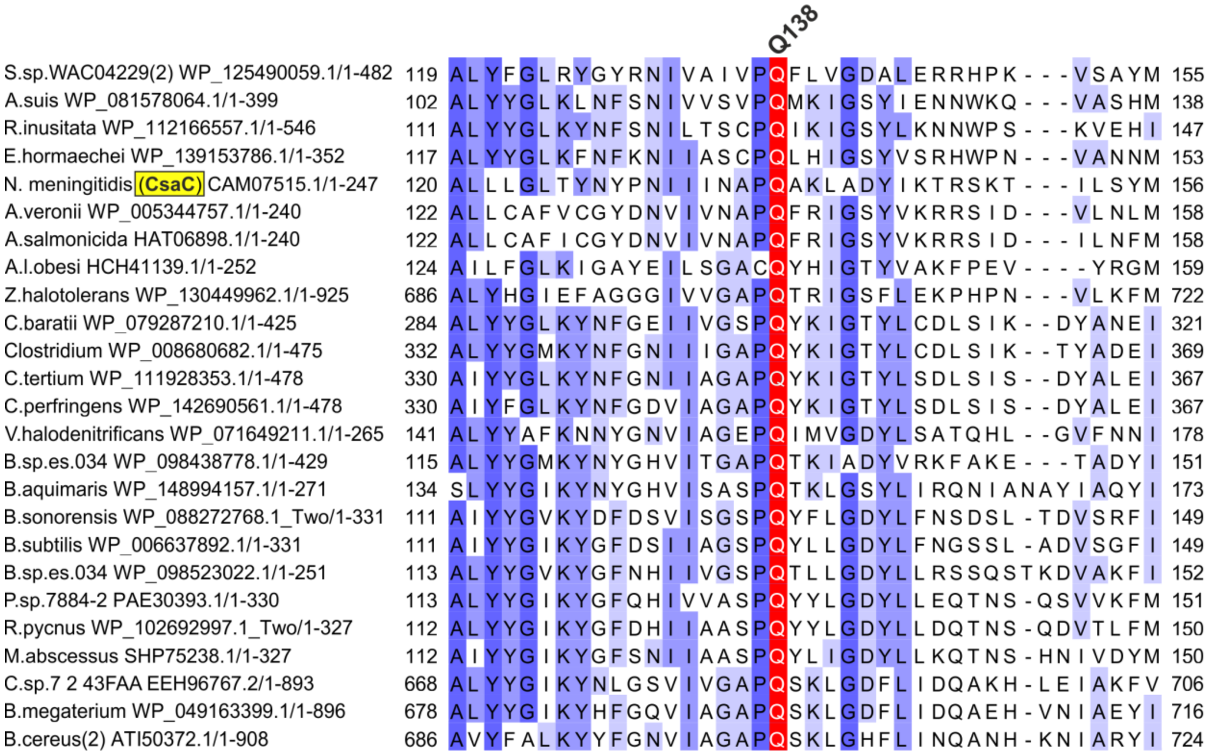

Supplementary Figure 27 part 2: Multiple-sequence alignment. Selected part of a multiple-sequence alignment (a complete alignment is shown in **Supplementary Data 1**) of putative CsaC homologs identified through BLAST[35] searches using the Blastp algorithm and the CsaC amino acid sequence as query. Species, accession number and sequence coverage for each database entry, as well as the first and the last amino acid of each aligned amino acid stretch, are indicated. Identical amino acids are highlighted from light to dark blue with increasing conservation. The entire alignment comprised only nine amino acid positions with 100% identity (highlighted in red). These include the catalytic triad serine S114 placed in a nucleophile elbow motif, as well as Y79 and Q138, which form the ‘extra’ arm of the unconventional, bifurcated active site arrangement of CsaC (**Supplementary Fig.** 17).

Supplementary Notes

Supplementary Note 1

Quantification based on ^{1}H NMR

Previous studies quantified the *O*-acetylation pattern of the NmA-CPS according to a method published by Lemercinier and Jones (1996)[3]. This method is based on the H2 signals of a one-dimensional ^{1}H spectrum and distinguishes 3OAc-ManNAc (corresponding to spin system *1* from this study, see Fig. 2), 3OAc-ManNAc linked to 4OAc-ManNAc at its reducing end (corresponding to *2* from this study), 4OAc-ManNAc (corresponding to *3* + *4* from this study) and nonOAc-ManNAc (corresponding to *5* + *6* + *7* + *8* from this study) (see **Supplementary Note 1 Figure** and Fig. 2).

Application of this method for the analysis of the material shown in Fig. 2d revealed that 47.8% of the entire H2 signals result from 3OAc-ManNAc (*1*), 9.0% from 3OAc-ManNAc that is followed by 4OAc-ManNAc (*2*, indicated by an asterisk in the **Supplementary Note 1 Figure**), 13.9% from 4OAc-ManNAc (*3* + *4*) and 29.3% from nonOAc-ManNAc (*5* + *6* + *7* + *8*, **Supplementary Note 1 Figure**). Note that the material analyzed in Fig. 2b and Fig.2d/**Supplementary Note 1 Figure** result from two different *O*-acetyl migration experiments, leading to slightly different *O*-acetylation levels.

Here, we performed a complete 2D assignment of the *O*-acetylated and non-*O*-acetylated serogroup A polymer (Fig. 2 and **Supplementary Figs.** 4-8). This allowed the identification of nine distinct spin systems (Fig. 2d), of which eight (*1*-*8*) contribute to the previously assigned H2 chemical shift pattern (**Supplementary Note 1 Figure**, Fig. 2d) and one (H2 of *9*, 4.345 ppm) overlaps with H3 of spin system *3* (Fig. 2d). Our assignment offers new possibilities for quantifying the various structural motifs present in the *O*-acetylated CPS and allows an estimation of the relative distribution of 4OAc-ManNAc within the polymer as outlined below.

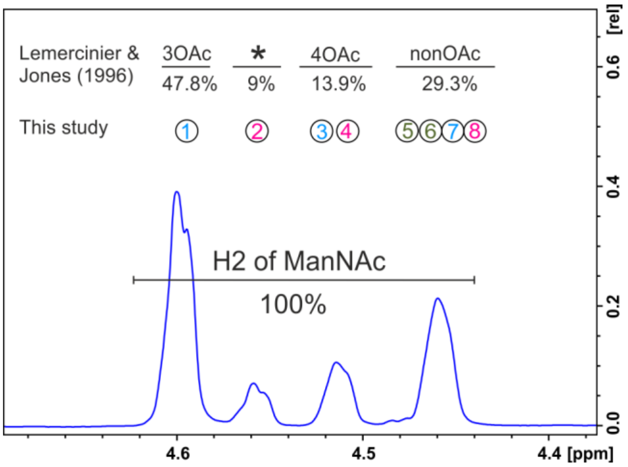

Supplementary Note 1 Figure. H2 chemical shift pattern of *O*-acetylated NmA-CPS. Lemercinier and Jones (1996)[3] assigned four spin systems to the H2 chemical shift pattern observed in ^{1}H NMR spectra of *O*-acetylated NmA-CPS. We showed in this study that the observed H2 signal pattern is more complex and results from eight overlapping spin systems (see. Fig. 2). * internal 3OAc-ManNAc linked to 4OAc-ManNAc at its reducing end as suggested in Lemercinier and Jones (1996)[3].

Quantification based on ^{31}P NMR

The knowledge gained from the *O*-acetyl group migration experiment and the 2D NMR analysis allowed the assignment of all phosphodiester peaks observed in the one-dimensional ^{31}P NMR spectrum (Fig. 2e). The ^{31}P signals can now be used to quantify the percentage of (i) 3OAc-ManNAc linked to 4OAc-ManNAc (7.9%, *2*, -5.88 ppm, Fig. 2e), (ii) the sum of 4OAc-ManNAc and nonOAc-ManNAc that are linked to 4OAc-ManNAc (5.3%, *4* + *8*, ~ -5.73 ppm, Fig. 2e), and (iii) the sum of all residues connected to 4OAc-ManNAc at their reducing ends (13.2%, *2* + *4* + *8*, ~ -5.73 ppm + -5.88 ppm, Fig. 2e). It is worth noting that the latter corresponds nicely to the amount of all 4O-acetylated units calculated from the ^{1}H spectrum (13.9%, *3* + *4*, **Supplementary Note 1 Figure**). Also for spin system *2*, the populations calculated from ^{1}H and ^{31}P spectra are in fairly good agreement (9.0% vs. 7.9%). As previously published[16], spin system *6* can be quantified from the well isolated phosphomonoester peak located at the non-reducing end of the oligosaccharides (1.0 ppm, 2.4%).

Information about the correlation between the anomeric signals and the phosphodiester signals were shared with collaborators from industry and helped to develop a method that facilitates the estimation of *O*-acetyl levels of serogroup A polymer in a quadrivalent vaccine formulation based on the chemical shift of H1[36]. Although suitable for an estimation of the *O*-acetyl content in a polysaccharide mixture, the method proved to be less precise than the previously published quantification based on H2[3], due to overlapping of nonOAc-ManNAc and OAc-ManNAc H1 peaks.

Quantification based on 2D ^{1}H-^{1}H TOCSY

Since neither of the one-dimensional spectra (^{1}H and ^{31}P) allowed the quantification of the individual spin systems *3*, *4*, *7*, *8* and *9*, we present here an estimation of the relative populations of eight out of nine spin systems, which can be gained through comparison of the H2-H5 correlations in the ^{1}H-^{1}H TOCSY spectrum (Fig. 2d). This is possible because the topologies of the spin systems are identical and thus the scalar couplings are as well. Therefore, comparing always the same correlation between proton H2 and proton H5 should reflect their populations. This analysis reveals the following populations for the material shown in Fig. 2d: 46.8±1% *1*, 8.2%±1% *2*, 11.7%±1% *3*, 1.7%±1% *4*, 1.3%±1% *6*, 25.3%±1% *7*, 3.2%±1% *8* and 1.9%±1% *9*.

We are aware that the presented relative populations have to be considered with care, because the TOCSY is not a particular quantitative experiment and because some signals overlap slightly with others (see H2-H5 correlations in Fig. 2d). The provided values represent an estimation of the relative populations of the spin systems rather than a precise quantification. However, it is worth noting that the obtained percentages agree with the values obtained from the 1D spectra, even for the least populated spin systems: (i) The percentages of 4O-acetylated units (11.7% (*3*)+ 1.7% (*4*)) calculated from the ^{1}H-^{1}H TOCSY and ^{1}H spectrum (**Supplementary Note 1 Figure**) are 13.4% and 13.9%,

respectively; (ii) The sum of *4* + *8* calculated from the ^{31}P spectrum and ^{1}H-^{1}H TOCSY is 5.3% and 4.9%, respectively.

Notably, the ^{1}H-^{1}H TOCSY-based quantification allows for the first time an overview of the abundances of different moieties connected to O6 of 4OAc-ManNAc: Setting all 3OAc-ManNAc signals (*1* + *2*) to 100% allows the calculation of the percentage of 3OAc-ManNAc residues that are linked to O6 of 4OAc-ManNAc (*2*) to be 14.9% of the entire 3OAc-ManNAc population. In analogy, setting all 4OAc-ManNAc signals (*3* + *4*) to 100% reveals that 12.7% of all 4OAc-ManNAc are linked to O6 of 4OAc-ManNAc (*4*); and setting all internal nonOAc-ManNAc signals to 100% (*7* + *8*) indicates that 11.2% of those nonOAc-ManNAc are linked to O6 of 4OAc-ManNAc. Judging from this estimation, there appears to be no obvious bias for a particular *O*-acetylation status of the residue connected to O6 of 4OAc-ManNAc. Consequently, this finding suggests that *O*-acetyl migration is not triggered by the adjacent non-reducing end neighbor.

Supplementary References

1. Coxon, B. Deuterium isotope effects in carbohydrates revisited. Cryoprobe studies of the anomerization and NH to ND deuterium isotope induced 13C NMR chemical shifts of acetamidodeoxy and aminodeoxy sugars. *Carbohydr. Res.* **340**, 1714–21 (2005).

2. Pan, Y., Ayani, T., Nadas, J., Wen, S. & Guo, Z. Accessibility of N-acyl-D-mannosamines to N-acetyl-D-neuraminic acid aldolase. *Carbohydr. Res.* **339**, 2091–100 (2004).

3. Lemercinier, X. & Jones, C. Full 1H NMR assignment and detailed O-acetylation patterns of capsular polysaccharides from Neisseria meningitidis used in vaccine production. *Carbohydr. Res.* **296**, 83–96 (1996).

4. Bardotti, A. *et al.* Physicochemical characterisation of glycoconjugate vaccines for prevention of meningococcal diseases. *Vaccine* **26**, 2284–96 (2008).

5. Lupisan, S. *et al.* Meningococcal polysaccharide A O-acetylation levels do not impact the immunogenicity of the quadrivalent meningococcal tetanus toxoid conjugate vaccine: results from a randomized, controlled phase III study of healthy adults aged 18 to 25 years. *Clin. Vaccine Immunol.* **20**, 1499–507 (2013).

6. Perry, M. B., Altman, E., Brisson, J. R., Beynon, L. M. & Richards, J. C. Structural characteristics of the antigenic capsular polysaccharides and lipopolysaccharides involved in the serological classification of Actinobacillus (Haemophilus) pleuropneumoniae strains. *Serodiagn. Immunother. Infect. Dis.* **4**, 299–308 (1990).

7. Berti, F., De Ricco, R. & Rappuoli, R. Role of O-Acetylation in the Immunogenicity of Bacterial Polysaccharide Vaccines. *Molecules* **23**, 1–10 (2018).

8. Gasmi, G. *et al.* 31P NMR characterization of terminal phosphates induced on DNA by the artificial nuclease ‘Mn-TMPyP/KHSO5’ in comparison with DNases I and II. *Nucleic Acids Res.* **19**, 2835–9 (1991).

9. Schuck, P. Size-distribution analysis of macromolecules by sedimentation velocity ultracentrifugation and lamm equation modeling. *Biophys. J.* **78**, 1606–19 (2000).

10. Zhao, H., Brautigam, C. A., Ghirlando, R. & Schuck, P. Overview of current methods in sedimentation velocity and sedimentation equilibrium analytical ultracentrifugation. *Curr. Protoc. protein Sci.* **Chapter 20**, Unit20.12 (2013).

11. Brookes, E., Demeler, B. & Rocco, M. Developments in the US-SOMO Bead Modeling Suite: New Features in the Direct Residue-to-Bead Method, Improved Grid Routines, and Influence of Accessible Surface Area Screening. *Macromol. Biosci.* **10**, 746–753 (2010).

12. Rocco, M. & Byron, O. Computing translational diffusion and sedimentation coefficients: an evaluation of experimental data and programs. *Eur. Biophys. J.* **44**, 417–431 (2015).

13. Laskowski, R. A. & Swindells, M. B. LigPlot+: Multiple ligand-protein interaction diagrams for drug discovery. *J. Chem. Inf. Model.* **51**, 2778–2786 (2011).

14. Fiebig, T. *et al.* Efficient solid-phase synthesis of meningococcal capsular oligosaccharides enables simple and fast chemoenzymatic vaccine production. *J. Biol. Chem.* **293**, 953–962 (2018).

15. Fiebig, T. *et al.* Molecular cloning and functional characterization of components of the capsule biosynthesis complex of Neisseria meningitidis serogroup A: toward in vitro vaccine production. *J. Biol. Chem.* **289**, 19395–407 (2014).

16. Berti, F. *et al.* Relative stability of meningococcal serogroup A and X polysaccharides. *Vaccine* **30**, 6409–15 (2012).

17. Ricci, S., Bardotti, A., D'Ascenzi, S. & Ravenscroft, N. Development of a new method for the quantitative analysis of the extracellular polysaccharide of Neisseria meningitidis serogroup A by use of high-performance anion-exchange chromatography with pulsed-amperometric detection. *Vaccine* **19**, 1989–97 (2001).

18. Willis, L. M. & Whitfield, C. Structure, biosynthesis, and function of bacterial capsular polysaccharides synthesized by ABC transporter-dependent pathways. *Carbohydr. Res.* **378**, 35–44 (2013).

19. Bergfeld, A. K. *et al.* The polysialic acid-specific O-Acetyltransferase OatC from Neisseria meningitidis serogroup C evolved apart from other bacterial sialate O-Acetyltransferases. *J. Biol. Chem.* **284**, 6–16 (2009).

20. Rauwerdink, A. & Kazlauskas, R. J. How the Same Core Catalytic Machinery Catalyzes 17 Different Reactions: the Serine-Histidine-Aspartate Catalytic Triad of α/β-Hydrolase Fold Enzymes. *ACS Catal.* **5**, 6153–6176 (2015).

21. Holmquist, M. Alpha/Beta-hydrolase fold enzymes: structures, functions and mechanisms. *Curr. Protein Pept. Sci.* **1**, 209–35 (2000).

22. Ollis, D. L. *et al.* The alpha/beta hydrolase fold. *Protein Eng.* **5**, 197–211 (1992).

23. Lenfant, N. *et al.* ESTHER, the database of the α/β-hydrolase fold superfamily of proteins: tools to explore diversity of functions. *Nucleic Acids Res.* **41**, D423-9 (2013).

24. Lejon, S., Ellis, J. & Valegård, K. The last step in cephalosporin C formation revealed: crystal structures of deacetylcephalosporin C acetyltransferase from Acremonium chrysogenum in complexes with reaction intermediates. *J. Mol. Biol.* **377**, 935–44 (2008).

25. Oda, K., Matoba, Y., Kumagai, T., Noda, M. & Sugiyama, M. Crystallographic study to determine the substrate specificity of an L-serine-acetylating enzyme found in the D-cycloserine biosynthetic pathway. *J. Bacteriol.* **195**, 1741–9 (2013).

26. Mirza, I. A., Nazi, I., Korczynska, M., Wright, G. D. & Berghuis, A. M. Crystal structure of homoserine transacetylase from Haemophilus influenzae reveals a new family of alpha/beta-hydrolases. *Biochemistry* **44**, 15768–73 (2005).

27. Upton, C. & Buckley, J. T. A new family of lipolytic enzymes? *Trends Biochem. Sci.* **20**, 178–179 (1995).

28. Mølgaard, A., Kauppinen, S. & Larsen, S. Rhamnogalacturonan acetylesterase elucidates the structure and function of a new family of hydrolases. *Structure* **8**, 373–383 (2000).

29. Sychantha, D. *et al.* In vitro characterization of the antivirulence target of Gram-positive pathogens, peptidoglycan O-acetyltransferase A (OatA). *PLoS Pathog.* **13**, 1–26 (2017).

30. Sychantha, D. & Clarke, A. J. Peptidoglycan Modification by the Catalytic Domain of Streptococcus pneumoniae OatA Follows a Ping-Pong Bi-Bi Mechanism of Action. *Biochemistry* **57**, 2394–2401 (2018).

31. Sychantha, D. *et al.* PatB1 is an O-acetyltransferase that decorates secondary cell wall polysaccharides. *Nat. Chem. Biol.* **14**, 79–85 (2018).

32. Riley, L. M. *et al.* Structural and functional characterization of Pseudomonas aeruginosa AlgX: role of AlgX in alginate acetylation. *J. Biol. Chem.* **288**, 22299–314 (2013).

33. Baker, P. *et al.* P. aeruginosa SGNH Hydrolase-Like Proteins AlgJ and AlgX Have Similar Topology but Separate and Distinct Roles in Alginate Acetylation. *PLoS Pathog.* **10**, (2014).

34. Jones, C. S., Sychantha, D., Howell, P. L. & Clarke, A. J. Structural basis for the O-acetyltransferase function of the extracytoplasmic domain of OatA from Staphylococcus aureus. *J. Biol. Chem.* **295**, 8204–8213 (2020).

35. Boratyn, G. M. *et al.* BLAST: a more efficient report with usability improvements. *Nucleic Acids Res.* **41**, W29-33 (2013).

36. Martini, S. *et al.* Correction to 'NMR Assays for Estimating the O-Acetyl Content of Meningococcal Polysaccharide Serogroup A in Quadrivalent Conjugate Vaccine Formulation'. *ACS omega* **4**, 15771 (2019).
