# Supplementary material for: Structural and mechanistic basis of capsule O-acetylation in Neisseria meningitidis serogroup A
Source: Nat Commun. 2020 Sep 18;11:4723. doi: 10.1038/s41467-020-18464-y (PMC7501274; doi:10.1038/s41467-020-18464-y)
Supplement: Supplementary file 2 — Description of Additional Supplementary Files [file 41467_2020_18464_MOESM2_ESM.pdf]

Description of Additional Supplementary Files

Title: Supplementary Data 1

Description: Multiple-sequence alignment. Multiple-sequence alignment of putative CsaC homologs identified through BLAST searches using the Blastp algorithm and the CsaC amino acid sequence as query. Accession number and species for each database entry are indicated. Identical amino acids are highlighted in yellow, highly conserved residues of the catalytic triad are highlighted in green.
