# Supplementary material for: Structural and mechanistic basis of capsule O-acetylation in Neisseria meningitidis serogroup A
Source: Nat Commun. 2020 Sep 18;11:4723. doi: 10.1038/s41467-020-18464-y (PMC7501274; doi:10.1038/s41467-020-18464-y)
Supplement: Supplementary file 4 — Reporting Summary [file 41467_2020_18464_MOESM4_ESM.pdf]

nature research

Corresponding author(s): Martina Mühlenhoff & Timm Fiebig

Last updated by author(s): Aug 14, 2020

Reporting Summary

Nature Research wishes to improve the reproducibility of the work that we publish. This form provides structure for consistency and transparency in reporting. For further information on Nature Research policies, see our Editorial Policies and the Editorial Policy Checklist.

Statistics

For all statistical analyses, confirm that the following items are present in the figure legend, table legend, main text, or Methods section.

n/a	Confirmed	
☐	☒	The exact sample size (*n*) for each experimental group/condition, given as a discrete number and unit of measurement
☐	☒	A statement on whether measurements were taken from distinct samples or whether the same sample was measured repeatedly
☒	☐	The statistical test(s) used AND whether they are one- or two-sided<br>*Only common tests should be described solely by name; describe more complex techniques in the Methods section.*
☒	☐	A description of all covariates tested
☒	☐	A description of any assumptions or corrections, such as tests of normality and adjustment for multiple comparisons
☐	☒	A full description of the statistical parameters including central tendency (e.g. means) or other basic estimates (e.g. regression coefficient) AND variation (e.g. standard deviation) or associated estimates of uncertainty (e.g. confidence intervals)
☒	☐	For null hypothesis testing, the test statistic (e.g. *F*, *t*, *r*) with confidence intervals, effect sizes, degrees of freedom and *P* value noted<br>*Give P values as exact values whenever suitable.*
☒	☐	For Bayesian analysis, information on the choice of priors and Markov chain Monte Carlo settings
☒	☐	For hierarchical and complex designs, identification of the appropriate level for tests and full reporting of outcomes
☒	☐	Estimates of effect sizes (e.g. Cohen's *d*, Pearson's *r*), indicating how they were calculated

Our web collection on statistics for biologists contains articles on many of the points above.

Software and code

Policy information about availability of computer code

Data collection	We did not generate any new software for our work. All software is mentioned and referenced in the Material and Methods section of the paper. Data collection software includes:<br>- NMR: Data were collected with Topspin 3.6.1 (Bruker Biospin, Germany)<br>- Analytical ultracentrifugation: Data acquisition was performed using ProteomeLab XL-I Version 6.0 (Firmware 5.7)<br>- MS analysis: Data acquisition was performed using MassLynx V4.1 software (Waters)<br>- HPLC: Data were collected with LCsolution version 1.25 SP4 (Shimadzu)<br>- HPAEC-PAD: Data acquisition was performed using Chromeleon 7.2 SR4 (Thermo Scientific)
Data analysis	We did not generate any new software for our work. All software is mentioned and referenced in the Material and Methods section of the paper. Data analysis software includes:<br>- NMR: Spectra were processed with Topspin 3.6.1 (Bruker Biospin, Germany) and analyzed with Sparky 3.115 (Goddard T.D., Kneller D.G. (2008) SPARKY 3. University of California, San Francisco, CA).<br>- Spectrophotometric assays: The manufacturer's software Gen5 V1.01.14 and GraphPad Prism 7.03 were used for data analyses (BioTek Instruments GmbH, Bad Friedrichshall/Germany)<br>- MS analysis: Spectra were analyzed using MassLynx V4.1 software (Waters). MS/MS protein spectra were automatically analyzed using the program ProteinLynx Global Server (Version 2.1, Waters)<br>- Crystallographic data: Data were integrated using XDS. The Phase Problem was solved in crank2. Native and soaked crystal data were solved by Molecular Replacement in Phaser with the gadolinium-soaked structure as a search model. Structures were refined by several cycles of automatic and manual refinement in Phenix and Coot, respectively.<br>- Analytical ultracentrifugation: Protein extinction coefficients at 280 nm, partial specific volumes, and buffer viscosities and densities were calculated from amino acid or buffer composition, respectively, by the program SEDNTERP. Sedimentation velocity data were analyzed using a model for diffusion-deconvoluted differential sedimentation coefficient distributions [continuous c(s) distributions] implemented in the program SEDFIT. Figures were prepared using the program GUSSI.

- HPLC: Data analysis was performed with LCsolution version 1.25 SP4 (Shimadzu).
- HPAEC-PAD: Data analysis was performed using Chromeleon 7.2 SR4 (Thermo Scientific).
- Bioinformatics analyses: Putative homologs of CsaC were identified using Blastp. Multiple-sequence alignments were performed with Clustal omega and annotated with Jalview, or performed with CLUSTALW implemented in MEGAX.

For manuscripts utilizing custom algorithms or software that are central to the research but not yet described in published literature, software must be made available to editors and reviewers. We strongly encourage code deposition in a community repository (e.g. GitHub). See the Nature Research guidelines for submitting code & software for further information.

Data

Policy information about availability of data

All manuscripts must include a data availability statement. This statement should provide the following information, where applicable:
- Accession codes, unique identifiers, or web links for publicly available datasets
- A list of figures that have associated raw data
- A description of any restrictions on data availability

The crystallographic datasets reported herein have been deposited in the PDB repository under accession codes 6YUO [https://doi.org/10.2210/pdb6YUO/pdb], 6YUV [https://doi.org/10.2210/pdb6YUV/pdb], 6YUS [https://doi.org/10.2210/pdb6YUS/pdb], 6YUQ [https://doi.org/10.2210/pdb6YUQ/pdb. Source data for Fig. 3, Fig. 5 and Supplementary Fig. 14 are provided with this paper. Raw NMR and mass spectrometry data are available from the corresponding authors upon request.

Field-specific reporting

Please select the one below that is the best fit for your research. If you are not sure, read the appropriate sections before making your selection.

☒ Life sciences ☐ Behavioural & social sciences ☐ Ecological, evolutionary & environmental sciences

For a reference copy of the document with all sections, see nature.com/documents/nr-reporting-summary-flat.pdf

Life sciences study design

All studies must disclose on these points even when the disclosure is negative.

Sample size	Sample sizes (i.e. number of replicate experiments) were not predetermined on statistical methods, but were choosen according to common practices in enzyme research (at least three independent experiments with excact replicate numbers given in figure legends). Statistics were limited to determination of mean values +/- SD.
Data exclusions	No data were excluded from the analyses.
Replication	Spectrophotometric and radioactive incorporation assays were performed at least three times (the exact number of repeats (n) is indicated in the figure legends). For sedimentation velocity analyses by analytical ultracentrifugation, two runs were performed with four different protein concentrations, all yielding highly similar results. Molar masses were confirmed by an independent sedimentation equilibrium experiment at four rotor speeds and two protein concentrations. The bioinformatic analysis was performed independently with two separate alogrithms, ClustalW and Clustal omega, yielding highly similar results. All attempts of replication were successful.
Randomization	Randomization was not necessary as no allocation of samples into experimental groups was required. In our experimental set up, defined enzyme variants were compared under well controlled conditions. Accordingly, the assays performed in this study did not depend on statistical analyses of an unknown relationship, but required a rational approach for activity comparison. Statistics were limited to determination of mean values +/- SD deviation.
Blinding	Blinding was not relevant because results are quantitative and did not require subjective judgment or interpretation.

Reporting for specific materials, systems and methods

We require information from authors about some types of materials, experimental systems and methods used in many studies. Here, indicate whether each material, system or method listed is relevant to your study. If you are not sure if a list item applies to your research, read the appropriate section before selecting a response.

Materials & experimental systems

n/a	Involved in the study
☒	☐ Antibodies
☒	☐ Eukaryotic cell lines
☒	☐ Palaeontology and archaeology
☒	☐ Animals and other organisms
☒	☐ Human research participants
☒	☐ Clinical data
☒	☐ Dual use research of concern

Methods

n/a	Involved in the study
☒	☐ ChIP-seq
☒	☐ Flow cytometry
☒	☐ MRI-based neuroimaging
